# Impact of in-person versus online supervised multicentre multicomponent prenatal exercise programme on maternal physical activity, fitness and healthy lifestyle: the Active Pregnancy trial SPIRIT 2025-based protocol

**DOI:** 10.1136/bmjsem-2025-002767

**Published:** 2025-07-11

**Authors:** Rita Santos-Rocha, Marco Branco, Joana Prior de Freitas, Bárbara Castro, Adelaide Teixeira Pinto, Sandra Silva - Santos, Cristina Nogueira-Silva, Miguel Angel Oviedo-Caro, Anna Szablewska, Anna Szumilewicz

**Affiliations:** 1 ESDRM Sport Sciences School of Rio Maior, Santarém Polytechnic University, Rio Maior, Portugal; 2 SPRINT Sport Physical Activity and Health Research and Innovation Center, Rio Maior, Portugal; 3 Instituto de Estudos Superiores de Fafe Lda, Braga, Portugal; 4 University of Minho School of Medicine, Braga, Portugal; 5 ICVS Institute of Life and Health Sciences, Braga, Portugal; 6 University of Seville, Seville, Spain; 7 Faculty of Health Sciences, Medical University of Gdansk, Gdansk, Poland; 8 Gdansk University of Physical Education and Sport, Gdansk, Poland

**Keywords:** Pregnancy, Exercise testing, Physical fitness, Physical activity, Women

## Abstract

Many studies have supported the positive effects of an active lifestyle on the prevention and treatment of pregnancy-related complications, as well as maintaining fitness and functionality. The COVID-19 pandemic has underscored the need for virtual approaches to health interventions; however, few studies have examined the effectiveness of these interventions. The Standard Protocol Items: Recommendations for Interventional Trials 2025 guidelines were followed to describe the protocol of the ACTIVE PREGNANCY trial, an intervention study that delivers a physical exercise programme in-person (IN) and online (ON) to improve maternal physical activity (PA), fitness and healthy lifestyle parameters in pregnant women. Pregnant women will be invited to participate in a multisite, multicomponent exercise programme delivered either IN or ON by qualified exercise physiologists. Both groups will receive an exercise intervention delivered in different real-life environments. Participants will undergo basic fitness field tests and complete questionnaires assessing PA, fitness and lifestyle parameters before starting the exercise programme and after completing 12 weeks of the intervention. Researchers will test whether the intervention is beneficial in maintaining or improving maternal parameters after 12 weeks and will compare the effectiveness of different exercise modes. Subgroups of maternal age and weekly volume of PA will also be analysed.

Primary outcomes: PA volume and health-related and functional fitness. Secondary outcomes: healthy lifestyle parameters. Additional outcomes: satisfaction with the exercise interventions and resources. This study was approved by the Ethics Committee of Santarém Polytechnic University and registered on ClinicalTrials.gov (NCT06954454). Findings will be disseminated via publications, conferences and training programmes.

**WHAT IS ALREADY KNOWN ON THIS TOPIC**

- Exercise during pregnancy provides various benefits for maternal health and cardiorespiratory fitness.
- The impact of exercise on the other health-related and skill-related fitness components is poorly explored.
- The effectiveness of supervised virtual interventions is poorly understood.

**WHAT THIS STUDY ADDS**

- This is the first study addressing the effectiveness of virtual intervention with pregnant women.
- The study will provide novel insights regarding the main maternal fitness components.
- The study covers a comprehensive analysis of health-related and skill-related fitness components.

**HOW THIS STUDY MIGHT AFFECT RESEARCH, PRACTICE OR POLICY**

- The results of the present work will be useful to test and compare the effectiveness of the in-person and online exercise programmes on maternal health and fitness during pregnancy.
- The findings will provide support to healthcare professionals in promoting physical activity and exercise during pregnancy and to exercise professionals in delivering safe and effective programmes.
- The results will inform future exercise recommendations and practice regarding virtual training.
- The results will inform public health strategies for promoting active and healthy lifestyles during pregnancy regardless of maternal age, health status and fitness levels.
- This study could pave the way for targeted interventions incorporating prenatal exercise as a prevention strategy of pregnancy-related complications by promoting an active and healthy lifestyle.

## Introduction

### Background and rationale

 Many studies have been supporting the positive effects of an active lifestyle concerning the prevention and treatment of pregnancy-related complications, such as gestational diabetes, hypertension, obesity, low back pain, urinary incontinence and depression, as well as regarding its effectiveness in maintaining fitness and functionality and improving postpartum recovery. Therefore, maternal physical activity (PA) is a significant public health issue.^1^ On the other hand, physical fitness is considered a powerful marker of health, as it is associated with a lower risk of cardiovascular events, cancer and all-cause mortality across all ages.^2 3^ Moreover, some studies with pregnant women have highlighted the potential impact of higher physical fitness levels on improved maternal and foetal health.^4^,^6^

Practice guidelines have become increasingly popular tools for synthesising evidence-based information to assist practitioners and patients in making decisions related to starting or continuing PA. Updated guidelines for exercising during pregnancy have been issued in recent years^7^,^9^ to facilitate informed advice from healthcare providers. Despite this knowledge, most women still do not receive proper guidance on how to exercise during pregnancy or after childbirth, and the prevalence of physical inactivity is still high among pregnant women.

The isolation periods during the COVID-19 pandemic may have harmed the health and fitness of pregnant women and introduced various PA dilemmas by experiencing disruption in their access to obstetric healthcare.^10^ This pandemic has accentuated the need for virtual interaction with populations considered to be at greater risk, such as pregnant women.^11^ Virtual interaction has increased worldwide, and its advantage is that it enables reaching remote and excluded populations, emphasising online programme delivery. Few studies have been conducted so far showing positive effects on selected pregnancy outcomes, such as birth weight and depression,^12 13^ but there is a lack of studies supporting the effectiveness of virtual interactions on general health and fitness. Moreover, the relative efficacy of these interventions on fitness parameters has not been compared in different environments.

We hypothesised that a multicomponent supervised prenatal physical exercise programme previously described in Santos-Rocha *et al*’s study^14^ positively impacts PA, fitness, lifestyle and health parameters of women throughout pregnancy, regardless of the delivery environment (in-person (IN) *vs* online (ON)), maternal age or weekly volume of PA, and that both exercise environments are equally effective.

### Objectives

To describe the study protocol of the ACTIVE PREGNANCY trial following the updated Standard Protocol Items: Recommendations for Interventional Trials 2025 guidelines,^15^ which is a supervised multicomponent prenatal exercise programme delivered either IN or ON aimed at improving maternal PA levels, health-related and functional fitness, healthy lifestyle and health parameters throughout pregnancy, regardless of maternal age or baseline PA. The primary objectives are to compare the impact of IN *versus* ON interventions on PA levels, self-perceived fitness and health-related and functional fitness. The secondary objectives are to assess the impact of both interventions on lifestyle (ie, Mediterranean diet, sleep quality, happiness and depression) and health parameters (ie, weight gain, gestational diabetes or hypertension) and to analyse differences by maternal age subgroups (<35 *vs* 35+years) and weekly volume of PA (<150, 150–300 or 300+min/week). Additional objectives include exploring participant satisfaction with the exercise interventions and evaluating the usefulness of digital resources (ie, ACTIVE PREGNANCY Guide, YouTube channel ACTIVE PREGNANCY and the framework of the ACTIVE PREGNANCY app).

## Methods

### Patient and public involvement

Participants in previous pilot studies were consulted regarding their satisfaction with the exercise intervention and the characteristics of the exercise professionals.^16^ These inputs have been used to improve the quality of the interventions. Medical doctors from participating hospitals will collaborate with the research group in recruiting participants, disseminating research findings through publications and seminars and delivering relevant educational content.

### Trial design

This is a multicentre, two-arm, parallel-group, randomised controlled trial (RCT) with a 1:1 allocation ratio and an equivalence framework. Pregnant women will be randomly assigned to either an IN or ON supervised multicomponent exercise intervention.

### Trial setting

The study will be conducted in healthcare and fitness centres across participating hospitals and universities in Portugal.

### Participants

This study aims to include healthy pregnant women (uniparous and multiparous) who, after the first clinical visit and ultrasonically confirmed viable intrauterine pregnancy, will be randomly allocated to one of two groups: exercise intervention delivered IN or ON, after signing an informed consent form.

### Eligibility criteria for participants

Inclusion criteria: pregnant women aged 18–50 years; 13–20 weeks of gestation at recruitment; no absolute contraindications to exercise during pregnancy; attending healthcare services regarding pregnancy and understanding the Portuguese language.

Exclusion criteria: medical conditions contraindicating PA and inability to participate in IN or ON formats.

### Eligibility criteria for exercise professionals who will deliver the interventions

The exercise intervention will be carried out by graduated exercise professionals (EQF level 6 or 7), holders of a BSc, MSc or PhD in Exercise or Sport Sciences, with specialisation in exercise testing and prescription during pregnancy. Exercise professionals will explain the study’s objectives and obtain informed consent before conducting assessments and delivering the exercise intervention.

### Exercise intervention programmes and comparators

The interventions follow international guidelines for PA during pregnancy.^7^,^9^ The exercise programme was designed and validated as a complex intervention and underwent three stages: development, pilot testing and evaluation.^14^ Exercise sessions consist of a combination of aerobic, resistance, stretching, balance, coordination, postural and pelvic floor muscle exercises, using multicomponent activities, such as aerobics, dancing, step, Pilates, bar, chair and resistance exercises using elastic bands, exercise balls, mats, dumbbells or body weight.

Table 1 describes both arms of the exercise intervention in line with the American College of Sports Medicine exercise prescription guidelines.^17^

**Table 1:** Description of both arms of the exercise intervention

Arm 1:IN exercise group	Type of exercise: multicomponent prenatal exercise programme, including aerobic, coordination, strength, stretching, balance, postural and pelvic floor muscle training exercises.Duration and frequency: two sessions of 60 min per week for 12 weeks.Intensity: moderate. According to the Borg scale of perceived exertion, which ranges from 6 to 20, moderate exercise is rated between 13 and 14. Each pregnant woman will rate her perceived exertion on a scale of 6–20.IN supervision: qualified exercise physiologists.Setting: healthcare, university or fitness centres.
Arm 2:ON exercise group	Type of exercise: multicomponent prenatal exercise programme, including aerobic, coordination, strength, stretching, balance, postural and pelvic floor muscle training exercises.Duration and frequency: two sessions of 60 min per week for 12 weeks.Intensity: moderate. According to the Borg scale of perceived exertion, which ranges from 6 to 20, moderate exercise is rated between 13 and 14. Each pregnant woman will rate her perceived exertion on a scale of 6–20.Remote supervision using a virtual platform: qualified exercise physiologists.Setting: home.
	Examples of the exercise programme are continuously updated on the ACTIVE PREGNANCY YouTube channel:@GravidezAtiva-ActivePregnancy(https://www.youtube.com/channel/UC0Vyookwc0mcQ5T70imtoNA/playlists).

IN, in-person; ON, online.

### Additional materials describing the intervention

Additional recommendations to participants include open-access educational materials, such as the ACTIVE PREGNANCY Guide (online supplemental 1), walking, swimming or performing a video session from the ACTIVE PREGNANCY YouTube Channel on other days of the week.

Exercise physiologists delivering the intervention will be supported by a specific textbook^18^ and the ACTIVE PREGNANCY YouTube Channel regarding programme planning, exercise testing and prescription and exercise adaptations.

Healthcare professionals providing health screenings and recruiting participants will be supported by the Health Professionals Guide (online supplemental 2).

Individual reports will be provided for each participant and her healthcare provider.

### Explanation for the choice of comparators

Despite the existing evidence on prenatal exercise, there is a need for robust, multicentre studies evaluating the impact of both IN and ON exercise interventions on maternal PA, fitness and health outcomes, independent of maternal age, health status or baseline fitness.

### Criteria for discontinuing or modifying allocated interventions

Participants will be free to discontinue or modify allocated interventions on request or due to medical reasons without any consequences.

### Strategies to improve adherence to interventions

Exercise professionals will monitor adherence to the intervention by recording the number of sessions attended over 12 weeks. Motivational strategies include organising online group meetings, sending weekly WhatsApp and Instagram reminders, providing access to resources promoting an active and healthy lifestyle and offering access to the YouTube channel.

The overall focus of these sessions is based on what already motivates participants to increase or maintain their PA level and healthy lifestyle. This is achieved by fostering interaction among participants to create meaningful group processes, such as support, experience exchange and reflection, as well as collecting feedback regarding the exercise programme and resources. All monthly online sessions will last 20–30 min and are led by exercise and health professionals. These sessions aim: (1) to inform about PA guidelines, benefits associated with PA and possible ways to increase PA during pregnancy; (2) to discuss participant’s barriers, wishes, needs, knowledge and former PA experiences to identify individual characteristics and motivation towards a more physically active lifestyle; (3) to teach the advantages of using a PA tracker, aside from measuring the PA level, the activity trackers are also used as an intervention element to motivate the participants to increase their PA levels, share it on a Strava group and provide feedback on recent PA performances; (4) to collect feedback regarding the organisation of the exercise programme and (5) to discuss other relevant topics chosen by the participants, such as myths about pregnancy PA, postpartum PA, the pelvic floor, uterine contractions, abdominal muscles and diastasis recti, birth, etc.

Individual sessions will be scheduled during the daytime at a time that is as convenient as possible for the participant.

The primary objectives of these motivational strategies are to enhance participant retention and adherence to follow-up, as well as to incorporate a patient-centred approach into the exercise intervention.

### Concomitant care permitted or prohibited during the trial

During the exercise trial, participants will be permitted to continue their routine medical care, including medications and consultations with their primary healthcare providers, as well as maintain their usual PA and nutritional habits, such as participation in other organised exercise programmes or specific sports.

### Timeline

**Figure 1:**
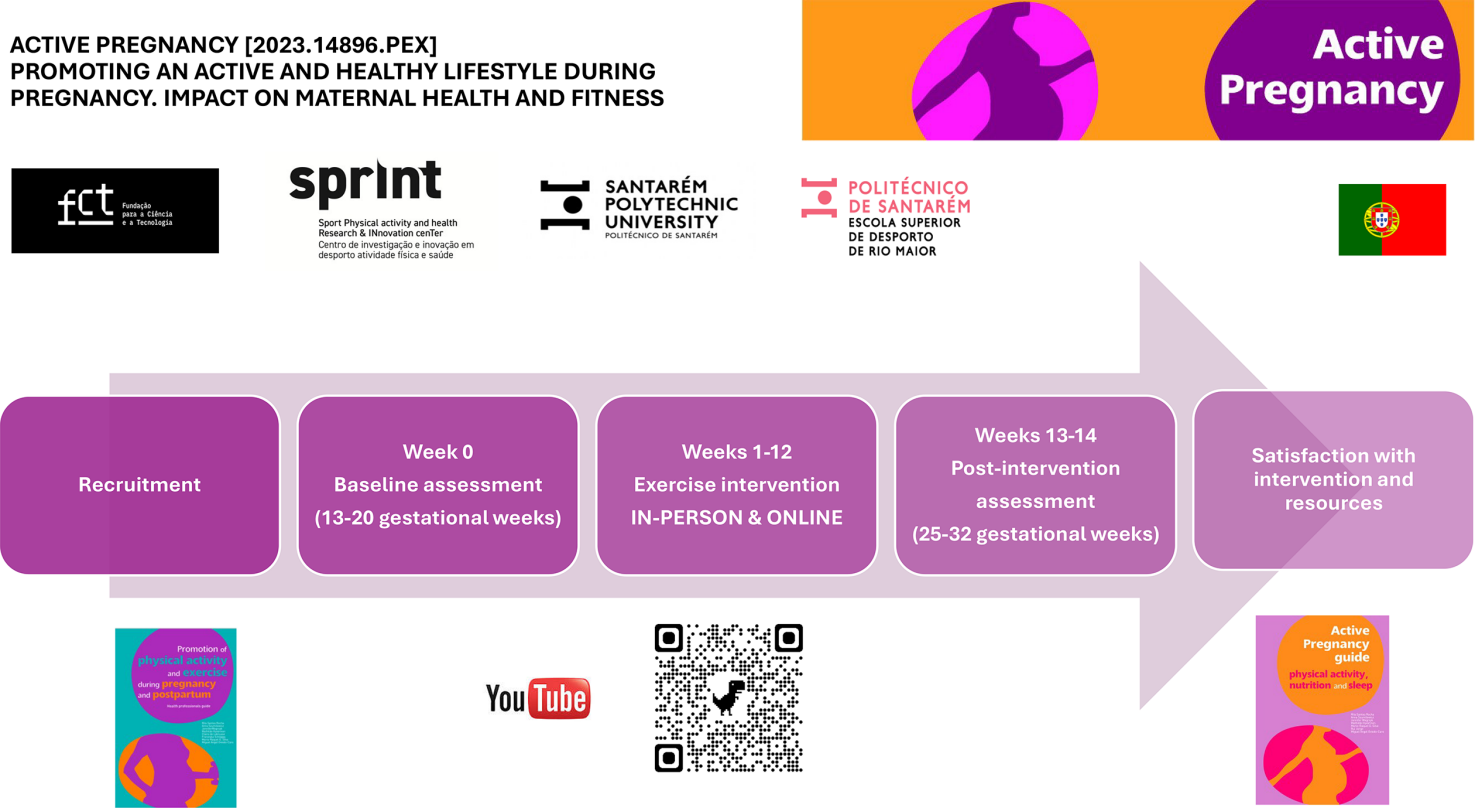
Study’s timeline.

The schematic diagram of the study, including the schedule of enrolment, data collection points (assessments) at baseline (13–20 gestational weeks) and postintervention (25–32 gestational weeks), interventions and additional collection points, is shown in figures1  2.

**Figure 2:**
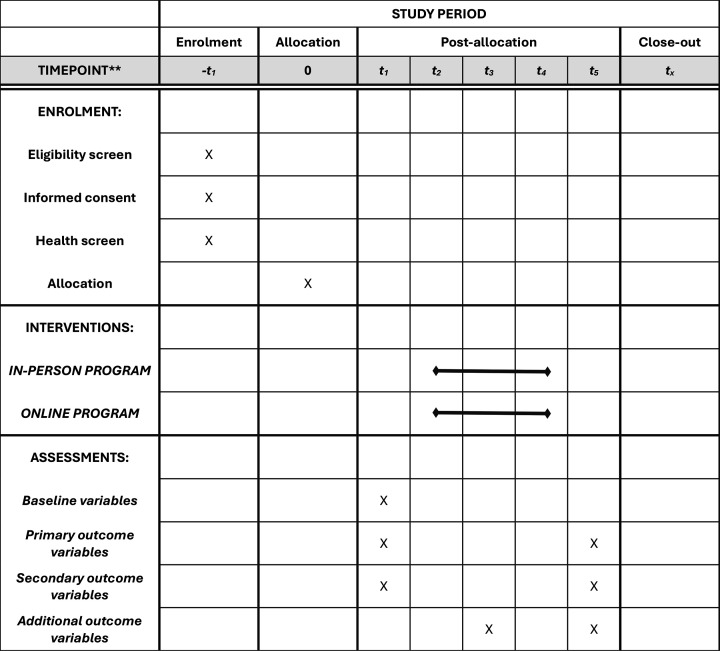
Schedule of enrolment, interventions and assessments. −t1, enrolment week; t0, allocation and baseline week; t1, pretraining assessment; t3, post-training assessment (week 12).

### Outcomes

Primary, secondary and additional outcome measures, including the specific measurement variables, timeframe and instruments, are shown in table 2.

**Table 2:** Primary, secondary and additional outcome measures, including the specific measurement variables, timeframe and instruments

Outcome measures	Instruments
Primary outcome measuresTimeframe: baseline (week 0) and after 12-week intervention (weeks 13 and 14)Analysis metric: change from baseline	
PA level and pattern of PA in the type of PA (work, active commuting, exercise, etc.), type of exercise, duration of each session (time spent in PA), weekly frequency and intensity and weekly volume (MET-min/week).	PPAQ^21^ and get active questionnaire for pregnancy^22^
Self-perceived fitness: self-perceived global fitness and fitness components (cardiorespiratory, strength, speed/agility and flexibility) when compared with other women of the same age. Likert scale 1 (poor) to 5 (very good).	IFIS^23^
Cardiorespiratory fitness estimated in mL/kg/min	Cardiorespiratory fitness—6-min walk test (average distance covered in metres and estimated cardiorespiratory fitness in mL/kg/min)^24^https://youtu.be/CUaFgfdSObc
Upper limbs strength	Upper limb strength—wall push-ups (number of repetitions in 30 s)^6 17^https://youtu.be/dp_tRoyxVYU
Lower limbs strength	Lower limb strength—sit-and-stand (number of repetitions in 30 s)^6 17 25^https://youtu.be/3ihcqnzyTXk
Dynamic balance	Dynamic balance—superwoman^6 17^https://youtu.be/Xiv_vWpe4-A
Static balance	Static balance—one-leg stance^6 17^https://youtu.be/sr0eYCfbLiM
Lower limbs flexibility	Lower limbs flexibility—modified sit and reach (mm)^6 17^https://youtu.be/Y6y_FAa3ulk
Upper limbs flexibility	Upper limbs flexibility—back scratch (mm)^6 17^https://youtu.be/MBwKi8kSFx8
Agility	Agility—time up and go over 3-m distance (in min)^6 17^https://youtu.be/3C944cMuTFo
Secondary outcome measuresTimeframe: baseline (week 0) and after 12-week intervention (weeks 13 and 14)Analysis metric: change from baseline	
Sleep quality	PSQI^26^
Weight prepregnancy, weight at baseline and weight gain (kg)	Body weight: measure with the participant wearing light clothing and without shoes^27^
Happiness scale	SHS^28^
Depression scale	EPDS^29^
Adherence to the Mediterranean diet	MEDAS^30^
HR	Measured rest HR, estimated maximal HR and calculated target HR (beats per minute)
Prevalence of pregnancy-related symptoms and health parameters (eg, weight gain, blood pressure, prevalence of gestational diabetes or hypertension and contraindications for PA)	Medical records, pregnancy symptoms inventory and get active questionnaire for pregnancy^22^
Other prespecified outcome measuresTimeframe: after the intervention (13 and 14 weeks)	
Participant satisfaction with the available resources—Likert scale (1–5, with 1 not satisfied at all, and 5 highly satisfied).	Satisfaction with educational digital resources (ie, ACTIVE PREGNANCY Guide, YouTube channel ACTIVE PREGNANCY and the framework of the ACTIVE PREGNANCY app).
Participant satisfaction with the intervention—Likert scale (1–5, with 1 not satisfied at all, and 5 highly satisfied).	A questionnaire explicitly made for the intervention evaluating different domains: type of exercises, hardness of the sessions, difficulty in particular exercises, weekly frequency, duration of the sessions, context of intervention (online, outdoor, gym and hospital), satisfaction with the exercise physiologist, willingness to continue in postpartum, willingness to repeat in future pregnancy and overall satisfaction to be included in the programme.
Feasibility and adherence	Feasibility evaluation of the implementation of the exercise programme: (1) number of women who declined to participate in the study; (2) number of women who gave up participation in the study; (3) adherence to the programme (number of days of actual performance of the exercise programme while admitted) and (4) number of women who completed at least 80% of the study.

EPDS, Edinburgh postpartum depression scale; HR, heart rate; IFIS, International Fitness Scale; MEDAS, Mediterranean Diet Adherence Screener; MET, metabolic equivalent; PA, physical activity; PPAQ, pregnancy physical activity questionnaire; PSQI, Pittsburgh sleep quality index; SHS, subjective happiness scale.

### Harms

Any spontaneously reported adverse events and other unintended effects of trial interventions or health status changes (absolute contraindication for PA) should be immediately reported by the participants, the exercise or the healthcare professionals to the principal researcher.

### Sample size

The sample size was determined using G*Power software (V.3.1) based on previous studies. To detect statistical differences between pre- and postdifferences in one group, a sample of 36 pregnant women was considered based on calculations performed using G*Power with the inputs of the mean±SD from previous studies by Dennis *et al*,^19^ a power of 0.8 and α≤0.05. Additionally, to detect statistical differences between two independent groups of pregnant women included (experimental) or not (control) in exercise programmes, a sample size of 59 per group was calculated based on the mean±SD values from previous studies by Ramírez-Vélez *et al*,^20^ a power of 0.8 and α≤0.05. Based on an expected loss-to-follow-up rate of 20%, we plan to include 100 participants in each of the two intervention groups. Therefore, a total of 200 pregnant women will be recruited and randomly allocated to either the IN or ON group (100 per group).

### Recruitment

Recruitment will occur through healthcare centres or hospitals during the first prenatal visit, as well as through social media campaigns and community outreach. Standard care consists of three appointments with their general practitioner or obstetrician (gestational weeks 6–10, 25 and 32), including ultrasonic scans. After the first visit, the woman will be verbally informed about the study by the healthcare professional. An informative flyer and the ACTIVE PREGNANCY Guide (online supplemental 1) will be provided, and written informed consent will be obtained to refer her to an exercise professional participating in the project. The exercise professional will provide further details on the intervention and objectives of the study, and allocation will be concealed to one of the IN or ON groups (figure 1) after checking the inclusion criteria and signing an informed consent. After inclusion, anthropometric and demographic information will be collected, along with a brief interview with the participant. The interview will provide insight into the participant’s thoughts on participating in a research project, as well as their prior and current PA levels, exercise preferences and experiences with health technologies to incorporate a patient-oriented approach into the exercise intervention. Before starting the intervention, the baseline data of the participant are measured.

### Assignment of interventions, randomisation and blinding

Blinding will be used by healthcare providers who recruit women and refer them to one of the available programmes. Exercise physiologists will generate a simple random allocation sequence stratified by study sites. Participants, healthcare providers and outcome assessors will not be blinded after assignment to interventions. Data analysts will be blinded after assignment to interventions.

### Data collection methods

Data will be collected through online (Google Forms) reliable and validated questionnaires, as well as via IN or ON field test assessments. Training for assessors will be provided to promote data quality. Table 1 includes the description of trial instruments. Data collection forms will be accessed by assessors, principal investigators (PIs) and data analysts. Data will be securely stored and managed following established ethical guidelines. Data from participants who do not complete at least 80% of the scheduled sessions will be considered a loss to follow-up.

### Plans to promote participant retention and complete follow-up

Plans to promote retention include the above-mentioned motivational strategies. Moreover, they will be informed about the study’s objectives and dissemination plans. Participants who discontinue or deviate from intervention protocols will be contacted by telephone to provide qualitative feedback and help us understand context-specific issues.

### Trial monitoring

The steering group of the research team comprises the PI and senior researchers who participated in planning and will provide organisational support to the trial by involving and supervising students, as well as organising scientific events related to the project. This group will meet every other month. The PI and junior researchers of the team will lead the trial. This group will meet every other week. The PI will be responsible for monitoring trial conduct.

### Data management

All data entry will be performed exclusively in electronic format by the exercise professionals. Data coding will be performed using the nine digits of the national VAT number (eg, 123456789). Data quality is checked (eg, missing data, duplicate data entry and range checks for data values) and monitored by the PI and data analysts. Data analysis will be performed by the data monitoring group, which includes PI, assessors and data analysts. Its main role is to check data quality. This group is independent from the sponsor and has no competing interests.

Access to the full protocol, participant-level data and statistical code can be provided by the PI on request and justification.

Data will be analysed according to the intent-to-treat principle and presented following the guidelines of the International Consolidated Test Reporting Standards guidelines 2025.

### Statistical methods for primary and secondary outcomes

Demographic data (age, weight, gestational age, area of residence and date of birth) will be described using frequencies and percentages for categorical variables.

Descriptive statistics (mean, SD, median and IQR) will be used to summarise baseline characteristics of participants in both groups (IN and ON).

Descriptive and inferential statistics will be applied to all outcomes.

A comparative analysis of IN *versus* ON interventions will be conducted using both intention-to-treat and per-protocol approaches. Comparative analysis will be used to compare changes in primary and secondary outcomes between IN and ON groups over time (baseline and postintervention). Subgroup analyses will evaluate potential differences between maternal age groups (<35 *vs* 35+ years) and PA level groups.

Adherence to the intervention (proportion of attendance in the planned sessions) will be calculated after the postintervention assessments.

Data from participants who do not complete at least 80% of the scheduled sessions will be considered a loss to follow-up.

Regarding additional outcome analysis, the evaluation of interventions and resources will be performed through descriptive analysis, summarising satisfaction with resources and exercise interventions.

Continuous variables will be assessed for normality using Shapiro–Wilk tests. Normally distributed variables will be presented as mean±SD and analysed using the independent sample t-test. The non-normally distributed data will be reported as the median with IQR and will be analysed using the Mann–Whitney test. For the within-group difference, normally distributed variables will be calculated with the paired-sample t-test. In contrast, those non-normally distributed variables will be calculated with the paired-sample non-parametric Wilcoxon test. Categorical data will be analysed using Pearson’s χ^2^ test or Fisher’s exact test, as appropriate.

A significance level of 0.05 (alpha level) will be set for all statistical tests. Power calculations were performed to ensure that the study was adequately powered to detect clinically significant differences between groups. All statistical analyses will be conducted in Statistical Package for the Social Sciences.

### Interim analysis

An interim analysis will be performed by the steering group and provided to the research group every 3 months until a final decision is made to terminate the trial.

### Post-trial care

After completing 12 weeks of trial participation, including baseline and postintervention assessments, participants will be invited to continue for free until the gestational week of childbirth. They will continue to have access to educational materials after the study ends. Participants will have access to ongoing healthcare and will be invited to participate in a similar trial during the postpartum period.

## Discussion

The ACTIVE PREGNANCY trial is an equivalence RCT with two parallel exercise intervention groups based on a multisetting, multicomponent, customised, supervised prenatal exercise programme.

The study is expected to provide evidence on the effectiveness of supervised prenatal exercise programmes delivered IN or ON on PA, fitness, lifestyle and health parameters of pregnant women. The findings will contribute to exercise prescription guidelines, social prescription programmes and policy recommendations aimed at promoting maternal health during pregnancy. Moreover, this study will generate evidence on how to incorporate exercise into everyday life for pregnant women.

Previous studies have investigated the effects of various IN exercise interventions on health outcomes in pregnant women. However, none of these studies have focused primarily on investigating the effect of exercise interventions on actual PA levels and age groups.

The literature is scarce regarding the effects of such interventions on health-related and skill-related fitness components, while numerous non-validated tools are used for fitness assessment.^6^ Moreover, the literature is scarce regarding the effectiveness of virtual interventions on health and fitness parameters. Thus, the main purpose of the ACTIVE PREGNANCY trial is to compare the effect of two different contexts of intervention.

Additionally, the ACTIVE PREGNANCY trial includes a process evaluation of the exercise intervention to enhance adherence. It explores the acceptability of resources promoting active and healthy lifestyles supported by health technology.

### Limitations of the study

A limitation of this ecological study is that PA and health parameters data will be collected through questionnaires, and fitness parameters data will be collected through field tests. PA and fitness parameters will be extensively measured using two different methods (questionnaires and field tests). Still, data will not be obtained by gold standard methods, such as doubly labelled water and direct oxygen measurements, respectively. Moreover, there are no validated batteries of specific tests for pregnant women. Another limitation is that, although technology is an integral part of modern living, online exercise interventions and digital resources require participants to use a personal computer or mobile phone, which can be expensive for some populations. Another limitation is the number of assessments, which require organisational adaptations to avoid burdening the participants and challenge adherence and compliance.

### Strengths of this study

The trial is comprehensive and multidisciplinary in its design and methodologies.

The efficacy of structured, supervised exercise training, provided in both IN and ON environments, using active and healthy lifestyle promotion resources, is directly compared in an RCT to improve multidimensional PA, fitness, lifestyle and health parameters in pregnant women.

The trial involves complex interventions provided in both IN and ON environments and is conducted in multiple settings across different locations in Portugal to enhance generalisability. In addition to the ecological assessment of multidimensional parameters of PA and health, this study will also extensively cover health-related and skill-related fitness parameters in pregnant women. Yet, as one of the additional outcomes, a process evaluation is conducted alongside the trial to explore how the interventions are carried out and adapted. Moreover, the stakeholders will also assess the resources for promoting active and healthy lifestyles. These resources are available in other languages. Therefore, the protocol may be developed in other countries.

The study will provide evidence-based knowledge that can contribute to improving recommendations for PA during pregnancy and to developing new, effective and simple guidance for implementing health technology-supported exercise programmes for pregnant women.

### Dissemination plans

Plans to disseminate trial results to participants, exercise and healthcare professionals, the public and other relevant groups include reporting in the trial registry, plain language summary and social media communications, plain language reports to communicate trial results to participants, publication of study protocol and findings in open access journals, academic dissertations or thesis, national and international conferences for exercise and health professionals and policy briefs.

Based on the results and process evaluation, the knowledge and tools from the ACTIVE PREGNANCY study can be transformed into initiatives in municipalities and hospitals to promote active and healthy lifestyles for both mothers and children. They can be used to prevent the development of lifestyle-related diseases across generations.

### Trial status

Recruiting start date: May 2025. Expected completion date: December 2026.

## Supplementary material

10.1136/bmjsem-2025-002767 online supplemental file 1

10.1136/bmjsem-2025-002767 online supplemental file 2

10.1136/bmjsem-2025-002767 Abstract translation 1 This web only file has been produced by the BMJ Publishing Group from an electronic file supplied by the author(s) and has not been edited for content.
