# Supplementary material for: Impact of in-person versus online supervised multicentre multicomponent prenatal exercise programme on maternal physical activity, fitness and healthy lifestyle: the Active Pregnancy trial SPIRIT 2025-based protocol
Source: BMJ Open Sport Exerc Med. 2025 Jul 11;11(3):e002767. doi: 10.1136/bmjsem-2025-002767 (PMC12248212; doi:10.1136/bmjsem-2025-002767)
Supplement: online supplemental file 1 [file bmjsem-11-3-s001.pdf]

# Active Pregnancy guide

## physical activity, nutrition and sleep


Rita Santos-Rocha
Anna Szumilewicz
Jennifer Wegrzyk
Mathilde Hyvärinen
Maria-Raquel G. Silva
Rui Jorge
Miguel Ángel Oviedo-Caro


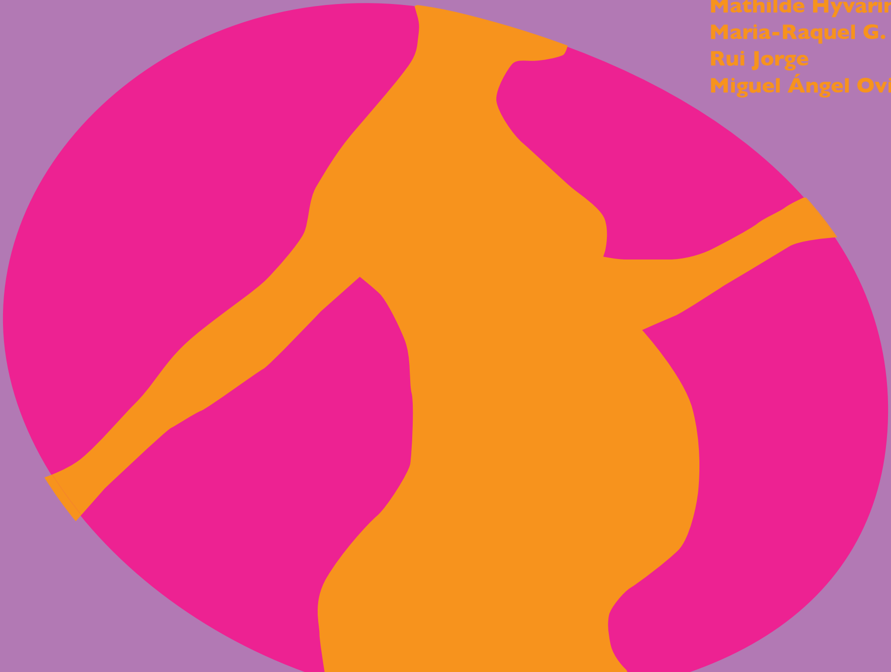

**ACTIVE PREGNANCY GUIDE - Physical activity, nutrition, and sleep**

Rita Santos-Rocha[1], Anna Szumilewicz[2], Jennifer Wegrzyk[3], Mathilde Hyvärinen[4], Maria-Raquel G. Silva[5], Rui Jorge[6], Miguel Ángel Oviedo-Caro[7]

**ISBN** 978-989-8768-50-6

**Publisher** Instituto Politécnico de Santarém - Escola Superior de Desporto de Rio Maior

**Cover and layout** SAF Design Works

**2023**

[1] R. Santos-Rocha, PhD

ESDRM-IPSANTARÉM Sport Sciences School of Rio Maior, Polytechnic Institute of Santarém, Rio Maior, Portugal

CIPER Laboratory of Biomechanics and Functional Morphology, Interdisciplinary Centre for the Study of Human Performance, Faculty of Human Kinetics, University of Lisbon, Cruz Quebrada-Dafundo, Portugal

e-mail: ritasantosrocha@esdrm.ipsantarem.pt / ritasantosrocha@gmail.com

[2] A. Szumilewicz, PhD

Gdansk University of Physical Education and Sport, Gdańsk, Poland

e-mail: anna.szumilewicz@awf.gda.pl / anna.szumilewicz@gmail.com

[3] J. Wegrzyk, PhD

HESAV School of Health Sciences, HES-SO University of Applied Sciences and Arts Western Switzerland, Lausanne, Switzerland

e-mail: jennifer.masset@hesav.ch

[4] M. Hyvärinen, MSc

HESAV School of Health Sciences, HES-SO University of Applied Sciences and Arts Western Switzerland, Lausanne, Switzerland

e-mail: mathilde.hyvaerinen@hesav.ch

[5] M.-R.G. Silva, PhD

Faculty of Health Sciences and FP-I3ID, University Fernando Pessoa, Porto, Portugal
CIAS-Research Centre for Anthropology and Health – Group of Human Biology, Health and Society, University of Coimbra, Coimbra, Portugal

CHRC-Comprehensive Health Research Centre - Group of Sleep, Chronobiology and Sleep Disorders, Nova Medical School, Nova University of Lisbon, Lisbon, Portugal

e-mail: raquel@ufp.edu.pt

[6] R. Jorge, PhD

ESAS-IPSANTARÉM School of Agriculture / ESDRM-IPSANTARÉM Sport Sciences School of Rio Maior, Polytechnic Institute of Santarém, Santarém, Portugal

e-mail: rui.jorge@esa.ipsantarem.pt

[7] M.A. Oviedo-Caro, PhD

Department of Physical Education and Sport, University of Seville, Seville, Spain

e-mail: maovicar@gmail.com

# Contents


**Foreword 4**

**1. Benefits of physical activity during pregnancy 6**

**2. Recommendations for physical activity during pregnancy 8**

**3. Health screening and pre-exercise assessment 11**

Health screening 11

Contraindications for physical activity in pregnancy 12

Evaluation of physical activity level and setting of objectives 14

Previously sedentary women 19

Partially active and active women 19

Very active women and athletes 19

**4. Exercise testing during pregnancy 20**

**5. Exercise prescription for pregnant women 23**

Types of physical exercises 27

Exercise duration, frequency, and intensity (volume) 28

Exercise progression and adaptation to body changes 29

First trimester 30

Second trimester 30

Third trimester 31

**6. Exercise prescription in special conditions 32**

Gestational diabetes 32

Excess weight and obesity 33

Hypertension and preeclampsia 34

Low back pain 35

# Contents


Depression and mental disorders 35

Urinary incontinence 36

**7. Safety issues regarding exercise during pregnancy 37**

Falls and injury 37

Nausea and dizziness 38

Heat, humidity, and environment 39

Sportswear and shoes 39

**8. Recommendations to reduce time spent in sedentary behavior 40**

**9. Diet and nutritional recommendations during pregnancy 42**

Before pregnancy 42

Weight management during pregnancy 43

Diet and nutrition during pregnancy 44

Hydration 47

Food safety during pregnancy 49

**10. Sleep recommendations during pregnancy 50**

**References** 52

# Foreword

Physical Activity and Exercise should be part of an active lifestyle during pregnancy and the postpartum period, as shown by growing evidence on its health benefits for pregnant women and newborns. Currently, there is consensus that maintaining light to vigorous physical activity during an uncomplicated pregnancy has several benefits for the health of the woman and the fetus. The World Health Organization (WHO) recommends that "women who, before pregnancy, habitually engaged in vigorous-intensity aerobic activity or who were physically active, can continue these activities during pregnancy and the postpartum period." [1]. Thus, pregnancy and the postpartum period provide good opportunities for promoting women's health and an active and healthy lifestyle, including proper nutrition and sleep patterns.

The purpose of this ACTIVE PREGNANCY GUIDE is to provide pregnant women with a basic understanding of the importance of an active and healthy lifestyle during the different stages of pregnancy.

A general overview, further discussion, and update of the recent guidelines and practice and evidence-based knowledge supporting the topics addressed in this guide are provided in another publication [2].

Before reading this Active Pregnancy Guide, it is important to understand the following definitions [3-5]:

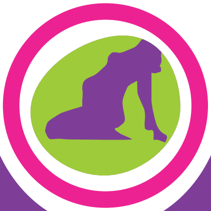

Physical activity is defined as any bodily movement produced by the contraction of skeletal muscles that results in a substantial increase in caloric requirements over resting energy expenditure. Physical activity can be categorized either by different contexts, such as leisure-time, exercise, sports, occupational, household, and transportation activities, or by intensity, i.e., light (less than 3 METs - metabolic equivalents[8]), moderate (between 3 and 5.9 METs), and vigorous (6 METs or more).

Sedentary behavior involves activities of less than 1.5 METs, including desk-based office work, driving a car and watching television.

Exercise is a type of physical activity consisting of planned, structured, and repetitive body movement to improve and/or maintain one or more components of physical fitness. Exercise is a subcategory of physical activity. Although energy expenditure is increased during physical activity, it does not necessarily reflect exercise, and should not be confused with fitness.

Physical fitness is defined as a set of attributes or characteristics related to the ability to perform physical activity. These characteristics are usually classified into health-related and skill-related components of physical fitness. According to the American College of Sport Medicine (ACSM), health-related physical fitness components include cardiorespiratory endurance, body composition, muscular strength and endurance, and flexibility, while skill-related components of physical fitness include agility, coordination, balance, power, reaction time, and speed.

Physical inactivity is a behavioral state of not achieving a certain minimum standard of physical activity on a regular basis, i.e., failing to meet the WHO recommended level of physical activity.

Exercise prescription commonly refers to a specific exercise program designed for a concrete purpose and often developed by an exercise or rehabilitation specialist for the customer. An ideal exercise program should meet individual fitness goals according to endurance, strength, flexibility, balance, coordination, gait, agility, and proprioceptive training, and respective stages of life (such as pregnancy) and clinical conditions.

[8] MET = Multiple of resting metabolic rate, used as a measure of exercise intensity.

# BENEFITS OF PHYSICAL ACTIVITY DURING PREGNANCY

# 1

Physical activity is associated with health benefits during pregnancy, delivery and postpartum period. In the last three decades, positive effects of prenatal physical activity on maternal and fetal health, and pregnancy outcomes have been highlighted. Yet, insufficient levels of physical activity have been stated worldwide. Physical inactivity is the fourth leading cause of death worldwide [6] and the strongest public health concern of the 21st century [7]. Physical inactivity during pregnancy is a significant public health9 issue due to its prevalence and association with adverse pregnancy and birth outcomes, as well as, short- and long-term risks for chronic diseases for mother and child.

Current research suggests that healthy pregnant women can begin or maintain moderate intensity aerobic exercise programs with no risk of adverse effects on their unborn fetus [8]. The role of the health care provider is also to update pregnant women on this knowledge.

Recent systematic reviews show strong evidence on the effectiveness of (moderate to vigorous intensity) physical activity on:

[9] Public health encompasses many disciplines that promote health and prevent disease and disability in defined populations.

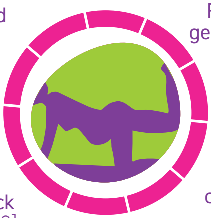


Moreover, moderate evidence suggests no effect of exercise on Apgar score [28], and no evidence shows an association between leisure-time physical activity or exercise, and an increased risk of preterm birth [28].

Further evidence-based beneficial effects have been stated for: weight retention/weight loss; breastfeeding; musculoskeletal complaints; infant neuronal development and long-term development of Non-Communicable Diseases (NCDs) in the mother and child [8, 11, 13, 18, 29].

> **“...healthy pregnant women can begin or maintain moderate intensity aerobic exercise programs with no risk...”**

# RECOMMENDATIONS FOR PHYSICAL ACTIVITY DURING PREGNANCY

2

Physical Activity and Exercise should be part of an active lifestyle during pregnancy and the puerperium, as shown by growing evidence on its benefits for the health of pregnant women and newborns. Currently there is consensus that maintaining light to moderate physical activity during an uncomplicated pregnancy has several benefits for the health of the woman and the fetus. Pregnancy provides good opportunities for promoting women's health and an active and healthy lifestyle, including proper nutrition and sleep patterns.

As endorsed by the World Health Organization, "some physical activity is better than none". However, appropriate and supervised exercise prescription is needed to tailor effective and safe exercise programs. The fact that the World Health Organization provides specific recommendations on physical activity for pregnant and postpartum women since 2020 [1], highlights the relevance of this topic.

The dissemination of official guidelines among pregnant women significantly contributes to increased participation in prenatal exercise [30]. Official guidelines published by national and international obstetrics, gynecology, or sports medicine institutions are a trustworthy and comprehensive source of information about safety and health benefits of exercise during pregnancy and should be fostered by health professionals. They should thus be accessible to all interested parties: pregnant women and their families, obstetric care providers, physiotherapists / kinesiologists and exercise professionals to enable an effective cooperation in the exercise program design.

An extensive review of recent guidelines is provided elsewhere [31-34]. The recommendations on physical activity during pregnancy and postpartum, published recently, are summarized in Box 1 [34].

## Box 1 Summary of the recent recommendations on physical activity during pregnancy and postpartum

| |
|---|
| All women without contraindications should be encouraged to participate in aerobic and strength-conditioning exercises as part of a healthy lifestyle during their pregnancy |
| 150 to 300 minutes of intentional physical activity of moderate to vigorous intensity per week |
| The exercise program should include: aerobic exercise and resistance exercise and also pelvic floor muscle training, flexibility, balance, and coordination exercise |
| Time spent on sedentary activities should be limited |
| Recommended activities / exercises (adapted if needed) e.g.: Aerobics, dancing, walking, jogging, running, resistance training, swimming, water exercise, cycling, cross-country skiing, Pilates, yoga, balance and posture, exercises preparing for childbirth |
| Activities / exercises recommended to avoid: e.g., Scuba diving, horseback riding, downhill skiing, team sports with a high potential for contact, activities with a high potential for falls and trauma |
| Pregnant women can consult healthcare professionals anytime to know whether or how to adjust their physical activity during pregnancy and the post-partum period |
| If complications occur, a specialist should be consulted to individualize physical activity, rather than abandoning it |
| Women who habitually engaged in aerobic and/or vigorous-intensity activities before pregnancy can continue these activities during pregnancy and the postpartum period |
| Exercise routines can be gradually resumed after pregnancy depending on the mode of delivery, vaginal or cesarean, and the presence or absence of medical or surgical complications |
| Moderate exercise during lactation does not affect the quantity or composition of breast milk and impact infant growth |
| Nursing women should consider feeding their infants before exercising in order to avoid exercise discomfort of engorged breast |
| Recommended types of exercise are specified for the early postpartum period and after full recovery of pelvic and musculoskeletal structures |
| Return to high impact activities including those with high gravitational load on the pelvic floor should occur gradually, and in consideration of individual recovery processes of pelvic floor and abdominal muscles depending on the mode of delivery. |

The recently published official recommendations on physical activity during pregnancy and postpartum can be accessed in the following links shown in Box 2 [34].

## Box 2 Links to recent recommendations on physical activity during pregnancy and postpartum

SMA - Sport Medicine Australia, 2017 [35]: https://sma.org.au/sma-site-content/uploads/2017/08/SMA-Position-Statement-Exercise-Pregnancy.pdf

IOC - International Olympic Committee, 2018 [36, 37]: https://bjsm.bmj.com/content/52/17/1080.long | https://bjsm.bmj.com/content/51/21/1516.long

SOGC - Society of Obstetricians and Gynaecologists of Canada / CSEP - Canadian Society for Exercise Physiology, 2018 [38]: https://bjsm.bmj.com/content/52/21/1339 • U.S. DHHS - U.S. Department of Health and Human Services, 2018 [39]: https://health.gov/sites/default/files/2019-09/Physical_Activity_Guidelines_2nd_edition.pdf

EIM/ACSM - Exercise is Medicine/American College of Sports Medicine, 2019 [40]: https://www.exerciseismedicine.org/assets/page_documents/EIM_Rx%20for%20Health_Pregnancy.pdf • WHO – World Health Organization, 2020 [1]: https://bjsm.bmj.com/content/54/24/1451.long

NHS - National Health Service (United Kingdom), 2020 [41]: https://www.nhs.uk/pregnancy/keeping-well/exercise/

ACOG - American College of Obstetricians and Gynecologists, 2020 [42]: https://www.acog.org/clinical/clinical-guidance/committee-opinion/articles/2020/04/physical-activity-and-exercise-during-pregnancy-and-the-post partum-period

ACSM - American College of Sports Medicine, 2020 [43]: https://www.acsm.org/docs/default-source/files-for-resource-library/pregnancy-physical-activity.pdf?sfvrsn=12a73853_4

RANZCOG – The Royal Australian and New Zealand College of Obstetricians and Gynaecologists, 2020 [44]:

AGDH - Australian Government. Department of Health, 2021 [45]: https://www.health.gov.au/resources/publications/physical-activity-and-exercise-during-pregnancy-guidelines-brochure

Brazilian Society of Cardiology, 2021 [46]: https://www.ncbi.nlm.nih.gov/pmc/articles/PMC8294738/#S01

Australian guidelines for physical activity in pregnancy and postpartum: https://www.jsams.org/article/S1440-2440(22)00068-8/fulltext [131]

# 3 HEALTH SCREENING AND PRE-EXERCISE ASSESSMENT

Health screening, i.e., the review of overall health and medical and obstetric risks by a healthcare professional should precede an exercise program during pregnancy [38, 42] to confirm the absence of contraindication to physical activity. Commonly observed pregnancy-related symptoms (such as low back pain, tiredness or nauseousness) may interfere with the adoption of an active lifestyle. Information on body changes and health benefits through personalized counselling about physical activity is recommended for each profile of women to overcome barriers.

The ACOG's Antepartum Records and the Postpartum Care forms can assist health care providers. These forms are available at the ACOG website upon registration:

https://www.acog.org/clinical-information/obstetric-patient-record-forms

The GET ACTIVE QUESTIONNAIRE FOR PREGNANCY by CSEP [47] is a 2-page guideline for health screening facilitates the communication between healthcare professionals, exercise specialists and pregnant women. It includes guidance on exercise prescription, healthy lifestyle during pregnancy, and exercise safety. The questionnaire is available at: • https://csep.ca/wp-content/uploads/2021/05/-GAQ_P_English.pdf The companion HEALTH CARE PROVIDER CONSULTATION FORM FOR PRENATAL PHYSICAL ACTIVITY by CSEP [48] helps healthcare professionals to engage in a meaningful conversation about health benefits of physical activity during pregnancy. The questionnaire is available at: • https://c-sep.ca/wp-content/uploads/2021/05/GAQ_P_HCP_English.pdf

Another validated and comprehensive inventory to objectively monitor pregnancy-related symptoms is available from Foxcroft et al. [49]:

https://bmcpregnancychildbirth.biomedcentral.com/articles/10.1186/1471-2393-13-3

These forms may be used in combination with other preliminary screening tools, such as the Physical Activity Readiness Questionnaire for Everyone (PAR--Q+) available at the official website [50]. This questionnaire is available at:

http://eparmedx.com/

## Contraindications for physical activity in pregnancy

Pregnant women and exercise and health professionals should also be familiar with the absolute and relative contraindications to exercise during pregnancy, as well as with signs and symptoms to cease exercise and seek medical attention. The following boxes summarize the factors included in several recommendations (adapted from [34]) . It should be emphasized that the following lists of contraindications to physical activity during pregnancy in many cases is based on the consensus of experts' opinions. Many studies and analyzes are currently being conducted, based on which the presented list may be modified in the future[132].

### Box 3 Signs and symptoms to cease exercise and seek medical attention

| |
|---|
| Feeling faint or headache |
| Chest pain |
| Lower back, pelvic or abdominal pain (potentially indicating pre-term labor) |
| Calf pain or swelling |
| Sudden swelling of the ankles, hands or face |
| Dizziness or presyncope or faintness that does not go away with rest |
| Shortness of breath that does not go away with rest / dyspnea before exertion |
| Unexplained / excessive shortness of breath |
| Excessive fatigue |
| Muscle weakness |
| Regular painful uterine contractions |
| Decreased fetal movement |
| Bleeding or amniotic fluid coming from vaginal (indicating rupture of the membranes) |

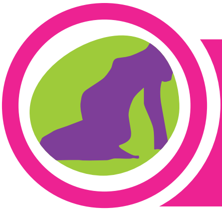

## Box 4 Relative contraindications for exercising during pregnancy

| |
|---|
| History of fetal growth restriction |
| Miscarriage / recurrent pregnancy loss |
| History of premature birth or labor |
| Cervical enlargement |
| Persistent bleeding in the second or third trimesters |
| Symptomatic anemia |
| Poorly controlled seizure disorder |
| Multiple gestation (triplets or higher) after the 28th week |
| Unevaluated maternal cardiac arrhythmia |
| Gestational hypertension |
| Mild/moderate cardiovascular disease |
| Chronic bronchitis or other respiratory disorders |
| Poorly controlled type I diabetes |
| Extreme underweight |
| Malnutrition, eating disorder |
| Orthopedic limitations |
| Other significant medical conditions |

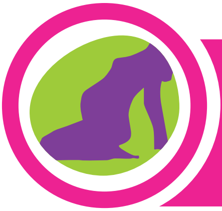

## Box 5 Absolute contraindications for exercising during pregnancy

| |
|---|
| Hemodynamically significant heart disease / other serious cardiovascular disorder |
| Incompetent cervix / cerclage |
| Intrauterine growth restriction in current pregnancy |
| Multiple gestation at risk of premature labor |
| Other serious systemic disorders |
| Placenta previa after 26/28 weeks' gestation |
| Pre-eclampsia / pregnancy-induced hypertension |
| Premature labor during the current pregnancy |
| Restrictive lung disease / respiratory disorder |
| Ruptured membranes |
| Severe anemia |
| Uncontrolled hypertension |
| Uncontrolled thyroid disease |
| Uncontrolled type I diabetes |
| Unexplained persistent vaginal bleeding / persistent second or third trimester bleeding |

Any of these signs or symptoms should induce participants to cease or limit physical activity to avoid the risk of future complications. In this case, it is of utmost importance to interact with the exercise professional to adapt daily routine and/or exercise program.

## Evaluation of physical activity level and setting of objectives

A healthy pregnant woman without complications may decide either to engage in a supervised or unsupervised physical activity / exercise program that is – ideally – adapted (e.g., through realistic objectives) and motivating (e.g., objecti

ve measures of volume). No tests or monitoring are needed. However, wearable technology (i.e., pedometers, accelerometers in combination with smartphone apps) and questionnaires are basic and affordable equipment to monitor physical activity.

Pedometers allow for step-count only and are less accurate and reliable than accelerometers [51]. They provide an inexpensive tool to objectively measure walking and are integrated in smartphones.

Questionnaires can be used to recall physical activity pattern and volume, complementing the information provided by wearables. The PPAQ - Pregnancy Physical Activity Questionnaire [52], is a widely used tool for the assessment and measurement of physical activity levels amongst pregnant women. PPAQ is a self-administered questionnaire which assesses sedentary, light, moderate, and vigorous activities regarding household/caregiving, occupational, and sports/exercise activities respectively. Pregnant women are asked to select the category that best reflect the amount of time spent in 32 activities and in inactivity for their current trimester of pregnancy. At the end of the questionnaire, an open-ended section allows to add activities that are not listed. The PPAQ is available at:

https://journals.lww.com/acsm-msse/Fulltext/2004/10000/Development_and_Validation_of_a_Pregnancy_Physical.14.aspx

Pregnancy is a complex phenomenon that considerably differs from one woman to another, and even for one and the same woman having several pregnancies. Thus, the exercise specialist may also raise other questions regarding the stage of pregnancy, objectives, motivations, potential barriers, and facilitators to adopt an active and healthy lifestyle. This information is needed in order to develop a tailored exercise prescription plan. With this purpose in mind, the exercise specialist may raise the following questions:

Is this your first pregnancy?

How did previous pregnancies go?

How old are you?

What is the current stage of pregnancy?

What are the main pregnancy-related symptoms?

What is your self-perception of health?

What are the main characteristics of your lifestyle, regarding nutrition, sleep, stress, physical activity?

Are you aware of the benefits of exercising?

Would you like me to introduce you to the physical activity recommendations and the associated health benefits for you and your baby?

Are you aware of relative and absolute contraindications to exercising?

What are your occupational and other leisure activities?

What are the main characteristics of your physical activity level and pattern?

When to start exercise during pregnancy / postpartum?

Would you like to adapt your amount of physical activity now that you are pregnant / have given birth?

What have you done before?

Why do you want to change?

What would be the advantages/disadvantages of doing more physical activity?

What are your main objectives and motivations for physical activity?

What are the barriers to increase/maintain your physical activity level?

Which activities would you like to do?

In summary, three profiles of pregnant women can be distinguished in terms of exercise practice and should be advised differently:

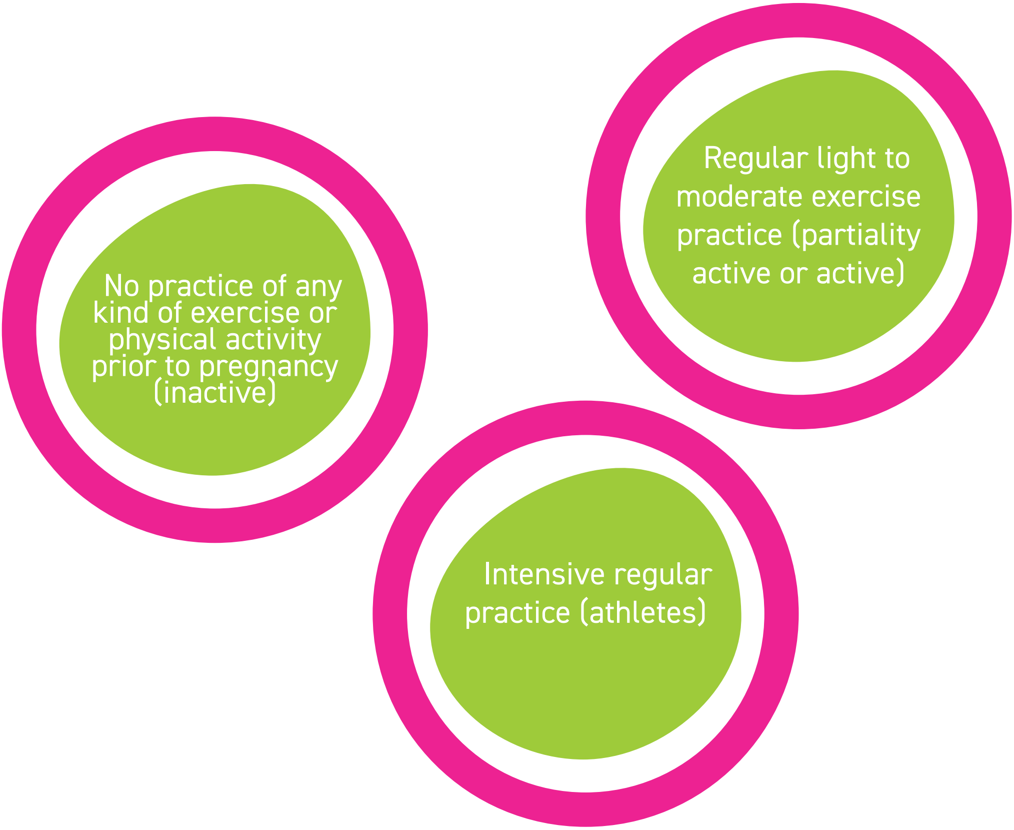


Current research shows that healthy pregnant women can engage in moderate physical activity and maintain moderate to vigorous exercise programs respectively with no risk of adverse effects on their unborn fetus [38, 40, 46]. The recommended volume of physical activity may be achieved by engaging in a formal exercise program or by increasing daily physical activity (box 6), independently of the previous level.

## Box 6 Forms of increasing the volume of physical activity or exercise through occupational activities, active commuting, and daily activities (adapted from [53])

| |
|---|
| Starting the day with some stretching |
| Increasing walking time (i.e. walking the dog, parking further away from the office or residence) |
| Using a pedometer and aiming for 10,000 steps per day |
| Walking or biking for active commuting |
| Engaging in walking groups with other mothers and babies, or with family and friends |
| Taking stairs instead of the elevator as often as possible |
| Doing housing chores, gardening, etc. |
| Avoiding standing in the same position for long periods of time |
| Limiting the seating time (i.e., using a standing desk) |
| Doing stretching breaks during seated activities or desk exercises, dancing with music at home |
| Reducing sedentary activities (e.g., television watching, computer use, sitting in a car or at a desk) |
| Getting all family involved (i.e., playing with the baby or other children) |
| Using available mobile applications that encourage physical activity |
| Performing short bouts of exercise at home (e.g., by following YouTube videos [69]), such as: Active Pregnancy YouTube Channel: https://www.youtube.com/channel/UC0-VyookwcOmcQ5T70imtoNA/playlists |

## Previously inactive women

Previously inactive women can engage in light activity anytime and progressively increase. Women who identify as inactive before pregnancy usually become more sedentary and less physically active as pregnancy progresses. So, providing women with adequate information could support them in planning and starting some exercise program. Doing any physical activity is better than doing none [1]. Thus, previously inactive women should start with small amounts of physical activity and gradually increase frequency, intensity and duration over time.

## Partially active and active women

Previously active women can continue their daily physical activity and exercise routine. Women who were partially active/active before pregnancy and became less active or even inactive during pregnancy are also unaware of the benefits of physical activity during pregnancy and postpartum. More concrete information on "what", "how" and "where" could support them to achieve the recommendations.

## Very active women and athletes

Very active women and pregnant athletes can also continue their daily physical activity and exercise routine with some adaptations regarding exercise selection and safety. Regarding the pregnant athlete, vigorous intensity exercise appears to be safe in healthy pregnancies [42] unless extreme sports are practiced [54]. Elite athletes, who wish to become pregnant, should discuss specific issues with their medical team [38, 55]. According to the Australian Sports Commission [54], recreational and competitive athletes may train safely at high intensities and volumes throughout pregnancy while being under obstetric supervision. Due to the risk of trauma, athletes performing contact sports may be advised to switch to fitness-oriented exercises. The key message of the International Olympic Committee (IOC) [36] is that elite athletes with an uncomplicated pregnancy should be reassured that they can continue exercising, although some adjustments in intensity and activity may be required.

# BENEFITS OF PHYSICAL ACTIVITY DURING PREGNANCY

# 4

Extensive exercise testing during pregnancy is usually only performed for medical reasons or for research purposes [5] unless desired by the pregnant woman. Special care should be taken to ensure that the pregnant woman feels comfortable and there are no risk of falls and injuries. Exercise testing should not be invasive during pregnancy. Instead, it should increase motivation, objectively evaluate the effects of training, and support exercise prescription through the assessment of baseline fitness level, as according to good practice based on the RANZCOG guidelines [44]. In a clinical and research setting, cardiopulmonary exercise testing during pregnancy is valuable in identifying underlying cardiopulmonary conditions, stratifying the risk of adverse pregnancy outcomes, as well as establishing exercise tolerance/limitations [57].

According to the ACSM [5], maximal exercise testing should not be performed unless medically necessary, and under medical supervision. There are several submaximal tests to evaluate physical fitness components (i.e., cardiorespiratory endurance, body composition, muscular strength and endurance, and flexibility), and skill-related components of physical fitness (i.e., agility, coordination, balance, power, reaction time, and speed) [4, 5]. Most tests have been developed for a healthy adult population and allow for an objective evaluation of fitness status and effectiveness of an exercise intervention.

Mottola et al. [58] developed and validated heart rate (HR) ranges that correspond to moderate intensity exercise for low-risk pregnant women based on age and body mass index (BMI) and fitness levels. Those HR ranges are provided in the Canadian [38] and RANZCOG [44] guidelines.

Regarding maximal heart rate, the estimation equations by Gellish et al. [59] are more accurate than other formulas, and were estimated for men and women participants in an adult fitness program with a broad range of age and fitness levels, as follows:

(eq.1) $$HRmax = 207 - (0.7 \times age)$$

(eq.2) $$HRmax = 192 – (0.007 \times age^2)$$

Although the nonlinear predictor model (eq. 2) was slightly more accurate than the linear equation (eq. 1), the authors suggest that the linear model is easier to use.

After estimating HRmax and monitoring heart rate at rest, the exercise intensity can be estimated using the heart rate reserve (HRR) equation [4, 5]:

(eq.3) HRR method (training) = [(HRmax – HRrest) x intensity in percent] + HRrest (bpm)

Submaximal exercise testing is more appropriate for pregnant women [5] in order to estimate the maximum rate of oxygen utilization of muscles during exercise (VO2max). Treadmill walking and upright leg cycling are the most common and convenient testing modalities during pregnancy, since the injury risk is low, physiological monitoring easy (not much vertical movement), and exercises based on basic movements. Another exercise testing option is a field test consisting of walking over a predetermined time or distance, such as the 6-min walk test. Those tests are easy to administer and require little equipment. The 6-min walk test (6MWT) assesses the distance for a 6 min time interval [5]. This test is easy and safe to administer, requires little equipment and there are existing references for resting HR and distance walked [60].

There are no specific tests for the assessment of musculoskeletal function, i.e., muscular strength and resistance, and flexibility, although these components of physical fitness are addressed in the recommended guidelines for exercise during pregnancy [4, 53]. Further details on musculoskeletal health adaptations during pregnancy can be found in Fitzgerald and Segal [61].

The IFIS - International FItness Scale for self-reported fitness is a simple-to-use tool with demonstrated validity and reliability [62]. The IFIS might be a useful tool for identifying pregnant women with low or very low physical fitness and with low health-related quality of life [63]. It also can be useful to stratify pregnant women in appropriate fitness levels on a population-based level where objective measurement is not possible [64]. This scale is available at:

http://www.helenastudy.com/ifis.php

Body composition is also a health-related component of physical fitness. Monitoring weight gain and nutritional status is insightful during pregnancy, especially for women with excessive weight or obesity status. In these cases, the assessment of body composition, body circumferences, body fat distribution markers and other body indexes can be used [65]. Excessive weight gain is undesirable; however, guilt or blame in pregnant women with regarding their eating habits need to be avoided. Dietary analysis and tailored nutrition plans should be conducted by a qualified nutritionist. Further development on this topic can be found elsewhere [66, 67].

**The IFIS might be a useful tool for identifying pregnant women with low or very low physical fitness and with low health-related quality of life**

# 5 EXERCISE PRESCRIPTION FOR PREGNANT WOMEN

Regarding exercise prescription and monitoring, pregnant women fall into the category of apparently healthy adults, although they are considered a special population [5]. Thus, the general guidelines of ACSM regarding the "FITT-VP principle" apply to pregnant women, with some modifications [5, 53]. This principle is based on the following elements that address one or more physical fitness components:

F – Frequency
(how many exercise sessions per week?)

I – Intensity
(how hard or difficult is the exercise?)

T - Time
(how long is each exercise session?)

T - Type of exercise
(which mode of exercise?)

V – Volume
(which amount?)[10]

P – Progression
(how to advance?)[11]

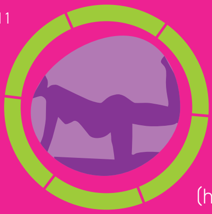

Box 7 resumes the exercise prescription components applied to pregnant women, adapted from Santos-Rocha et al. [53].

[10] Usually, the volume is considered to be the product of intensity, frequency and duration of the exercise sessions.

[11] With pregnant clients, the "progression" is assumed as the adaptation of exercise to each trimester of pregnancy, rather than focused on intensity and complexity, taking into account the physiological adaptations to pregnancy.

## Box 7 Summary of Exercise Prescription components for pregnant women

| Aerobic Exercise | | | | |
|---|---|---|---|---|
| Type | Intensity | Duration | Frequency | Progression/ Adaptation |
| Exercises that activate large muscle groups in a rhythmic and continuous fashion<br>A variety of weight- and non-weight-bearing activities are well tolerated during pregnancy<br>Aerobic exercises can be categorized by intensity and skill demands | Moderate intensity exercise<br>(3-5.9 METs; RPE = 12-13; 40%-60% VO2reserve)<br>Vigorous intensity exercise<br>(>6 METs; RPE = 14-17) for women who were highly active prior to pregnancy or for those who progress to higher fitness levels during pregnancy<br>So far, there is little evidence on the influence of exercise of high intensity (RPE > 17) on the course of pregnancy. | 30 min / day of accumulated moderate intensity exercise to total at least 150 min / week<br>or<br>75 min / week of vigorous intensity<br>or<br>a mix between moderate and vigorous intensity<br>Previously inactive women should progress from 15 to 30 min / day | Previous sedentary: up to 3 days / week<br>Previous active: 3-5 days / week to most days of the week | Avoid activities with risk of fall and trauma<br>Activities that require jumping movements and quick changes in direction which can stress joints should be done with caution to –minimize the risk of joint injury |

| Resistance Exercise | | | | |
|---|---|---|---|---|
| Type | Intensity | Duration | Frequency | Progression/ Adaptation |
| A variety of machines, free weights, and body weight exercises are well tolerated during pregnancy | Intensity that permits multiple submaximal repetitions (i.e., 8-10 or 12-15 repetitions) to be performed to the point of moderate fatigue<br>(40%-60% of estimated one repetition maximum) | 1-2 sets for beginners<br>2-3 sets for intermediate and advanced<br>Target major muscles groups<br>A basic program includes 8-10 different exercises | 2-3 nonconsecu-tive days / week | If causing discomfort, modifying the supine position of the exercise to instead be performed on one's side, sitting or standing is a safe alternative<br>Avoid perfor-ming the Valsalva maneuver<br>during exercise<br>Heavy-resistan-ce weight lifting and intense repetitive isometric exercises should be performed with caution until more data is available |

| Flexibility Exercise | | | | |
|---|---|---|---|---|
| Type | Intensity | Duration | Frequency | Progression/ Adaptation |
| A series of active or passive static and dynamic flexibility exercises for each muscle--tendon unit | Stretch to the point of feeling tightness or slight discom-fort | Hold static stretch for<br>10-30 s<br>(up to 60 s)<br>2-4 repetitions of each exercise | At least 2-3 up to 7 days / weekk | Avoid excessive joint stress |

| Neuromotor Exercise | | | | |
|---|---|---|---|---|
| Type | Intensity | Duration | Frequency | Progression/ Adaptation |
| Exercises involving motor skill, e.g., balance, agility, coordination, gait), proprio-ceptive training, and multifaceted activities (e.g., Pilates, Yoga, tai chi) | Intensity in balance training refers to the degree of difficulty of the postures, movements, or routines practiced<br>An effective intensity (and volume) of neuromotor exercise has not been determi-ned | 20-30 to 60 min / day | At least 1-2 up to 7 days / week Can be included in daily activities | Avoid positions that are uncomfortable or likely to result in loss of balance and falling |

| Pelvic floor training | | | | |
|---|---|---|---|---|
| Type | Intensity | Duration | Frequency | Progression/ Adaptation |
| Complex training for pelvic-floor muscles should be focused both on their contraction and relaxation<br>Various devices can be used to increase the effectiveness and attractive-ness of exercise (e.g. vaginal cones) | An effective intensity (and volume) of pelvic floor exercise has not been determi-ned | 10-20 min / day | 1-7 days / week Should be incorporated in any prenatal exercise program | Can be done anywhere, anytime, everyday Ensure proper technique: on the expiration contraction of perineal muscles and then transversal muscles Different exercises should be performed to improve pelvic floor muscle speed, strength, endurance and muscular coordination, and engaging both fast and slow twitch muscle fibers |

## Types of physical exercises

Plenty of physical activities can be performed alone or in group, indoor or outdoor, with or without equipment. Among the recommended are: walking, jogging, indoor cycling, cross-country skiing, swimming, water exercise, low-impact aerobics, step, dancing, Pilates, yoga, flexibility, balance, posture and functional, pelvic floor muscles training, and resistance training [34]. Other safe activities (for pregnant women who participated in these activities regularly before pregnancy) are running, outdoor cycling, strength training and racquet sports, upon consultation with an obstetric care provider [42, 44]. Further explanation of each type of exercise can be found in Szumilewicz and Santos-Rocha [68]. Further examples of exercises and sessions tailored to pregnant and postpartum women can be found in the ACTIVE PREGNANCY YouTube channel [69]:

https://www.youtube.com/channel/UC0Vyookwc0mcQ5T70imtoNA/playlists

A comprehensive exercise program during pregnancy may include several of the recommended types of exercise and should address all physical fitness components (i.e., aerobic, resistance training, and flexibility), as well as neuromotor exercises, pelvic floor training, and preparation for birth exercises [53, 70]. Each exercise session could consist of at least 30 minutes of aerobic exercise, strength and flexibility training (including posture and functional exercise), neuromotor exercise (especially, balance and coordination), and pelvic floor muscle training. For example, a step exercise session may combine aerobic, lower limb resistance, and neuromotor training, while a Pilates exercise session may combine upper, core and lower limb resistance, posture, flexibility, and neuromotor training.

A typical exercise session should be organized as follows [5, 53]:

- 5-10 min warm-up period, including, walking, pelvic floor training, light stretching, or movements performed during the main part of the session. Some fun exercises in groups could be done to promotion social inclusion.
- the main part should include endurance, strengthening and balance exercises. Each exercise should be presented with variations to let the women choose the best fit.
- cool-down period including breathing exercises, light stretching, mobility exercises, pelvic floor training.

Regarding exercise selection and adaptation, exercise professionals must be aware of the morphological [65], physiological [71], musculoskeletal [61, 72] and biomechanical [73] changes that occur during pregnancy, such as increased ligament laxity, weight gain, increased fatigue, change in the center of gravity, carpal tunnel syndrome and vena cava syndrome that will affect the response to exercise. Moreover, typical symptoms associated to each trimester of pregnancy, motivations and objectives, safety considerations, fitness level, and level of experience of the pregnant women will also impact on exercise selection and adaptation [53, 68]. For instance, indoor or outdoor cycling, aerobic and step exercise can be performed at different intensity and complexity levels. For a pregnant woman who has never practiced these activities, the entire learning process must be kept in mind so as not to jeopardize her safety in the event of falls or collisions. Several guidelines [35, 38-40, 42, 45] also list non recommended physical activities, including contact sports, activities with high risk of falling, activities at high altitude (when not normally living at high altitude), scuba diving, skydiving, downhill skiing, water skiing, activities in excessive heat (e.g., hot Pilates, and hot yoga). Further explanation can be found in Szumilewicz et al. [34].

## Exercise duration, frequency, and intensity (volume)

Exercise duration is prescribed as the amount of time physical activity is performed, i.e., time per session, per day, and per week [5]. Frequency is prescribed as sessions per day and as days per week. Exercise duration typically ranges from 20 to 60 min. Most guidelines for exercise during pregnancy [34] suggest 30 minutes of daily exercise, five to seven days per week. Previously inactive or not regularly exercising women should begin with 10 to 20 minutes of continuous low-intensity exercise three times per week, gradually increasing the intensity, frequency, and duration. If an inactive woman would like to participate in a one-hour group session, the healthcare professional should encourage her.

Exercise professionals can empower women using two practical methods to monitor intensity in practice [42]:

Perceived exertion (overall sensation of effort): for moderate exercise, ratings of perceived exertion should be 13 to 14 (somewhat hard) on a Borg Rating of Perceived Exertion scale, where 6 represents no exertion and 20 represents maximal exertion.

The "talk test": the individual should be able to speak comfortably and in complete sentences reflecting moderate exercise intensity. In contrast, vigorous exercise is associated with substantial increases in breathing, thus an inability to carry on a conversation easily, and perspiration.

Exercise volume is the resultant of frequency, intensity, and exercise duration. Usually, the exercise volume is used to estimate the gross energy expenditure in metabolic equivalents (in MET-min/week or in kcal/week) with respect to body composition and weight management outcomes [4, 5]. Another form of estimating the exercise volume is via steps per day using pedometers. The goal of 10,000 steps / day is often cited regarding health benefits, but a pedometer step count of at least 5,400–7,900 steps / day can already meet recommended exercise targets. This step count volume is approximately equal to 1,000 kcal / week or 150 min / week of moderate-intensity physical activity [5].

## Exercise progression and adaptation to body changes

Exercise progression may vary at different time points during pregnancy, thus exercise routines should remain flexible and in accordance with the physiological and biomechanical adaptations occurring over the time course of pregnancy [53, 68, 74].

## First trimester

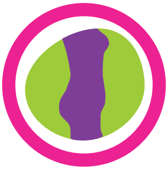

The right amount of exercise for a pregnant woman will depend on how active she was before pregnancy. Most women don't need to modify their activities too much during the first trimester of pregnancy. Usually, it is best to favor low impact exercises, e.g., walking, Nordic walking, low-impact aerobics and step, yoga, Pilates, resistance training, indoor cycling, swimming, and water aerobics. Slightly more vigorous exercises may also be appropriate in the first trimester, in case the woman is used to them, e.g., running, jogging, cross-country skiing, outdoor cycling, and moderate weightlifting, etc. Pelvic floor training should be advised from the first trimester. In the first trimester, most pregnant women experience symptoms, such as mood changes, nausea, vomiting, breast tenderness, dyspepsia, frequent urination, and constipation, which can limit daily and physical activities. Symptoms and discomfort may prevent some women from practicing physical activity or exercise, and may require adaptations of the types of exercise selected.

## Second trimester

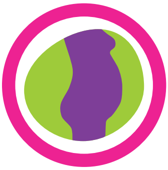

During the second trimester, discomfort of the first trimester is usually gone and physical limitations of the third trimester have not yet appeared. At this stage, the uterine volume increases and the inferior vena cava syndrome can develop. This results in a reduction of venous return through said vessel, due to the pressure exerted by the pregnant uterus in supine position. Some women may therefore feel discomfort or dizziness while lying on their back. In practice, many women report sleeping while lying on back. Nevertheless, some guidelines recommend avoiding this position after the first trimester of pregnancy, in case of discomfort [40, 44].

## Third trimester

During the third trimester, the increased volume of the gravid uterus as well as the weight gain of the pregnant woman compromises the volume at the abdominal and pulmonary level. Consequently, pregnant women tend to decrease the intensity and duration of their physical activity. It is therefore recommended to start or maintain an adequate amount of physical activity particularly in the third trimester. Activities in the aquatic environment are adequate (weightlessness, mobilizing joints with passive resistance, the appearance of inferior vena cava syndrome is attenuated). The AGDH guidelines [45] recommend that after 28 weeks, exercises should not be performed lying flat on back, but in an upper body tilt of 45-degree angle or while lying on the side. Moreover, decreased balance and coordination can lead to falls. Balance and coordination exercises are recommended at each stage of pregnancy. Hormonal and biomechanical adaptations may be associated with joint and low back pain that could be minimized by strengthening abdominal and back muscles [42]. Pelvic floor muscles training should be maintained. Walking or Nordic walking with good posture and at varying paces can be performed autonomously any time. During the third trimester, women should be advised to prepare for birth program [75-77].

**Exercise progression may vary at different time points during pregnancy**

# EXERCISE PRESCRIPTION IN SPECIAL CONDITIONS

# 6

Exercise can be a recreational or competitive component but also serve as an adjunct treatment for several disorders, such as gestational diabetes, excessive weight gain and obesity, low back pain, and antenatal depression and prevention of hypertension and preeclampsia [10, 13, 78]. Women suffering from these conditions face substantial barriers to participate in exercise and require support to initiate and adhere to physical activity. Under medical supervision and in inter-professional collaboration, exercise professionals may need to select appropriate exercise interventions.

## Gestational diabetes

A healthy pregnancy can be associated with resistance to insulin on glucose uptake and utilization [79]. In 1-14% of pregnant women this condition develops into gestational diabetes mellitus (GDM) [80]. Gestational diabetes is the most common metabolic disorder in pregnancy and its prevalence is nowadays increasing because there is a higher number of pregnant women with a body mass index (BMI) or weight gain level in the range of overweight or obesity, and also because childbearing age is increasing [80]. GDM is associated with a wide range of adverse health consequences for women and their infants in the short and long term, including an increased risk of macrosomia, birth complications, and maternal diabetes after pregnancy. It may also increase the risk of obesity and type 2 diabetes in offspring later in life [81, 82]. There is growing evidence that exercise and physical activity can control this condition while being easy to be carried out, effective and with minimum costs [83].

Observational studies strongly support exercise and physical activity as a tool that may control glycemia levels in pregnancy and reduce the risk of gestational diabetes. In the last few years, various studies showed the following protective functions of exercise and physical activity:

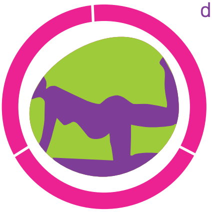


To achieve at least a 25% reduction in the odds of developing GDM (and also gestational hypertension and pre-eclampsia), pregnant women need to accumulate at least 600 MET-min/week of moderate-intensity exercise (i.e., approximately 140 min) [15].

Exercise prescription is the same for pregnant women with and without gestational diabetes. However, pregnant women who need glucose-lowering drugs for metabolic control of glycemia should be closely monitored, since exercise may misadjust the prescribed pharmacological regimen.

## Excess weight and obesity

Gestational weight gain (GWG) has often been identified as critical for maternal and fetal health [87]. The ACOG states that "in pregnancy, physical inactivity and excessive weight gain have been recognized as independent risk factors for maternal obesity and related pregnancy complications, including gestational diabetes mellitus" [88]. The ACOG advice that obese pregnant women should be encouraged to engage in a healthy lifestyle including physical activity and judicious diets [88]. This special population should start with low-intensity, short periods of exercise and gradual increase of exercise volume [38, 42]. In the last few years various studies showed:

Supervised physical activity combined with diet programs were most effective in managing weight among overweight and obese pregnant and postpartum women [89], lower the odds of a caesarean section [90] and prevent postpartum weight retention

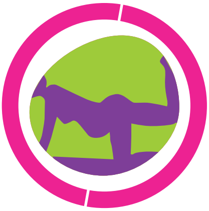

Exercise in obese pregnant women reduces the odds of excessive weight gain [87,89, 90]

## Hypertension and preeclampsia

Hypertension12 in pregnancy may result in adverse perinatal outcomes for mother and fetus / newborn. Preeclampsia in pregnancy and postpartum is due to an abnormal development of uterine spiral arteries. Despite scientific advances in the understanding of risk factors for preeclampsia, and preventive measures, the condition remains the second most prevalent cause of global maternal mortality, reaching 14% [91].

At present, most research in preeclampsia focuses on improving the development of uterine spiral arteries, determining factors related to genetic predisposition, and improving the low immunological response of most pregnant women that develop this pathology [92]. Exercise plays a key role in view of increasing placental vascularity and stimulating the immune system [93]. In the last few years various studies showed:

Exercise is safe and beneficial in pregnancies involving hypertension (therapy) [14, 15, 94, 95]

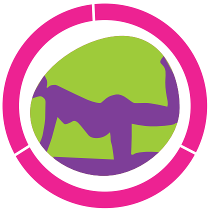

Aerobic exercise for about 30-60 min, two to seven times per week during pregnancy, is associated with a significantly reduced risk of gestational hypertension as compared to sedentary behavior. However, the recommended exercise intensity for these conditions remains unclear [14]

To achieve at least a 25% reduction in the odds of developing gestational hypertension and pre-eclampsia (and also GDM), pregnant women need to accumulate at least 600 MET-min/week of moderate-intensity exercise (i.e., approximately 140 min) [15]

## Low back pain

The majority of pregnant women experience low back pain that interferes with daily routine and worsens as pregnancy progresses. In the last few years various studies showed:

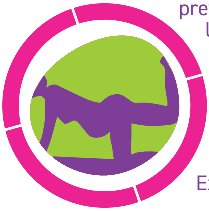

The effectiveness of exercise in reducing back pain and preventing lumbopelvic pain in pregnancy [22-24, 78, 96]

Exercise reduced the risk of low back pain in pregnancy by 9% and prevented sick leave due to lumbopelvic pain [97]

Prenatal exercise decreased the severity of low back, pelvic girdle, and lumbopelvic pain during and following pregnancy [22]

Exercise appears to reduce the risk of low back pain in pregnant women, but there is no clear evidence for an effect on pelvic girdle pain [23]

However, in the listed studies, exercise interventions varied in type, frequency, intensity and duration, hindering a possible association of specific types of exercise interventions with these outcomes [78].

## Depression and mental disorders

The prevalence of depression during the first trimester of pregnancy was reported as 7.4-11%; in the second trimester 12.8%; and in the third trimester 8.5-12%. The prevalence at 40 weeks of pregnancy was 18.4% [98, 99]. Practice of physical activity and exercise is considered as a therapeutic complement to pharmacological treatment even in major depression cases [100]. Various studies showed that:

[12] The ACSM [5] defines ‘hypertension’ as: having a resting systolic blood pressure (BP) of 130 mmHg or greater; having a resting diastolic BP of 80 mmHg or greater; taking antihypertensive medication; being told by a physician or health professional on at least two occasions that one has high BP; or any

Light-to-moderate aerobic exercise improves mild-to-moderate depressive symptoms, and increases the likelihood that mild-to-moderate depression will resolve in the postpartum period [27, 28]

Exercise in pregnancy may prevent perinatal depression and anxiety [78]

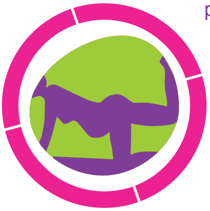

Prenatal exercise reduced the odds and severity of prenatal depression [25, 26]

Sufficient physical activity was associated with a reduced likelihood of probable antenatal depression and trait anxiety symptoms [101]

## Urinary incontinence

Pelvic floor muscle dysfunctions can lead to urinary incontinence, a condition which often affects women during pregnancy and postpartum. Urinary incontinence (UI) is prevalent in antenatal and postnatal women, and pelvic floor muscle training (PFMT) is the first-line treatment for UI [20, 102, 103]. In case of symptoms, assessment and training by a gynecologist/midwife and women's health physiotherapist is indicated [36]. According to AGDH guidelines [45], pelvic floor exercises strengthen and tone the pelvic floor muscles and other tissues. Moreover, a strong pelvic floor can reduce the chance of complications (such as UI) after giving birth and later in life. Current evidence supports that:

PFMT (e.g., Kegel exercises) may be performed daily to strengthen the pelvic floor muscles and to reduce the odds of urinary incontinence [1, 38, 40, 43, 44]

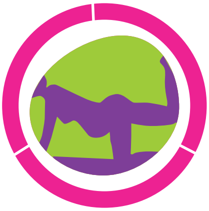

Structured pelvic floor muscle training in early pregnancy for continent women may prevent the onset of UI in late pregnancy and postpartum [20]

According to RANZCOG guidelines [44], activities that involve jumping or bouncing may add extra load to the pelvic floor muscles and should be avoided. However, there is some evidence that women participating in high-low impact exercise program (containing jumps and runs) combined with pelvic floor muscle training, maintained urinary continence, and improved neuromuscular activity of pelvic floor muscles [104]

# 7 SAFETY ISSUES REGARDING EXERCISE DURING PREGNANCY

The following considerations ensure the safety of an exercise program for pregnant women:

## Falls and injury

| |
|---|
| Pregnant women should avoid contact sports and sports or activities that may cause loss of balance or trauma to the mother and fetus (e.g., soccer, basketball, ice hockey, rollerblading, horseback riding, skiing, snowboarding, scuba diving, and vigorous intensity racquet sports) [36, 38]. However, in the absence of medical contraindications, the decision to stop or continue particular disciplines should be based on the assessment of women's individual abilities, skills, previous experience and their sense of security and comfort. |
| Increased body weight is associated with increased loading at the joints [44, 72]. Thus, weight-supported activities such as water-based exercise or stationary cycling may be more comfortable compared with weight-bearing exercises such as walking or jogging in the later stages of pregnancy [44]. |
| During pregnancy, an increase in the laxity of the musculoskeletal system is a natural adaptive process. There is a significant increase in joint laxity in five of seven peripheral joints over the course of the pregnancy and postpartum [105]. |
| An exercise program employing minimal to moderate weight-bearing did not result in any measurable increases in knee laxity and, therefore, appears to be appropriate about knee stability [106]. |
| According to the RANZOG guidelines [45], the increase in ligament laxity associated with pregnancy may have implications for the injury risk. However, no scientific evidence proves a prevalence of joint injury related to physical activity in pregnant women. Activities that require jumping movements and quick changes in direction (e.g., court sports, aerobic dancing, etc.) should therefore be performed with caution [45]. |
| According to the SMA guidelines [35], stretching exercises are useful but should be done gently due to the increased joint laxity during pregnancy. Because of increased relaxation of ligaments in pregnancy, joints are supported less effectively, especially in women with poor muscle mass. Activities that may result in excessive joint stress should be discontinued or adapted. |

The altered center of gravity resulting from the change in weight distribution as pregnancy progresses may influence balance [44, 72]. Thus, precaution should be taken to modify the exercise routine to minimize or avoid fast changes in direction, if necessary [44].

Balance exercises can improve the ability to resist forces within or outside of the body that cause falls while a person is stationary or moving. Strengthening muscles of the back, abdomen, and legs also improves balance [39].

When jogging, running or cycling, rocky terrains or unstable grounds should be avoided, since the joints are more lax in pregnancy, and ankle sprains and other injuries may occur.

## Nausea and dizziness

For all activity types, the Valsalva maneuver, prolonged isometric contractions, and prolonged (motionless) standing should be avoided [5]

High-intensity or prolonged exercise over 45 minutes can lead to hypoglycemia; therefore, adequate energy intake before exercise, or limiting the exercise session, is essential to minimize this risk [42]

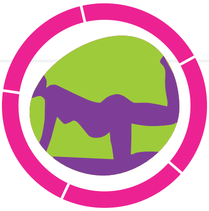

Some women will need to avoid physical activity in the supine position notably after week 16 of pregnancy [38, 42]. Due to the weight of the growing fetus, exertion or prolonged periods in the supine position may reduce venous return and cardiac output [5]

Exercise should always be completed with a cool-down and never stopped suddenly [44]

Fast movement changes in the vertical plane (e.g., from lying or sitting to standing; fast stand to sit and sit to stand) are associated with a reduction in blood pressure and may cause dizziness and imbalance.

## Heat, humidity, and environment

Hot environmental conditions and associated heat stress can increase adverse pregnancy outcomes and negatively affect mental health [107].

Evidence suggests that during exercise, evaporative (sweating) and dry (skin blood flow and temperature) heat loss responses increase from early to late pregnancy in addition to greater cardiac output, blood volume and reduced vascular resistance [108].

Pregnant women should avoid exercising in a hot humid environment, always be well hydrated, and dress appropriately to avoid heat stress [38, 42, 45].

Prolonged exercise should be performed in a thermoneutral environment or in controlled environmental conditions (air conditioning) with close attention paid to proper hydration and caloric intake [38, 42].

Pregnant women are sensitive to smell. Exercise should be performed in a clean environment and avoid air pollution.

Women should choose a place with adequate space and floor surface, ventilation, and temperature, as well as proper exercise equipment or common household materials. The key-point is safety, i.e., if there is no bench step, it is preferably to perform the step exercise on the floor (i.e., "invisible step") rather than use an inadequate bench. Another example is to use a stable and not slippy chair, if the exercise program is performed on a chair (e.g., exercises in [69]).

In general, exercising outdoors - and in group - is preferable. Caution should be taken regarding the wind, the rain and slipping pavement while running or cycling. Proper sportswear and shoes, as well as adequate sun and head protection (e.g., hat or bike helmet) are other recommendations for exercising outdoor [68].

## Sportswear and shoes

Endocrine changes occurring during pregnancy result in increased laxity of the ligaments of the foot, and several gait adaptations may occur [73, 109]

Sportswear should be comfortable, functional and (ideally) seamless due to the increased sensitivity of the skin

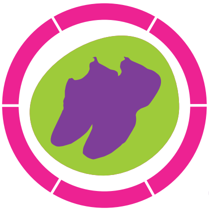

The AGDH guidelines [45] advise that pregnant women should wear appropriate shoes, non-restrictive clothing, and a supportive bra, and - in warm environments - loose clothing made from 'breathable' fabric

Breasts become more sensitive during pregnancy. A supportive sports (or pregnancy specific) bra provides comfort during physical activities, such as, walking and running

Pregnant women should wear comfortable exercise footwear providing ankle and arch support, shock absorption and stability, especially during weight-bearing activities and outdoor activities [73]

Shoe specialists can provide recommendations to meet individual biomechanical profiles

# RECOMMENDATIONS TO REDUCE TIME SPENT IN SEDENTARY BEHAVIOR

# 8

Sedentary behavior increases the risk of cardiovascular and cancer mortality, incident type 2 diabetes, and all-cause mortality [110]. During pregnancy, sedentary behaviors are associated with impaired glucose tolerance, high level of LDL[13] cholesterol and C reactive protein, mental distress, and larger newborn abdominal circumference [111]. Despite those negative consequences, pregnant women spent nearly two-thirds of their day in sedentary behaviors, independently of the trimester [112]. Furthermore, even those women that are compliant with physical activity guidelines at mid-pregnancy, did not reduce the time spent in sedentary behavior [113]. A recent cross-sectional study by Oviedo-Caro et al. [114] estimated a decrease of 4% in cardiorespiratory fitness and 6% in skinfold thickness adiposity when 30 minutes of moderate to vigorous physical activity was replaced by sedentary time.

The WHO states that replacing sedentary time with physical activity of any intensity (including light intensity) provides health benefits [1]. However, there is insufficient evidence to estimate health consequences for different domains of sedentary behavior, i.e., sitting or lying. In addition, more research is needed with regards to health benefits of breaking up prolonged periods of sedentary time [1]. The ACOG's guidelines do not include specific advice on sedentary behavior during pregnancy [42]. The Canadian Society for Exercise Physiology in their 24-hours Movement Guidelines for adults, encourage adults to limit sedentary time to 8 hours or less, by spending no more than 3 hours of recreational screen time and, by breaking up long periods of sitting as often as possible [115]. In addition, CSEP suggests that replacing sedentary behavior by light physical activity; and light physical activity by more moderate to vigorous physical activity - along with sufficient sleep - can provide greater health benefits [115].

[13] Low density lipoprotein cholesterol

In order to obtain an accurate and efficient measure of sedentary time, its definition and differentiation from physical inactivity needs to be understood, i.e., any waking activity involving energy expenditure less than 1.5 METs in a seated, lying, or reclining posture [5]. Devices that combine accelerometer and inclinometer placed on the thigh allow to differentiate between sitting, lying, and standing postures, provide more sensitive measurement of sedentary time than accelerometers only [116].

In addition to objective measures of sedentary behavior, subjective measures are available for pregnant women. Several studies have used the subscale titled sedentary behavior from the Pregnancy Physical Activity Questionnaire (PPAQ) [52], although this subscale has shown poor agreement with devices combining accelerometer and inclinometer [117]. Another tool was designed to evaluate self-reported sedentary behavior in general population, the Sedentary Behavior Questionnaire (SBQ) [118]. This tool has been validated for pregnant women, however, the correlation with combined measures of accelerometer and physiological sensors is weak [119].

**"…CSEP suggests that replacing sedentary behavior by light physical activity; and light physical activity by more moderate to vigorous physical activity …"**

# DIET AND NUTRITIONAL RECOMMENDATIONS DURING PREGNANCY

# 9

"You have to eat this", "you cannot eat that" and the commonplace "you must eat for two" are some of the many advices which any pregnant women will listen to during their pregnancy. Eating healthy and in the right portions for each stage of the pregnancy is the right thing to do. Here's a few simple and practical evidence-based advice on how to eat before and during pregnancy.\

## Before pregnancy

Even before getting pregnant, nutrition is a fundamental aspect in women's health status, therefore is important to assure that no nutritional deficit is present when pregnancy starts.

Two important micronutrients to consider while preparing for pregnancy are iodine and folates [120]. Several countries have guidelines for physicians to supplement women with iodine and folic acid during pregnancy planning. The Recommended Dietary Allowances for folate before pregnancy are 400 µg per day and in Table 1 are some examples of folate rich food sources.

Table 1. Examples of folate food sources (expressed as µg of folate per 100g of edible food).

| Food sources | Mean folate concentration (µg/100 |
|---|---|
| Black-eyed peas, white beans and chickpeas | 370 |
| Liver (beef and pork) | 310 |
| "All-Bran" or "Corn Flakes" cereal | 209 |
| Asparagus, parsley, savoy cabbage, spinach, and watercress | 168 |

The Recommended Dietary Allowances of iodine before pregnancy are 150 µg per day and in Table 2 are some examples of iodine rich food sources, though it is important to highlight that iodine food concentrations are very dependent on soil iodine concentration and therefore highly variable between regions.

Table 2. xamples of iodine food sources (expressed as µg of iodine per 100g of edible food).

| Food sources | Mean iodine concentration (µg/100 g) |
|---|---|
| Codfish | 192 |
| Eggs | 50 |
| Dairy (milk, yogurt, cheese) | 42 |
| Nuts | 31 |

## Weight management during pregnancy

During the first three months of pregnancy, pregnant women have the same energy needs (need the same amount of energy) as before they got pregnant. In fact, just during the second and the third trimester, energy needs increase around 340 and 452 kcal/day, respectively. Weight management during pregnancy is mostly dependent on pregnant women's energy balance, therefore weight variations during pregnancy will depend on women's energy intake and energy expenditure.

Weight increase guidelines during pregnancy are dependent on baseline Body Mass Index (BMI), which is a simple calculation [weight/(height$^2$)] using woman's weight (kg) and height (m) [90]. Table 3 displays the recommended weight increase during pregnancy, considering women's baseline BMI.

Table 3. Recommended weight increase during pregnancy.

| Baseline BMI | Recommended weight increase during pregnancy | Mean weekly weight increase (second and third trimester) |
|---|---|---|
| Underweight < 18.5 kg/m2 | 12.5-18 kg | 0.5 kg |
| Normal weight 18.5-24.9 kg/m2 | 11.5-16 kg | 0.4 kg |
| Overweight 25-29.9 kg/m2 | 7-11.5 kg | 0.3 kg |
| Obese ≥ 30 kg/m2 | 5-9 kg | 0.2 kg |

Twin pregnancies may aim for a total body weight increase between 15.9 to 20.4 kg with a 0.7 kg increasing rate during the second and third trimester.

All previous guidelines are based in the premise that during the first trimester of pregnancy there was no relevant weight increase, and it is also important to highlight that it is normal that the weight increase rate during pregnancy is higher alongside pregnancy.

For example, a woman with 1.64 m and 60 kg will have a BMI of 22.3 kg/m2 [60 ÷ (1.64 x 1.64)], so it would be recommended a total weight gain during pregnancy between 11.5 and 16 kg (a total body weight between 70.5 and 75 kg).

The more the pregnant woman exceeds the recommended weight gain during pregnancy, the greater the risk of the occurrence of pregnancy-related complications such as gestational diabetes or even excessive fetal weight gain, which can reduce the chances of natural child-birth [88].

## Diet and nutrition during pregnancy

Nutritional needs change throughout pregnancy, however, general healthy eating principles still apply and should be followed (e.g., Healthy Eating Plate food guide).

Protein is an important nutrient during pregnancy. The recommended daily intake of protein in adult women is 0.8 g/kg [e.g., a 60 kg woman would need

about 48 g of protein per day (60 x 0.8 = 48 g)], while in pregnant women protein needs increase to 1.1 g/kg [e.g., a 60 kg pregnant woman would need about 66 g of protein per day (60 x 1.1 = 66 g]. Protein sources can be separated in animal and plant proteins, some examples of animal protein sources are meat, fish, eggs and dairy products and some examples of plant protein sources are legumes such as beans, chickpeas or lentils and nuts such as almonds, peanuts or pine nuts.

About half of daily energy needs during pregnancy should come from carbohydrates. Carbohydrates-rich foods such as whole grain cereals, fruits, legumes and vegetables are some examples of healthy options, which can help to reach the carbohydrates daily goal. Despite the fact sugar-rich foods (e.g., sweet desserts, chocolates, sweet cookies) are also sources of carbohydrates, its intake should be limited. If the pregnant woman has diabetes, or if she develops gestational diabetes during pregnancy, implementing personalized dietary advice/plan is essential, mostly in cases which insulin therapy is necessary, because just with a personalized evaluation, adaptation and distribution of the nutritional needs and intake (e.g., carbohydrates intake), the best glycaemic control (diabetes control) can be achieved.

Fat intake during pregnancy, and most of all, fat quality intake, is also important because the development of the brain and the retina (located in the eyes and essential for vision) depends, in part, on polyunsaturated fatty acids, as omega-3 and omega-6 fatty acids.

Foods such as olive oil, fish in general, seeds, oilseed nuts and avocado are some examples of foods that may help you achieve the recommended daily doses of these nutrients - 1.4 g per day of omega-3 and 13 g per day of omega-6.

Assuming that during pregnancy is present a healthy and balanced diet and therefore the intake of all the main nutrients is sufficient and balanced, it is not necessary to increase the consumption of nutrients as calcium or phosphorus, because women's body itself is able to manage these nutrients more efficiently (e.g., during pregnancy, the pregnant woman produces several hormones that allow her to absorb greater amounts of calcium from the food she eats). On the other hand, there are nutrients whose recommended amounts increase considerably during pregnancy, and it is sometimes necessary to supplement those

nutrients to reach the recommended values and avoid deficits (e.g., iron needs during pregnancy are 150% higher than in an adult women of childbearing age). Table 4 shows the recommended daily intake of some micronutrients (vitamins and minerals) for adult women of childbearing age and for pregnant women.

Table 4. Recommended daily intake for adult women of childbearing age and for pregnant women, of some micronutrients.

| Nutrient | Women of childbearing age | Pregnant women |
|---|---|---|
| Vitamin A (μg/day) | 700 | 770 |
| Vitamin D (μg/day) | 15 | 15 |
| Vitamin E (mg/day) | 15 | 15 |
| Vitamin K (μg/day) | 90 | 90 |
| Folate (μg/day) | 400 | 600 |
| Niacin (mg/day) | 14 | 18 |
| Riboflavin (mg/day) | 1,1 | 1,4 |
| Thiamine (mg/day) | 1,1 | 1,4 |
| Vitamin B6 (mg/day) | 1,3 | 1,9 |
| Vitamin B12 (μg/day) | 2,4 | 2,6 |
| Vitamin C (mg/day) | 75 | 85 |
| Calcium (mg/day) | 1000 | 1000 |
| Iron (mg/day) | 18 | 27 |
| Phosphorous (mg/day) | 700 | 700 |
| Iodine (μg/day) | 150 | 220 |
| Magnesium (mg/day) | 320 | 350 |
| Potassium (mg/day) | 4700 | 4700 |
| Selenium (μg/day) | 55 | 60 |
| Zinc (mg/day) | 8 | 11 |

The use of multivitamin and multimineral supplements during pregnancy is a growing practice worldwide, however, it is recommended that the pregnant woman obtain most of the necessary nutrients through food, and if necessary, to use these "aids" to achieve all nutritional goals. It is important to emphasize that supplementation with folic acid, iodine or iron are usually recommend during pregnancy, but this supplementation should be through well-regulated drugs containing micronutrients and not through less regulated food supplements (usually with multiple nutrients). In most countries, food supplements are not pharmacologically regulated and have a high risk of adulteration and contamination.

For instance, folic acid and iodine supplementation can prevent several fetal problems hence its supplementation is recommended during pregnancy by reducing the risk associated to these nutrients' deficits. Especially in the second and third trimesters of pregnancy, iron supplementation is also often recommended since it is difficult to reach the high daily iron needs just with a regular diet.

To increase iron usage by the pregnant women body, simple changes as adding vitamin C rich foods (e.g. kiwi, strawberry, pepper, kale, papaya, orange) to iron rich foods (e.g. cinnamon, clams, liver, beans, lentils, meat), as it enhances the absorption of iron; and avoid a concomitant consumption of iron rich foods with beverages such as tea and coffee, as they inhibit the absorption of iron, are some useful advices.

## Hydration

Hydration status is of paramount importance during pregnancy. Pregnant women should drink water before, during and after exercise. Usually, it is advised that the pregnant women should drink about 1.7 to 3 L/day, but since factors as temperature, humidity, clothing and sweating (highly influenced by exercise) influence dehydration, recommendations should be individualized considering the previous factors. Usual dietary intake should also be considered while defining the amount of liquids that a pregnant women should have, because almost every food has relevant amounts of water in its constitution. In the case of a pregnant exerciser an example of a simple hydration plan can be the intake of about 0.5 L of liquids about 30 to 60 minutes before starting to exercise, the intake of 0.5 to 1.2 L per hour of exercise and a reposition of 1.5 to 2 L per each 1 L of

sweat lost during the exercise session. Sweat loss during an exercise session can be estimated by weighing without clothing before exercising and weighing again without clothing after exercise (after drying with a towel). The weight difference will be mainly due to the sweat loss. If liquids were intake during the exercise session, the amount of liquids intake should be deducted from the result (e.g., if a woman has 60 kg before exercise and drank 0.5 L of water during the exercise session, and at the end of the exercise session has 59.5 kg, it means that the sweat loss was about 1 L, i.e., 60 - 59.5 + 0.5 of water ≈ 1 L of sweat loss; therefore, after physical exercise between 1.5 to 2 L of fluids would be adequate to compensate the sweat lost).

Hydration is also a key factor in a condition that is estimated to affect more than a third of pregnant women: constipation. Constipation is also aggravated if there is a need to use iron supplements because constipation is one of the common adverse effects of iron supplementation. If there is constipation, the triad: drinking sufficient fluids, sufficient intake of dietary fiber (e.g., through foods such as whole grains and derivatives, vegetables, seeds, fresh and dried fruits such as kiwi, orange, fig and plum) and regular and sufficient physical activity seem to be the most efficient lifestyle approach. If, on the other hand, diarrhea is present hydration and electrolytes needs increases enormously in order to compensate for the acute water and electrolyte losses.

Other issue related with hydration is UI. UI during exercise is due to mechanical and anatomical changes. Considerations exist to minimize UI during exercise, such as: voiding before activity; avoiding breath holding and use of Valsalva maneuver during exercise; practicing pelvic muscle-strengthening exercises and; minimizing high-impact activities when incontinence symptoms appear; check if a bathroom is available in proximity of the training location.

Since competitive athletes have a strenuous training schedule throughout pregnancy and resume high-intensity postpartum training sooner as compared to other pregnant women, they require frequent and closer supervision [42]. Such athletes should pay particular attention to avoid hyperthermia, maintain proper hydration, and sustain adequate caloric intake to prevent weight loss, which may adversely affect fetal growth [42].

## Food safety during pregnancy

Pregnancy is one of the stages of life where a food-borne infection can bring more serious consequences and that is one of the reasons why supplementary care is usually recommended to pregnant women. These supplementary care starts on the food sources choice and should go until its preparation, cooking and even storing (e.g., refrigerating). Bacteria like Salmonella or Listeria, or parasites like Toxoplasma can terribly affect the course of pregnancy. Simple advice as the following will reduce the risk of contact with these and other microorganisms that can be especially dangerous during pregnancy:

| |
|---|
| Properly wash and disinfect hands always before and after handling food, after going to the toilet, after being in contact with animals, or after being in contact with places where animals may have been (e.g., if you are gardening, handling earth or sand) |
| Wash and properly disinfect the vegetables and fruits you eat |
| Separate raw food from cooked or previously washed and disinfected food, in order to avoid cross contamination (e.g., never place cooked food in a container where raw food was previously, without properly washing and disinfecting the container) |
| If you leave food to defrost in the refrigerator (e.g., meat or fish) always place them in closed containers to prevent the liquid resulting from the defrosting from spilling and potentially contaminate other foods |
| Ensure that the food is properly defrosted before cooking it |
| Properly cook food (i.e., get to an adequate cooking temperature during the appropriate time), especially foods such as meat, fish or eggs (always opt for the "well-done" options) |
| If you reheat the food (leftovers), make sure it gets to boiling temperature, or if you use the microwave assure they are well cooked internally |
| Always check and respect the expiration date of the food |
| Be careful with interactions with cats and places where they may have been, as cat feces may be contaminated with Toxoplasma. If you are gardening or otherwise have to handle cat feces (e.g., if you have a cat) always wear gloves |

# SLEEP RECOMMENDATIONS DURING PREGNANCY

# 10

Sleep is a precious asset that unfortunately is still very despised in today's society, the so-called civilized society, the one that doesn't sleep - the 24 hour one. While sleep deprivation is common during pregnancy, and more pronounced in the postpartum period, it is even more increased due to the continuous care that the mother must provide to her newborn baby. In addition, daily needs are increased from the first trimester of pregnancy, and daytime sleepiness is easily evident [121-124]. However, if sleep is insufficient, in duration and quality, the health and general well-being of the pregnant woman may be compromised, that is sleep deprivation can negatively affect her performance in daily tasks, maternal health (through increased blood pressure, and the risk of eclampsia, caesarean and depressive symptoms), and also her future ability to produce milk and, therefore, to breastfeed [124-127].

Still considering sleep during pregnancy, it can also add an important risk factor, namely the fact that the pregnant woman is an adolescent. In this younger age-group, energy and nutritional needs are increased, the sleep pattern is altered (due to the neurophysiological components of the body associated with sleep and circadian rhythm) and the risk of developing malnutrition may exist, especially in pregnant women with low socioeconomic status [123,128].

In pregnancy, the most frequent sleep disorders are insomnia, restless legs syndrome and sleep apnea [129].

Insomnia is characterized by the inability to initiate or maintain sleep, that is the pregnant woman wakes up several times before waking up and is unable to continue to sleep, which can result in excessive daytime sleepiness and, consequently, functional impairment during all day [123]

Restless legs syndrome is two to three times more prevalent in pregnant women than in the general population [130]. It is characterized by an unpleasant

sensation that leads to sudden movement of the legs, often associated with pain, making it difficult to sleep. These symptoms can be alleviated by moving the legs, for example, by walking.

In pregnancy, sleep apnea is associated with overweight or obesity in women. There is a momentary interruption in breathing, which may be associated with snoring; these symptoms should be evaluated and monitored during pregnancy [129].

However, there are daily habits and behaviours that help to minimize symptoms or even improve sleep quality during pregnancy, namely [123,130]:

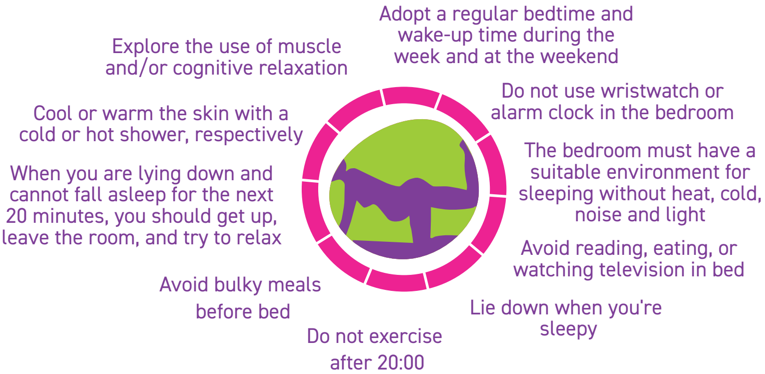

# References


1. Bull FC, Al-Ansari SS, Biddle S, et al. World Health Organization 2020 guidelines on physical activity and sedentary behavior. British Journal of Sports Medicine. 2020;54:1451--62. Available at: https://bjsm.bmj.com/content/54/24/1451.long

2. Santos-Rocha R (Editor). Exercise and Physical Activity during Pregnancy and Postpartum. Evidence-Based Guidelines. 2 ed. Switzerland: Springer International Publishing, 2022.

3. Caspersen CJ, Powell KE, Christenson GM. Physical activity, exercise, and physical fitness: definitions and distinctions for health-related research. Public Health Rep. 1985;100(2):126–31.

4. ACSM - American College of Sports Medicine. ACSM's Resources for the Exercise Physiologist (2nd ed). Wolters Kluwer, 2018.

5. ACSM - American College of Sports Medicine. ACSM's Guidelines for Exercise Testing and Prescription. 11 ed. Wolters Kluwer Health, 2021.

6. Kohl HW 3rd, Craig CL, Lambert EV, Inoue S, Alkandari JR, Leetongin G, Kahlmeier S; Lancet Physical Activity Series Working Group. The pandemic of physical inactivity: global action for public health. Lancet 2012;380(9838):294-305.

7. Blair SN. Physical inactivity: the biggest public health problem of the 21st century. Br J Sports Med. 2009;43:1-2.

8. Barakat R, Perales M, Bacchi M, Coteron J, Refoyo I. A program of exercise throughout pregnancy. Is it safe to mother and newborn? Am J Health Promot 2013;29(1): 2-8.

9. Perales M, Santos-Lozano A, Ruiz JR, Lucia A, Barakat R. Benefits of aerobic or resistance training during pregnancy on maternal health and perinatal outcomes: A systematic review. Early Hum Dev. 2016 Mar;94:43-8.

10. Dipietro L, Evenson KR, Bloodgood B, Sprow K, Troiano RP, Piercy KL, Vaux-Bjerke A, Powell KE; 2018 PHYSICAL ACTIVITY GUIDELINES ADVISORY COMMITTEE*. Benefits of Physical Activity during Pregnancy and Postpartum: An Umbrella Review. Med Sci Sports Exerc. 2019 Jun;51(6):1292-302.

11. Díaz-Burrueco JR, Cano-Ibáñez N, Martín-Peláez S, Khan KS, Amezcua-Prieto C. Effects on the maternal-fetal health outcomes of various physical activity types in healthy pregnant women. A systematic review and meta-analysis. Eur J Obstet Gynecol Reprod Biol 2021;262:203-215.diet and exercise interventions for preventing gestational diabetes mellitus. Cochrane Database Syst Rev. 2017 Nov 13;11(11):CD010443.

12. Davenport MH, Skow RJ, Steinback CD. Maternal Responses to Aerobic Exercise in Pregnancy. Clin Obstet Gynecol. 2016 Sep;59(3):541-51.

13. Morales-Suárez-Varela M, Clemente-Bosch E, Peraita-Costa I, Llopis-Morales A, Martínez I, Llopis-González A. Maternal Physical Activity During Pregnancy and the Effect on the Mother and Newborn: A Systematic Review. J Phys Act Health. 2020 Dec 22;18(1):130-47.

14. Magro-Malosso ER, Saccone G, Di Tommaso M, Roman A, Berghella V. Exercise during pregnancy and risk of gestational hypertensive disorders: a systematic review and meta-analysis. Acta Obstet Gynecol Scand. 2017 Aug;96(8):921-31.

15. Davenport MH, Ruchat SM, Poitras VJ, Jaramillo Garcia A, Gray CE, Barrowman N, Skow RJ, Meah VL, Riske L, Sobierajski F, James M, Kathol AJ, Nuspl M, Marchand AA, Nagpal TS, Slater LG, Weeks A, Adamo KB, Davies GA, Barakat R, Mottola MF. Prenatal exercise for the prevention of gestational diabetes mellitus and hypertensive disorders of pregnancy: a systematic review and meta-analysis. Br J Sports Med. 2018 Nov;52(21):1367-75.

16. Harrison AL, Shields N, Taylor NF, Frawley HC. Exercise improves glycaemic control in women diagnosed with gestational diabetes mellitus: a systematic review. J Physiother. 2016 Oct;62(4):188-96.

17. Bgeginski, R., Ribeiro, P. A.B., Mottola, M. F. and Ramos, J. G. L. (2017), Effects of weekly supervised exercise or physical activity counseling on fasting blood glucose in women diagnosed with gestational diabetes mellitus: A systematic review and meta-analysis of randomized trials. Journal of Diabetes, 9: 1023–32

18. Brown J, Ceysens G, Boulvain M. Exercise for pregnant women with gestational diabetes for improving maternal and fetal outcomes. Cochrane Database Syst Rev. 2017 Jun 22;6(6):-CD012202.

19. Shepherd E, Gomersall JC, Tieu J, Han S, Crowther CA, Middleton P. Combined diet and exercise interventions for preventing gestational diabetes mellitus. Cochrane Database Syst Rev. 2017 Nov 13;11(11):CD010443.

20. Mørkved S, Bø K. Effect of pelvic floor muscle training during pregnancy and after childbirth on prevention and treatment of urinary incontinence: a systematic review. Br J Sports Med. 2014 Feb;48(4):299-310.

21. Domenjoz I, Kayser B, Boulvain M. Effect of physical activity during pregnancy on mode of delivery. Am J Obstet Gynecol. 2014 Oct;211(4):401.e1-11.

22. Liddle SD, Pennick V. Interventions for preventing and treating low-back and pelvic pain during pregnancy. Cochrane Database of Systematic Reviews 2015, Issue 9. Art. No.: CD001139.

23. Davenport MH, Marchand AA, Mottola MF, Poitras VJ, Gray CE, Jaramillo Garcia A, Barrowman N, Sobierajski F, James M, Meah VL, Skow RJ, Riske L, Nuspl M, Nagpal TS, Courbalay A, Slater LG, Adamo KB, Davies GA, Barakat R, Ruchat SM. Exercise for the prevention and treatment of low back, pelvic girdle and lumbopelvic pain during pregnancy: a systematic review and meta-analysis. Br J Sports Med. 2019 Jan;53(2):90-98.

24. Shiri R, Coggon D, Falah-Hassani K. Exercise for the prevention of low back and pelvic girdle pain in pregnancy: A meta-analysis of randomized controlled trials. Eur J Pain. 2018 Jan;22(1):19-27.

25. Daley AJ, Foster L, Long G, Palmer C, Robinson O, Walmsley H, Ward R. The effectiveness of exercise for the prevention and treatment of antenatal depression: systematic review with meta-analysis. BJOG 2015 Jan; 122(1):57-62.

26. McCurdy AP, Boulé NG, Sivak A, Davenport MH. Effects of Exercise on Mild-to-Moderate Depressive Symptoms in the Postpartum Period: A Meta-analysis. Obstet Gynecol. 2017 May 5.

27. Davenport MH, McCurdy AP, Mottola MF, Skow RJ, Meah VL, Poitras VJ, Jaramillo Garcia A, Gray CE, Barrowman N, Riske L, Sobierajski F, James M, Nagpal T, Marchand AA, Nuspl M, Slater LG, Barakat R, Adamo KB, Davies GA, Ruchat SM. Impact of prenatal exercise on both prenatal and postnatal anxiety and depressive symptoms: a systematic review and meta-analysis. Br J Sports Med. 2018 Nov;52(21):1376-1385.

28. Nakamura A, van der Waerden J, Melchior M, Bolze C, El-Khoury F, Pryor L. Physical activity during pregnancy and postpartum depression: Systematic review and meta-analysis. J Affect Disord. 2019 Mar 1;246:29-41.

29. Bø K, Artal R, Barakat R, Brown W, Dooley M, Evenson KR, Haakstad LA, Larsen K, Kayser B, Kinnunen TI, Mottola MF, Nygaard I, van Poppel M, Stuge B, Davies GA; IOC Medical Commission. Exercise and pregnancy in recreational and elite athletes: 2016 evidence summary from the IOC expert group meeting, Lausanne. Part 2-the effect of exercise on the fetus, labour and birth. Br J Sports Med. 2016 Oct 12. pii: bjsports-2016--096810.

30. Tanha FD, Ghajarzadeh M, Mohseni M, Shariat M, Ranjbar M. Is ACOG Guideline Helpful for Encouraging Pregnant Women to Do Exercise During Pregnancy? Acta Medica Iranica. 2014;52(6):458-61.

31. Savvaki D, Taousani E, Goulis DG, Tsirou E, Voziki E, Douda H, Nikolettos N, Tokmakidis SP. Guidelines for exercise during normal pregnancy and gestational diabetes: a review of international recommendations. Hormones (Athens). 2018 Dec;17(4):521-529.

32. Evenson KR, Mottola MF, Artal R. Review of Recent Physical Activity Guidelines During Pregnancy to Facilitate Advice by Health Care Providers. Obstet Gynecol Surv. 2019 Aug;74(8):481-9.

33. Tsakiridis I, Bakaloudi DR, Oikonomidou AC, Dagklis T, Chourdakis M. Exercise during pregnancy: a comparative review of guidelines. J Perinat Med. 2020 Jul 28;48(6):519-525.

34. Szumilewicz A, Worska A, Santos-Rocha R, Oviedo-Caro MA. Evidence-based and practice-oriented guidelines for exercising during pregnancy. In R. Santos-Rocha, Editor, Exercise and Physical Activity during Pregnancy and Postpartum. Evidence-Based Guidelines. 2 ed. Switzerland: Springer International Publishing; 2022, Ch.7.

35. SMA – Sports Medicine Australia. Pregnancy and Exercise. Women in Sport. 2017. Available at: https://sma.org.au/sma-site-content/uploads/2017/08/SMA-Position-Statement-Exercise-Pregnancy.pdf

36. Bø K, Artal R, Barakat R, Brown WJ, Davies GAL, Dooley M, et al. Exercise and pregnancy in recreational and elite athletes: 2016/2017 evidence summary from the IOC expert group meeting, Lausanne. Part 5 - Recommendations for health professionals and active women. British journal of sports medicine. 2018;52(17):1080-5. Available at: https://bjsm.bmj.com/content/52/17/1080.long

37. Bø K, Artal R, Barakat R, Brown WJ, Davies GAL, Dooley M, Evenson KR, Haakstad LAH, Kayser B, Kinnunen TI, Larsén K, Mottola MF, Nygaard I, van Poppel M, Stuge B, Khan KM; IOC Medical Commission. Exercise and pregnancy in recreational and elite athletes: 2016/17 evidence summary from the IOC Expert Group Meeting, Lausanne. Part 3 - Exercise in the postpartum period. Br J Sports Med. 2017 Nov;51(21):1516-25. Available at: https://bjsm.bmj.com/content/51/21/1516.long

38. Mottola MF, Davenport MH, Ruchat S-M, Davies GA, Poitras VJ, Gray CE, et al. 2019 Canadian guideline for physical activity throughout pregnancy. British Journal of Sports Medicine. 2018;52(21):1339-46. Available at: https://bjsm.bmj.com/content/52/21/1339

39. USDHHS - U.S. Department of Health and Human Services. Physical Activity Guidelines for Americans. 2 ed. Washington, DC: U.S. Department of Health and Human Services; 2018. Available at: https://health.gov/sites/default/files/2019-09/Physical_Activity_Guidelines_2nd_edition.pdf

40. EIM/ACSM. Being Active during Pregnancy. Exercise is Medicine/American College of Sports Medicine 2019. Available at: https://www.exerciseismedicine.org/assets/page_documents/EIM_Rx%20for%20Health_Pregnancy.pdf

41. NHS - National Health Service. Exercise in Pregnancy; 2020. Available at: https://www.nhs.uk/pregnancy/keeping-well/exercise/

42. ACOG – American College of Obstetricians and Gynecologists. ACOG Committee Opinion No. 804: Physical Activity and Exercise During Pregnancy and the Postpartum Period. Obstetrics and Gynecology [Internet]. 2020; 135(4):[e178-e88 pp.]. Available at: https://www.acog.org/clinical/clinical-guidance/committee-opnion/articles/2020/04/physical-activity-and-exercise-during-pregnancy-and-the-postpartum-period

43. ACSM - American College of Sport Medicine. ACSM information on Pregnancy Physical Activity. American College of Sports Medicine; 2020. Available at: https://www.acsm.org/docs/default-source/files-for-resource-library/pregnancy-physical-activity.pdf?sfvrsn=12a73853_4

44. RANZCOG - The Royal Australian and New Zealand College of Obstetricians and Gynaecologists. Exercise in Pregnancy. RANZCOG, 2020. Available at: https://ranzcog.edu.au/womens-health/patient-information-resources/exercise-during-pregnancy

45. AGDH - Australian Government. Department of Health. Guidelines for physical activity during pregnancy, 2021. Available at: https://www.health.gov.au/resources/publications/physical-activity-and-exercise-during-pregnancy-guidelines-brochure

46. Campos MDSB, Buglia S, Colombo CSSS, Buchler RDD, Brito ASX, Mizzaci CC, Feitosa RHF, Leite DB, Hossri CAC, Albuquerque LCA, Freitas OGA, Grossman GB, Mastrocola LE. Position Statement on Exercise During Pregnancy and the Post-Partum Period - 2021. Arq Bras Cardiol. 2021 Jul;117(1):160-80. English, Portuguese. Available at: https://www.ncbi.nlm.nih.gov/pmc/articles/PMC8294738/#S01

47. CSEP - Canadian Society for Exercise Physiology. GET ACTIVE QUESTIONNAIRE FOR PREGNANCY. 2021 Available at: https://csep.ca/wp-content/uploads/2021/05/GAQ_P_English.pdf

48. CSEP - Canadian Society for Exercise Physiology. HEALTH CARE PROVIDER CONSULTATION FORM FOR PRENATAL PHYSICAL ACTIVITY. 2021 Available at: https://csep.ca/wp-content/uploads/2021/05/GAQ_P_HCP_English.pdf

49. Foxcroft KF, Callaway LK, Byrne NM, Webster J. Development and validation of a pregnancy symptoms inventory. BMC Pregnancy Childbirth. 2013 Jan 16;13:3.

50. PAR-Q+ Collaboration. Physical Activity Readiness Questionnaire for Everyone (PAR-Q+), 2017. Available at: http://eparmedx.com/

51. Tang MSS, Moore K, McGavigan A, Clark RA, Ganesan AN. Effectiveness of Wearable Trackers on Physical Activity in Healthy Adults: Systematic Review and Meta-Analysis of Randomized Controlled Trials. JMIR Mhealth Uhealth. 2020 Jul 22;8(7):e15576.

52. Chasan-Taber L, Schmidt MD, Roberts DE, Hosmer D, Markenson G, Freedson PS. Development and validation of a Pregnancy Physical Activity Questionnaire. Med Sci Sports Exerc., 2004; 36(10):1750-60.

53. Santos-Rocha R, Corrales-Gutierrez I, Szumilewicz A, Pajaujiene S. Exercise testing and prescription during pregnancy. In R. Santos-Rocha, Editor, Exercise and Physical Activity during Pregnancy and Postpartum. Evidence-Based Guidelines. 2 ed. Switzerland: Springer International Publishing; 2022, Ch.8.

54. ASC - Australian Sports Commission. Pregnancy and Sport: Guidelines for the Australian sporting industry, 2002.

55. Szymanski LM, Satin AJ. Strenuous exercise during pregnancy: is there a limit? Am J Obstet Gynecol. 2012 Sep;207(3):179.e1-6.

56. Pivarnik JM, Szymanski LM, Conway MR. The Elite Athlete and Strenuous Exercise in Pregnancy. Clin Obstet Gynecol. 2016 Sep;59(3):613-9.

57. Wowdzia JB, Davenport MH. Cardiopulmonary exercise testing during pregnancy. Birth Defects Res. 2021 Feb 1;113(3):248-64.

58. Mottola MF, Davenport MH, Brun CR, Inglis SD, Charlesworth S, Stopper MM. VO2peak prediction and exercise prescription for pregnant women. Med Sci Sports Exerc. 2006; 38(8):1389-95.

59. Gellish RL, Goslin BR, Olson RE, McDonald A, Russi GD, Moudgil VK. Longitudinal modeling of the relationship between age and maximal heart rate. Med Sci Sports Exerc. 2007;39(5):822–9.

60. Dennis AT, Salman M, Paxton E, Flint M, Leeton L, Roodt F, Yentis S, Dyer RA. Resting Hemodynamics and Response to Exercise Using the 6-Minute Walk Test in Late Pregnancy: An International Prospective Multicentre Study. Anesth Analg. 2019 Aug;129(2):450-7.

61. Fitzgerald CM and Segal NA (eds.), Musculoskeletal Health in Pregnancy and Postpartum, 2015. Springer International Publishing Switzerland.

62. Ortega FB, Ruiz JR, España-Romero V, Vicente-Rodriguez G, Martínez-Gómez D, Manios Y, Béghin L, Molnar D, Widhalm K, Moreno LA, Sjöström M, Castillo MJ; HELENA study group. The International Fitness Scale (IFIS): usefulness of self-reported fitness in youth. Int J Epidemiol. 2011 Jun;40(3):701-11.

63. Romero-Gallardo L, Soriano-Maldonado A, Ocón-Hernández O, Acosta-Manzano P, Coll-Risco I, Borges-Cosic M, Ortega FB, Aparicio VA. International Fitness Scale-IFIS: Validity and association with health-related quality of life in pregnant women. Scand J Med Sci Sports. 2020 Mar;30(3):505-14.

64. Henström M, Leppänen MH, Henriksson P, Söderström E, Sandborg J, Ortega FB, Löf M. Self-reported (IFIS) versus measured physical fitness, and their associations to cardiometabolic risk factors in early pregnancy. Sci Rep. 2021 Nov 22;11(1):22719.

65. Pimenta N and van Poppel M. Body composition changes during pregnancy and effects of physical exercise. In Santos-Rocha, Editor, Exercise and Physical Activity during Pregnancy and Postpartum. Evidence-Based Guidelines. 2 ed. Switzerland: Springer International Publishing; 2022, Ch.4

66. Silva MRG, Rodriguez Doñate B, Che Carballo KN. Nutritional requirements for the pregnant exerciser and athlete. In R. Santos-Rocha, Editor, Exercise and Physical Activity during Pregnancy and Postpartum. Evidence-Based Guidelines. 2 ed. Switzerland: Springer International Publishing; 2022, Ch.13.

67. Jorge R, Teixeira D, Ferreira I, Alvarez-Fálcon AL. Diet Recommendations for the Pregnant Exerciser and Athlete. In R. Santos-Rocha, Editor, Exercise and Physical Activity during Pregnancy and Postpartum. Evidence-Based Guidelines. 2 ed. Switzerland: Springer International Publishing; 2022, Ch.14.

68. Szumilewicz A and Santos-Rocha R. Exercise selection during pregnancy. In Santos-Rocha, Editor, Exercise and Physical Activity during Pregnancy and Postpartum. Evidence-Based Guidelines. 2 ed. Switzerland: Springer International Publishing; 2022, Ch.9.

69. Active Pregnancy YouTube Chanel: https://www.youtube.com/channel/UC0Vyookwc0mcQ5T70imtoNA/playlists

70. Garber CE, Blissmer B, Deschenes MR, Franklin BA, Lamonte MJ, Lee IM, Nieman DC, Swain DP; American College of Sports Medicine. American College of Sports Medicine position stand. Quantity and quality of exercise for developing and maintaining cardiorespiratory, musculoskeletal, and neuromotor fitness in apparently healthy adults: guidance for prescribing exercise. Med Sci Sports Exerc. 2011 Jul;43(7):1334-59.

71. Perales M, Nagpal TS, Barakat R. Physiological changes during pregnancy. Main adaptations and discomforts and implications for physical activity and exercise. In Santos-Rocha, Editor, Exercise and Physical Activity during Pregnancy and Postpartum. Evidence-Based Guidelines. 2 ed. Switzerland: Springer International Publishing; 2022, Ch.3.

72. Bø K, Stuge B, Hilde G. Specific musculoskeletal adaptations in pregnancy: pelvic floor, pelvic girdle and low back pain. Implications for physical activity and exercise. In Santos-Rocha, Editor, Exercise and Physical Activity during Pregnancy and Postpartum. Evidence-Based Guidelines. 2 ed. Switzerland: Springer International Publishing; 2022, Ch.6.

73. Branco M, Santos-Rocha R, Aguiar L, Vieira F, Veloso AP. Biomechanical adaptations of gait in pregnancy. Implications for physical activity and exercise. In Santos-Rocha, Editor, Exercise and Physical Activity during Pregnancy and Postpartum. Evidence-Based Guidelines. 2 ed. Switzerland: Springer International Publishing; 2022, Ch.5.

74. ACOG. Your Pregnancy and Childbirth. 7 ed. American College of Obstetricians and Gynecologists, 2021.

75. Miquelutti MA, Cecatti JG, Makuch MY. Evaluation of a birth preparation program on lumbopelvic pain, urinary incontinence, anxiety and exercise: a randomized controlled trial. BMC Pregnancy Childbirth. 2013 Jul 29;13:154.

76. Miquelutti MA, Cecatti JG, Makuch MY. Developing strategies to be added to the protocol for antenatal care: an exercise and birth preparation program. Clinics (Sao Paulo). 2015 Apr;70(4):231-6.

77. Akca A, Corbacioglu Esmer A, Ozyurek ES, Aydin A, Korkmaz N, Gorgen H, Akbayir O. The influence of the systematic birth preparation program on childbirth satisfaction. Arch Gynecol Obstet. 2017 May;295(5):1127-33.

78. Ribeiro MM, Andrade A, Nunes I. Physical exercise in pregnancy: benefits, risks and prescription. J Perinat Med. 2021 Sep 6.

79. Sivan E, Homko CJ, Chen XH, Reece EA, Boden G. Effect of insulin on fat metabolism during and after normal pregnancy. Diabetes. 1999;48(4):834-8.

80. Zhang CL, Ning Y. Effect of dietary and lifestyle factors on the risk of gestational diabetes: review of epidemiologic evidence. American Journal of Clinical Nutrition. 2011;94(6):1975S-9S.

81. Pettit D, Bennett PH, Knowler WC, et al. Gestational diabetes mellitus and impaired glucose tolerance during pregnancy: long-term effects on obesity and glucose intolerance in the offspring. Diabetes Care 1985;34:119–22.

82. Horvath K, Koch K, Jeitler K, et al. Effects of treatment in women with gestational diabetes mellitus: systematic review and meta-analysis. BMJ 2010;340:1395.

83. Carolan-Olah MC. Educational and intervention programmes for gestational diabetes mellitus (GDM) management: an integrative review. Collegian 2016;23:103–14.

84. Sanabria-Martínez G, García-Hermoso A, Poyatos-León R, Álvarez-Bueno C, Sánchez--López M, Martínez-Vizcaíno V. Effectiveness of physical activity interventions on preventing gestational diabetes mellitus and excessive maternal weight gain: a meta-analysis. BJOG. 2015 Aug;122(9):1167-74.

85. Brown J, Ceysens G, Boulvain M. Exercise for pregnant women with gestational diabetes for improving maternal and fetal outcomes. Cochrane Database Syst Rev. 2017 Jun 22;6(6):-CD012202.

86. Shepherd E, Gomersall JC, Tieu J, Han S, Crowther CA, Middleton P. Combined diet and exercise interventions for preventing gestational diabetes mellitus. Cochrane Database Syst Rev. 2017 Nov 13;11(11):CD010443.

87. Choi J, Fukuoka Y, Lee JH. The effects of physical activity and physical activity plus diet interventions on body weight in overweight or obese women who are pregnant or in postpartum: a systematic review and meta analysis of randomized controlled trials. Preventive Medicine. 2013;56(6):351-64.

88. ACOG - American College of Obstetricians and Gynecologists. Obesity and Pregnancy. ACOG Committee opinion Nº 549. American College of Obstetricians and Gynecologists. Obstet Gynecol 2013; 121:213-7.

89. Mottola MF, Giroux I, Gratton R, et al. Nutrition and exercise prevent excess weight gain in overweight pregnant women. Med Sci Sports Exerc. 2010;42(2):265–72.

90. International Weight Management in Pregnancy (i-WIP) Collaborative Group. Effect of diet and physical activity based interventions in pregnancy on gestational weight gain and pregnancy outcomes: meta-analysis of individual participant data from randomised trials. BMJ. 2017 Jul 19;358:j3119.

91. Say L, Chou D, Gemmill A, et al. Global causes of maternal death: a WHO systematic analysis. Lancet Glob Health 2014;2(6):e323–e333.

92. Gillon TE, Pels A, von Dadelszen P, et al. Hypertensive disorders of pregnancy: a systematic review of international clinical practice guidelines. PLoS One 2014;9(12):e113715.

93. Berzan E, Doyle R, Brown CM. Treatment of preeclampsia: current approach and future perspectives. Curr Hypertens Rep 2014;16(9):473.

94. Mparmpakas D, Goumenou A, Zachariades E, et al. Immune system function, stress, exercise and nutrition profile can affect pregnancy outcome: Lessons from a Mediterranean cohort. Exp Ther Med 2013;5(2):411–8.

95. Chawla S, Anim-Nyame N. Advice on exercise for pregnant women with hypertensive disorders of pregnancy. Int J Gynaecol Obstet. 2015 Mar;128(3):275-9.

96. Abu MA, Abdul Ghani NA, Lim Pei S, Sulaiman AS, Omar MH, Muhamad Ariffin MH, et al. Do exercises improve back pain in pregnancy? Hormone Molecular Biology & Clinical Investigation. 2017;32(3):1-7.

97. Sklempe Kokic I, Ivanisevic M, Uremovic M, Kokic T, Pisot R, Simunic B. Effect of therapeutic exercises on pregnancy-related low back pain and pelvic girdle pain: Secondary analysis of a randomized controlled trial. Journal of Rehabilitation Medicine. 2017;49(3):251-7.

98. Gavin N, Gaynes BN, Lohr KN. Meltzer-Brody S. Perinatal depression: a systematic review of prevalence and incidence. Obstet Gynecol 2005; 106(5):1071-83.

99. Bennett HA, Einarsson A, Taddio A. Prevalence of depression during pregnancy: systematic review. Obstet Gynecol 2006; 103(4):698-709.

100. Trivedi MH, Greer TL, Grannemann BD, Chambliss HO, Jordan AN: Exercise as an augmentation strategy for treatment of major depression. J Psychiatr Pract 2006; 12:205-–13.

101. Padmapriya N, Bernard J, Liang S, Loy S, Shen Z, Kwek K, et al. Association of physical activity and sedentary behavior with depression and anxiety symptoms during pregnancy in a multiethnic cohort of Asian women. Archives of Women's Mental Health. 2016;19(6):1119-28.

102. Yang X, Zhang A, Sayer L, Bassett S, Woodward S. The effectiveness of group-based pelvic floor muscle training in preventing and treating urinary incontinence for antenatal and postnatal women: a systematic review. Int Urogynecol J. 2021 Aug 28.

103. Woodley SJ, Lawrenson P, Boyle R, Cody JD, Mørkved S, Kernohan A, Hay-Smith EJC. Pelvic floor muscle training for preventing and treating urinary and faecal incontinence in antenatal and postnatal women. Cochrane Database Syst Rev. 2020 May 6;5(5):CD007471

104. Szumilewicz A, Dornowski M, Piernicka M, Worska A, Kuchta A, Kortas J, . . . Jastrzębski Z. High-Low Impact Exercise Program Including Pelvic Floor Muscle Exercises Improves Pelvic Floor Muscle Function in Healthy Pregnant Women – A Randomized Control Trial. Frontiers in Physiology. 2019, 9, 1867.

105. Schauberger CW, Rooney BL, Goldsmith L, Shenton D, Silva PD, Schaper A. Peripheral joint laxity increases in pregnancy but does not correlate with serum relaxin levels. American Journal Of Obstetrics And Gynecology. 1996;174(2):667-71.

106. Dumas GA, Reid JG. Laxity of knee cruciate ligaments during pregnancy. The Journal of Orthopaedic and Sports Physical Therapy. 1997;26(1):2-6.

107. Ebi KL, Capon A, Berry P, Broderick C, de Dear R, Havenith G, Honda Y, Kovats RS, Ma W, Malik A, Morris NB, Nybo L, Seneviratne SI, Vanos J, Jay O. Hot weather and heat extremes: health risks. Lancet. 2021 Aug 21;398(10301):698-708.

108. Dervis S, Dobson KL, Nagpal TS, Geurts C, Haman F, Adamo KB. Heat loss responses at rest and during exercise in pregnancy: A scoping review. J Therm Biol. 2021 Jul;99:103011.

109. Branco M, Santos-Rocha R, Vieira F. Biomechanics of gait during pregnancy. Scientific World Journal. 2014;2014:527940.

110. Patterson R, McNamara E, Tainio M, de Sa TH, Smith AD, Sharp SJ, et al. Sedentary behaviour and risk of all-cause, cardiovascular and cancer mortality, and incident type 2 diabetes: a systematic review and dose response meta analysis. Eur J Epidemiol. 2018;33(9):811–29.

111. Fazzi C, Saunders DH, Linton K, Norman JE, Reynolds RM. Sedentary behaviours during pregnancy: a systematic review. Int J Behav Nutr Phys Act. 2017 Mar 16;14(1):32.

112. Barone Gibbs B, Jones MA, Jakicic JM, Jeyabalan A, Whitaker KM, Catov JM. Objectively Measured Sedentary Behavior and Physical Activity Across 3 Trimesters of Pregnancy: The Monitoring Movement and Health Study. J Phys Act Health. 2021 Jan 28;18(3):254-61.

113. Di Fabio DR, Blomme CK, Smith KM, Welk GJ, Campbell CG. Adherence to physical activity guidelines in mid-pregnancy does not reduce sedentary time: an observational study. Int J Behav Nutr Phys Act. 2015 Feb 24;12:27.

114. Oviedo-Caro MÁ, Bueno-Antequera J, Munguía-Izquierdo D. Associations of 24-hours activity composition with adiposity and cardiorespiratory fitness: The PregnActive project. Scand J Med Sci Sports. 2020 Feb;30(2):295-302.

115. Canadian Society for Exercise Physiology. Canadian 24-Hour Movement Guidelines: An Integration of Physical Activity, Sedentary Behaviour, and Sleep. 2021. Available at: https://csepguidelines.ca/guidelines/adults-18-64/

116. Kozey-Keadle S, Libertine A, Lyden K, Staudenmayer J, Freedson PS. Validation of wearable monitors for assessing sedentary behavior. Med Sci Sports Exerc. 2011 Aug;43(8):1561-7.

117. Barone Gibbs B, Paley JL, Jones MA, Whitaker KM, Connolly CP, Catov JM. Validity of self-reported and objectively measured sedentary behavior in pregnancy. BMC Pregnancy Childbirth. 2020 Feb 11;20(1):99.

118. Rosenberg DE, Norman GJ, Wagner N, Patrick K, Calfas KJ, Sallis JF. Reliability and validity of the Sedentary Behavior Questionnaire (SBQ) for adults. J Phys Act Health. 2010 Nov;7(6):697-705.

119. Oviedo-Caro MÁ, Bueno-Antequera J, Munguía-Izquierdo D. Measuring Sedentary Behavior During Pregnancy: Comparison Between Self-reported and Objective Measures. Matern Child Health J. 2018 Jul;22(7):968-77.

120. Institute of Medicine (US) Panel on Micronutrients. Dietary Reference Intakes for Vitamin A, Vitamin K, Arsenic, Boron, Chromium, Copper, Iodine, Iron, Manganese, Molybdenum, Nickel, Silicon, Vanadium, and Zinc. Washington (DC): National Academies Press (US); 2001. 8, Iodine. Available from: https://www.ncbi.nlm.nih.gov/books/NBK222323/

121. Silva M-RG, Paiva T. Sono, Nutrição, Ritmo Circadiano, Jet Lag e Desempenho Desportivo. Lisboa: Federação de Ginástica de Portugal/ Instituto Português do Desporto e da Juventude I.P. 2015. ISBN: 978-989-8650-54-2.

122. Silva M-RG, Paiva T. Poor precompetitive sleep habits, nutrients' deficiencies, inappropriate body composition and athletic performance in elite gymnasts. Eur J Sport Sci 2016; 16(6):726-35.

123. Silva, M-RG, Paiva T. O sono durante a gravidez: neurofisiologia, distúrbios e higiene do sono. In Santos-Rocha R, Branco M (coords). Gravidez ativa: adaptações fisiológicas e biomecânicas durante a gravidez e no pós-parto. Lisboa: Fundação para a Ciência e a Tecnologia. 2016. ISBN: 978-989-8768-17-9 (edição digital); 978-989-8768-18-6 (edição impressa). 155-171.

124. Ramirez JO, Cabrera SA, Hidalgo H. et al.. Is preeclampsia associated with restless legs syndrome? Sleep Med 2013;14:894-6.

125. Vahdat M, Sariri E, Miri S. et al. Prevalence and associated features of restless legs syndrome in a population of Iranian women during pregnancy. Int J Gynecol Obstetrics 2013;123:46-9.

126. Wesstrom J, Skalkidou A, Manconi M. et al.Prepregnancy restless legs syndrome (Willis-Ekbom disease) is associated with Perinatal depression J Clin Sleep Med 2014;10:527-33.

127. Silva M-RG, Bellotto ML. Nutritional Requirements for Maternal and Newborn Health. Current Women's Health Reviews 2015;11: 41-50.

128. Centers for Disease Control and Prevention. (2011). National Sleep Awareness Week — March 7–13, 2011. MMWR Morb Mortal Wkly Rep, 60 (8): 233.

129. Picchietti DL, Hensley JG, Bainbridge JL, et al. Consensus clinical practice guidelines for the diagnosis and treatment of restless legs syndrome/Willis-Ekbom disease during pregnancy and lactation. Sleep Med Rev 2014;pii: S1087-0792(14)00119-1.

130. Halson SI. Nutrition, sleep and recovery. Eur J Sport Sci 2008;8(2): 119-126.

131 ·Brown WJ, Hayman M, Haakstad LAH, Lamerton T, Mena GP, Green A, et al. Australian guidelines for physical activity in pregnancy and postpartum. Journal of Science and Medicine in Sport. 2022;25(6):511-9. doi:10.1016/j.jsams.2022.03.008.Epub 2022 Mar 16. PMID: 35418334

132 · · Meah VL, Davies GA, Davenport MH. Why can't I exercise during pregnancy? Time to revisit medical 'absolute' and 'relative' contraindications: systematic review of evidence of harm and a call to action. Br J of Sports Med. 2020 Dec;54(23):1395-1404.

**ACTIVE PREGNANCY GUIDE - Physical activity, nutrition, and sleep**

**Funded by:**
Swiss-Portuguese Seed Money CCISP – Conselho Coordenador dos Institutos Superiores Politécnicos (Portugal) – HES-SO – Haute École Specialisée de Suisse Occidentale (Switzerland) collaborative research: ACTIVE PREGNANCY - PROMOTING PHYSICAL EXERCISE AND A HEALTHY LIFESTYLE DURING PREGNANCY AND POSTPARTUM.

**Promotors:**
IPSANTARÉM - Polytechnic Institute of Santarém, Portugal: ESDRM – Sport Sciences School of Rio Maior; ESSS – Health School of Santarém; ESAS – Agrarian School of Santarém
and HES-SO - University of Applied Sciences and Arts Western Switzerland: HESAV - School of Health Sciences, Lausanne

**Principal Researchers:**
Rita Santos Rocha and Jennifer Masset

POLITÉCNICO DE SANTARÉM
ESCOLA SUPERIOR DE DESPORTO DE RIO MAIOR

CONSELHO COORDENADOR DOS INSTITUTOS SUPERIORES POLITECNICOS

HESAV
HAUTE ÉCOLE DE SANTÉ VAUD

Hes·so
Haute Ecole Spécialisée de Suisse occidentale
Fachhochschule Westschweiz
University of Applied Sciences Western Switzerland
