# Supplementary material for: Impact of in-person versus online supervised multicentre multicomponent prenatal exercise programme on maternal physical activity, fitness and healthy lifestyle: the Active Pregnancy trial SPIRIT 2025-based protocol
Source: BMJ Open Sport Exerc Med. 2025 Jul 11;11(3):e002767. doi: 10.1136/bmjsem-2025-002767 (PMC12248212; doi:10.1136/bmjsem-2025-002767)
Supplement: online supplemental file 2 [file bmjsem-11-3-s002.pdf]

Promotion of physical activity and exercise during pregnancy and postpartum

Health professionals guide

Rita Santos Rocha
Anna Szumilewicz
Jennifer Wegrzyk
Mathilde Hyvärinen
Claire de Labrusse
Franziska Schläppy
Maria-Raquel G. Silva
Miguel Ángel Oviedo-Caro

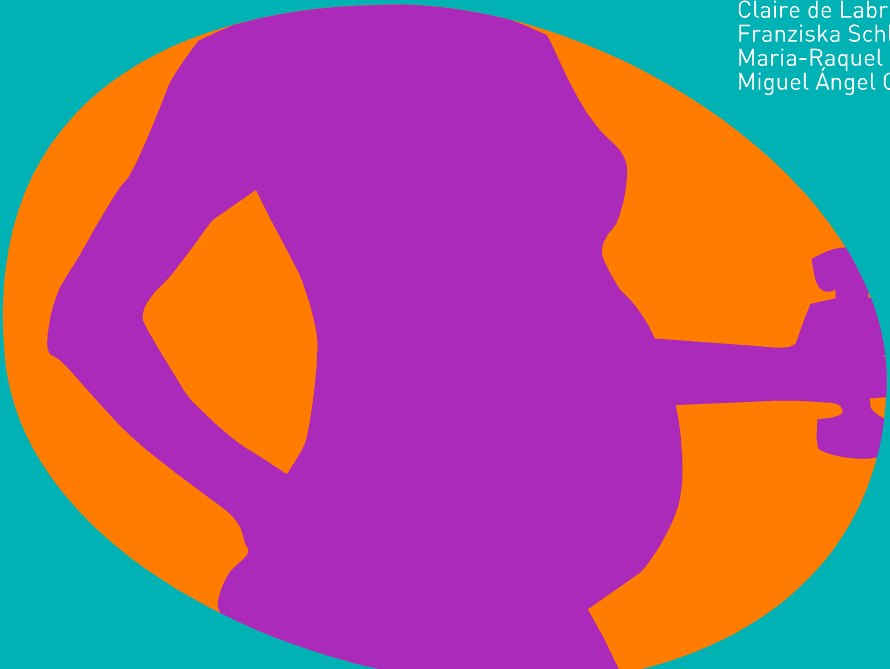

PROMOTION OF PHYSICAL ACTIVITY AND EXERCISE DURING PREGNANCY AND POSTPARTUM. HEALTH PROFESSIONALS GUIDE

Rita Santos-Rocha[1], Anna Szumilewicz[2], Jennifer Wegrzyk[3], Mathilde Hyvärinen[4], Claire De Labrusse[5], Franziska Schläppy[6], Maria-Raquel G. Silva[7], Miguel Ángel Oviedo-Caro[8]

ISBN 978-989-8768-42-1

[1] **R. Santos-Rocha, PhD**

ESDRM-IPSANTARÉM Sport Sciences School of Rio Maior, Polytechnic Institute of Santarém, Rio Maior, Portugal

CIPER Laboratory of Biomechanics and Functional Morphology, Interdisciplinary Centre for the Study of Human Performance, Faculty of Human Kinetics, University of Lisbon, Cruz Quebrada-Dafundo, Portugal

e-mail: ritasantosrocha@esdrm.ipsantarem.pt / ritasantosrocha@gmail.com

[2] **A. Szumilewicz, PhD**

Gdansk University of Physical Education and Sport, Gdansk, Poland

e-mail: anna.szumilewicz@awf.gda.pl / anna.szumilewicz@gmail.com

[3] **J. Wegrzyk, PhD**

HESAV School of Health Sciences, HES-SO University of Applied Sciences and Arts Western Switzerland, Lausanne, Switzerland

e-mail: jennifer.masset@hesav.ch

[4] **M. Hyvärinen, MSc**

HESAV School of Health Sciences, HES-SO University of Applied Sciences and Arts Western Switzerland, Lausanne, Switzerland

e-mail: mathilde.hyvaerinen@hesav.ch

[5] **C. De Labrusse, PhD**

HESAV School of Health Sciences, HES-SO University of Applied Sciences and Arts Western Switzerland, Lausanne, Switzerland

e-mail: clairedelabrusse@hesav.ch

[6] **F. Schläppy, MSc**

HESAV School of Health Sciences, HES-SO University of Applied Sciences and Arts Western Switzerland, Lausanne, Switzerland

e-mail: franziska.schlappy@hesav.ch

[7] **M.-R.G. Silva, PhD**

Faculty of Health Sciences, University Fernando Pessoa, Porto, Portugal

CIAS Research Centre for Anthropology and Health, University of Coimbra, Coimbra, Portugal

CHRC Comprehensive Health Research Centre - Group of Sleep, Chronobiology and Sleep Disorders, Nova Medical School, Nova University of Lisbon, Lisbon, Portugal

e-mail: raquel@ufp.edu.pt

[8] **M.A. Oviedo-Caro, PhD**

Department of Physical Education and Sport, University of Seville, Seville, Spain

e-mail: maovicar@gmail.com

CONTENTS

C

Foreword 5

1. Physical activity during pregnancy – a public health issue 6

2. Definitions regarding physical activity promotion in perinatality 8

3. Role of healthcare providers in promoting physical activity 9

4. Recommendations for physical activity during pregnancy and postpartum 12

5. Behavioral change in physical activity 16

6. How to promote physical activity in perinatality by means of motivational counselling 18

6.1. Inactive women 20

6.2. Partially active and active women 21

6.3. Athletes 22

7. Health screening and contraindications for physical activity in pregnancy 23

7.1. Health screening 23

7.2. Contraindications for physical activity in pregnancy 24

8. Evaluation of physical activity level and setting of objectives 27

9. Exercise testing during pregnancy 29

10. Exercise prescription for pregnant women 33

10.1. Type of exercise 37

10.2. Exercise duration, frequency, and intensity (volume) 39

10.3. Exercise progression and adaptation to body changes 40

First trimester 40

Second trimester 41

Third trimester 41

11. Exercise prescription in special conditions 42

11.1. Gestational diabetes 42

11.2. Excess weight and obesity 43

11.3. Hypertension and preeclampsia 44

11.4. Low back pain 45

11.5. Depression and mental disorders 45

11.6. Urinary incontinence 46

12. Safety issues regarding exercise during pregnancy 48

12.1. Hydration 48

12.2. Falls and injury 49

12.3. Nausea and dizziness 50

12.4. Heat, humidity, and environment 51

12.5. Sportswear and shoes 52

12.6. Hight-intensity or prolonged exercise 52

13. Recommendations to reduce time spent in sedentary behavior 53

14. Exercise prescription for early postpartum women 55

14.1. Exercise in the immediate postpartum period 56

14.2. Benefits of postpartum physical activity and exercise 56

14.3. General physical activity guidelines in postpartum 57

14.4. Pre-exercise assessment in the early postpartum 59

14.5. General exercise prescription components in postpartum 61

14.6. Exercise selection and adaptation in the early postpartum 65

References 68

FOREWORD

Physical Activity and Exercise should be part of an active lifestyle during pregnancy and the postpartum period, as shown by growing evidence on its health benefits for pregnant women and newborns. Currently, there is consensus that maintaining light to vigorous physical activity during an uncomplicated pregnancy has several benefits for the health of the woman and the fetus. The World Health Organization (WHO) recommends that "women who, before pregnancy, habitually engaged in vigorous-intensity aerobic activity or who were physically active, can continue these activities during pregnancy and the postpartum period." [1]. Thus, pregnancy and the postpartum period provide good opportunities for promoting women's health and an active and healthy lifestyle, including proper nutrition and sleep patterns.

Health professionals such as gynecologists, general practitioners, midwives, physiotherapists, nutritionists and psychologists should support women to take an active role via shared decision-making on the management of an active lifestyle during and after pregnancy. All health professionals providing care during pregnancy should be familiar with the international recommendations for physical activity, exercise prescription, contraindications, signs, and symptoms based on which physical activity/exercise should be modified or avoided.

As endorsed by the WHO [1], "some physical activity is better than none". However, appropriate and supervised exercise prescription is needed to tailor effective and safe exercise programs. Exercise prescription in pregnancy is the planning of exercise and fitness-related activities designed to meet the health and fitness goals and motivations of the pregnant woman across the three trimesters, taking into account fitness level and experience with exercise, body adaptations and pregnancy-related symptoms at each stage of pregnancy.

Based on scientific evidence, the purpose of this publication PROMOTION OF PHYSICAL ACTIVITY AND EXERCISE DURING PREGNANCY AND POSTPARTUM. GUIDE FOR HEALTHCARE PROFESSIONALS is to provide healthcare professionals with a basic understanding of the importance of an active and healthy lifestyle during the different stages of pregnancy and cues to promote and tailor physical activity and specific exercise programs in daily life.

1

PHYSICAL ACTIVITY DURING PREGNANCY – A PUBLIC HEALTH ISSUE

Further discussion, and an update of existing guidelines and evidence-based practice are provided in another publication [2].

Physical activity is associated with health benefits during pregnancy, delivery and the postpartum period. In the last three decades, an increasing amount of scientific evidence proves the positive effects of prenatal physical activity on maternal and fetal health, as well as on pregnancy outcomes. Yet, insufficient levels of physical activity are stated in pregnant women worldwide. Physical inactivity during pregnancy is a significant public health[9] issue due to its prevalence and association with adverse pregnancy and birth outcomes, as well as the short- and long-term risk for several chronic diseases for mother and child.

Current research suggests that healthy pregnant women can begin or maintain moderate intensity aerobic exercise programs with no risk of adverse effects on their unborn fetus [3-6]. The role of the health care provider is also to update pregnant women on this knowledge.

Recent systematic reviews show strong evidence on the effectiveness of (moderate to vigorous intensity) physical activity:

[9] Public health encompasses many disciplines that promote health and prevent disease and disability in defined populations.

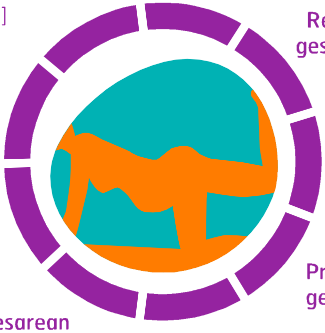

Moreover, moderate evidence suggests positive impact, no negative effect of exercise on Apgar score [24], and no evidence shows an association between leisure-time physical activity or exercise, and an increased risk of preterm birth [24].

Further evidence-based beneficial effects have been stated for: weight retention/weight loss; breastfeeding; musculoskeletal complaints; infant neuronal development and long-term development of Non-Communicable Diseases (NCDs) in the mother and child [5, 6, 8, 15, 21-23].

DEFINITIONS REGARDING PHYSICAL ACTIVITY PROMOTION IN PERINATALITY

2

Physical activity is defined as any bodily movement produced by the contraction of skeletal muscles that results in a substantial increase in caloric requirements over resting energy expenditure [25]. Physical activity can be categorized either by different contexts, such as leisure-time, exercise, sports, occupational, household, and transportation activities, or by intensity, i.e., light (between 1,5 and 2,9 METs - metabolic equivalents[10]), moderate (between 3 and 5.9 METs), and vigorous (6 METs or more) [26, 27]. In contrast, sedentary behavior involves activities of less than 1.5 METs including desk-based office work, driving a car and watching television.

Exercise is a type of physical activity consisting of planned, structured, and repetitive body movement to improve and/or maintain one or more components of physical fitness [25]. Exercise is a subcategory of physical activity. Although energy expenditure is increased during physical activity, it does not necessarily reflect exercise, and should not be confused with fitness [25].

Physical fitness is defined as a set of attributes or characteristics that individuals have or achieve with regards to their ability to perform physical activity [27]. These characteristics are usually classified into health-related and skill-related components of physical fitness. According to the American College of Sport Medicine (ACSM) [27], health-related physical fitness components include cardiorespiratory endurance, body composition, muscular strength and endurance, and flexibility; while skill-related components of physical fitness include agility, coordination, balance, power, reaction time, and speed. According to the definition of the United States Department of Health and HumanServices - USDHHS [28], the components of physical fitness include: cardiorespiratory fitness, musculoskeletal fitness, flexibility, balance, and speed.

Physical inactivity is a behavioral state of not achieving a certain minimum standard of physical activity on a regular basis [26, 27], i.e., failing to meet the WHO recommended level of physical activity. Physical inactivity is the fourth leading cause of death worldwide [29] and considered the strongest public health concern of the 21st century [30].

[10] MET = Multiple of resting metabolic rate, used as a measure of exercise intensity.

3

ROLE OF HEALTHCARE PROVIDERS IN PROMOTING PHYSICAL ACTIVITY

Challenges for pregnant women of practicing physical activity during perinatality are numerous and include lack of knowledge about existing recommendations, unawareness of how to engage in physical activity, social isolation and unavailability of physical activity offers. Despite the above stated scientific evidence, health professionals often lack either knowledge of existing recommendations and pregnancy-related benefits or resources to adequately address the topic [31]. Among health professionals, midwives are ideally placed to promote physical activity during pregnancy consultations as part of a wider network of practitioners [32,33]. After the assessment of potential contraindications for exercising, healthcare providers should provide counseling on an active lifestyle and - if needed and desired - refer pregnant women to an exercise professional (e.g., exercise physiologist, exercise specialist, adapted physical activity specialist, etc.) with a background and experience in pregnancy and/or postpartum physical activity and/or exercise. Therefore, interprofessional collaboration is essential. All professionals working in health promotion should know when, how, and towards whom to guide women with specific needs related to pathologies for additional support [34].

When exercising during pregnancy, women need to feel safe and professionally guided to ensure proper technique, confidence, and appropriate progression of intensity and complexity [35]. The exercise professional should provide regular feedback, positive reinforcement, and behavioral strategies to enhance adherence.

Interprofessional settings (including healthcare providers and exercise experts) can help to reach fitness goals, tailor exercises according to abilities and - most importantly - minimize the risk of injury [26, 27, 36]. The ACSM [37, 38] recommends that physical activity programs should be individualized for each woman based on situation, preferences and motivation experience and current health status. Exercise professionals can notably support aerobic training, strength training, flexibility, balance, pelvic floor muscle training, during pregnancy and postpartum [38-41]. The National Health Services (NHS) guidelines [42] advise pregnant women to make sure that exercise professionals are properly qualified and informed about their pregnancy status.

The Sports Medicine Australia (SMA) guidelines [43] advise pregnant women to ask for a medical doctor's recommendation to consult exercise specialists in view of an individually prescribed exercise program including appropriate types of activities and ways to progress at a safe and steady pace. The Canadian guidelines [44] and the Royal Australian and New Zealand College of Obstetricians and Gynaecologists (RANZCOG) guidelines [45] highlight fitness professionals and exercise physiologists as target users of their evidence-based guidelines in view of maternal, fetal and neonatal health outcomes of prenatal physical activity. The Physical Activity Guidelines for Americans by USDHHS [28] state that physical activity specialists can encourage to attain and maintain regular physical activity by providing advice on adapted activities and ways to progress at a safe and steady pace, even for individuals with chronic conditions. These statements in the official position documents highlight the increasing importance of the exercise professional in promoting and implementing effective and adapted / safe programs.

Depending on the country, exercise specialists are movement professionals with a bachelor or master's degree in sport sciences (i.e., exercise physiologist, adapted physical activity specialist, sport coach) or a specific health profession (i.e., physiotherapists, kinesiologists). Exercise specialists have different ways to promote physical activity in perinatality including: motivational counselling, adapted physical activity and exercise group sessions, and exercise prescription. In 2016, EuropeActive published the role and standards of the Pregnancy and Postnatal Exercise Specialist, based on the European Qualification Framework [46]. The role of the exercise specialist is to encourage exercise participation for beginners and already active women at all stages of pregnancy and during the postpartum period [46] including medical clearance, the assessment of overall physical fitness, the development of adapted exercise programs, feedback on progress, adherence and outcomes to relevant stakeholders. To do so, it is important to understand motivations, facilitators, and barriers for exercising not only for pregnant women in general but on an individual basis [47].

Knowledge acquisition is not always sufficient for inactive women to initiate a behavior. Self-efficacy is crucial to be physically active during pregnancy and can be improved through counselling [48]. Motivational counselling using tools such as the Five A's (Ask, Advise, Assess, Assist, and Arrange) are recommended by the American College of Obstetricians and Gynecologists (ACOG) to promote daily routine in physical activity and to limit sedentary behavior [31]. Education enhances knowledge on physical activity recommendations and health benefits; skills on how, when, and where to practice physical activity, and awareness of health care social support. Moreover, developing accurate and tailored advice

could support women to make informed behavioral decisions concerning physical activity [48].

Group sessions of adapted physical activity could initiate behavior change by promoting positive experiences, providing social support, and self-experiencing training in a safe environment [49]. A one-hour session could consist of a fun warm-up; a main part with strengthening, balance and endurance exercises, and a cool-down with mobility and stretching exercises. Sessions free of charge is recommended in view of health equity [50].

Exercise prescription commonly refers to a specific exercise program designed for a concrete purpose and often developed by an exercise or rehabilitation specialist for the client [26, 27, 39]. An ideal exercise program should meet individual fitness goals according to the components: balance, coordination, gait, agility, and proprioceptive training [27] and take into account the respective stages of life (such as pregnancy) and clinical conditions.

"…The exercise professional should provide regular feedback, positive reinforcement, and behavioral strategies to enhance adherence…"

4

RECOMMENDATIONS FOR PHYSICAL ACTIVITY DURING PREGNANCY AND POSTPARTUM

Tanha et al. [51] showed that the dissemination of official guidelines among pregnant women significantly contributes to increased participation in prenatal exercise. Official guidelines published by national and international obstetrics, gynecology, or sports medicine institutions are a trustworthy and comprehensive source of information in terms of safety and health benefits of exercise during pregnancy and should be fostered by health professionals. They should thus be accessible to all interested parties: pregnant women and their families, obstetric care providers, physiotherapists / kinesiologists and exercise professionals to enable an effective cooperation in the exercise program design.

The fact that the World Health Organization provides specific recommendations on physical activity for pregnant and postpartum women since 2020 [1], highlights the relevance of this topic. An extensive review of recent guidelines is provided elsewhere [41, 52-55]. The recommendations on physical activity during pregnancy and postpartum, published recently, are summarized in Box 1.

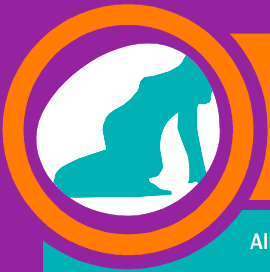

Box 1 SUMMARY OF THE RECENT RECOMMENDATIONS ON PHYSICAL ACTIVITY DURING PREGNANCY AND POSTPARTUM, ADAPTED FROM [53]

All women should be encouraged to participate in aerobic and strength-conditioning exercises as part of a healthy lifestyle during their pregnancy

150 to 300 minutes of intentional physical activity of moderate to vigorous intensity per week

The exercise program should include: aerobic exercise and resistance exercise including pelvic floor muscle training, flexibility, balance, and coordination exercise

Time spent on sedentary activities should be limited

Recommended activities / exercises (adapted if needed) e.g.: Aerobics, dancing, walking, jogging, running, resistance training, swimming, water exercise, cycling, cross-country skiing, Pilates, yoga, balance and posture, exercises preparing for childbirth

Activities / exercises to be avoided: e.g. Scuba diving, horseback riding, downhill skiing, team sports with a high potential for contact, activities with a high potential for falls and trauma

Pregnant women can consult healthcare professionals anytime to know whether or how to adjust their physical activity during pregnancy and the postpartum period

If complications occur, a specialist should be consulted to individualize physical activity, rather than abandoning it

Women who habitually engaged in aerobic and/or vigorous-intensity activities before pregnancy can continue these activities during pregnancy and the postpartum period

Exercise routines can be gradually resumed after pregnancy depending on the mode of delivery, vaginal or cesarean, and the presence or absence of medical or surgical complications

Moderate exercise during lactation does not affect the quantity or composition of breast milk and impact infant growth

Nursing women should consider feeding their infants before exercising in order to avoid exercise discomfort of engorged breast

Recommended types of exercise are specified for the early postpartum period and after full recovery of pelvic and musculoskeletal structures

Return to high impact activities including those with high gravitational load on the pelvic floor should occur gradually, and in consideration of individual recovery processes of pelvic floor and abdominal muscles depending on the mode of delivery

The recently published official recommendations on physical activity during pregnancy and postpartum can be accessed in the following links shown in Box 2.

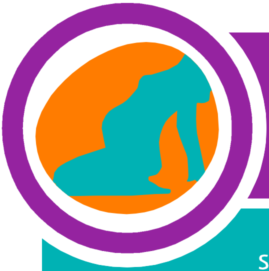

Box 2 LINKS TO RECENT RECOMMENDATIONS ON PHYSICAL ACTIVITY DURING PREGNANCY AND POSTPARTUM, ADAPTED FROM [53]

SOGC - Society of Obstetricians and Gynaecologists of Canada / CSEP - Canadian Society for Exercise Physiology, 2018 [44]: https://bjsm.bmj.com/content/52/21/1339

U.S. DHHS - U.S. Department of Health and Human Services, 2018 [28]: https://health.gov/sites/default/files/201909/Physical_Activity_Guidelines_2nd_edition.pdf

IOC - International Olympic Committee, 2018 [56, 57]: https://bjsm.bmj.com/content/52/17/1080.long | https://bjsm.bmj.com/content/51/21/1516.long

EIM/ACSM - Exercise is Medicine/American College of Sports Medicine, 2019 [38]: https://www.exerciseismedicine.org/assets/page_documents/EIM_Rx%20for%20Health_Pregnancy.pdf

WHO – World Health Organization, 2020 [2]: https://bjsm.bmj.com/content/54/24/1451.long

NHS - National Health Service (United Kingdom), 2020 [42]: https://www.nhs.uk/pregnancy/keeping-well/exercise/

ACOG - American College of Obstetricians and Gynecologists, 2020 [31]: https://www.acog.org/clinical/clinical-guidance/committee-opinion/articles/2020/04/physical-activity-and-exercise-during-pregnancy-and-the-postpartum-period

ACSM - American College of Sports Medicine, 2020 [37]: https://www.acsm.org/docs/default-source/files-for-resource-library/pregnancy-physical-activity.pdf?sfvrsn=12a73853_4

AGDH - Australian Gavernment. Department of Health, 2020 [58]: https://www.health.gov.au/resources/publications/physical-activity-and-exercise-during-pregnancy-guidelines-brochure

SMA - Sport Medicine Australia, 2021 [43]: https://sma.org.au/sma-site-content/uploads/2017/08/SMA-Position-Statement-Exercise-Pregnancy.pdf

RANZCOG – The Royal Australian and New Zealand College of Obstetricians and Gynaecologists, 2021 [45]:

Brazilian Society of Cardiology, 2021 [59]: https://www.ncbi.nlm.nih.gov/pmc/articles/PMC8294738/#S01

5

BEHAVIORAL CHANGE IN PHYSICAL ACTIVITY

Pregnancy is an opportune time for the promotion of healthy lifestyles. Behavioral determinants of health evolve naturally and healthy behaviors are reinforced over unhealthy behaviors, because women are more motivated to do the best for their babies. Women are getting in regular contact with health care professionals and are more likely to adopt a healthy lifestyle if recommended by health professionals [31]. As a special stage of life, pregnancy includes several phases involving specific needs which should be considered through individual adaptation of existing recommendations and guidelines for physical activity promotion during pregnancy. Moreover, pregnancy is a complex phenomenon that considerably differs from one woman to another, and even for one and the same woman having several pregnancies.

Exercise and healthcare professionals should rather guide than control the behavior of pregnant women. To do so, it is important to understand the barriers and facilitators for participation [47], including beliefs, feelings, skills, and knowledge [49]. In summary, three profiles of pregnant women can be distinguished in terms of exercise practice and should be advised differently:

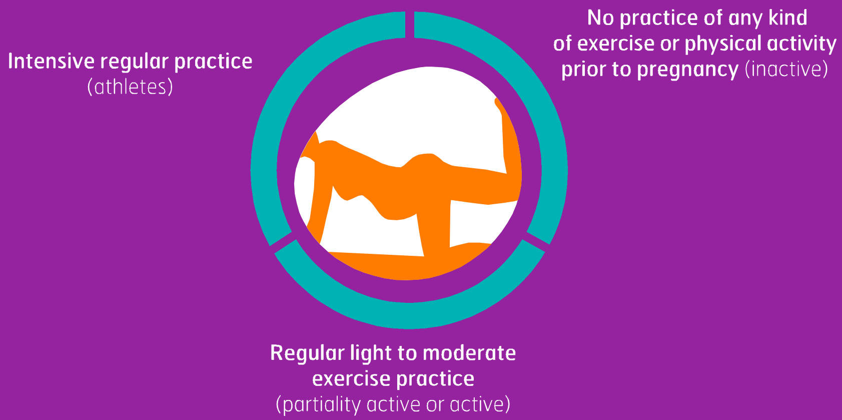

Current research shows that healthy pregnant women can engage in moderate physical activity and maintain moderate to vigorous exercise programs respectively with no risk of adverse effects on their unborn fetus [3-6]. Previously sedentary women can engage in light activity anytime and progressively increase. Previously active women can continue their daily physical activity and exercise routine. Pregnant athletes can also continue their daily physical activity and exercise routine with some adaptations regarding exercise selection and safety.

Commonly observed pregnancy-related symptoms (such as low back pain, tiredness or nauseousness) may interfere with the adoption of an active lifestyle. Information on body changes and health benefits through personalized counselling about physical activity is recommended for each profile of women to overcome barriers. Motivational counselling - as outlined hereafter - is particularly appreciated by women [31].

Physical activity guidelines do not need to be met to benefit from being physically more active.

"...As a special stage of life, pregnancy includes several phases involving specific needs..."

6

HOW TO PROMOTE PHYSICAL ACTIVITY IN PERINATALITY BY MEANS OF MOTIVATIONAL COUNSELLING

Motivational counselling adapts to each woman's experience and knowledge. In accordance with the tool "Five A's (Ask, Advise, Assess, Assist, and Arrange)", the following questions could be addressed:

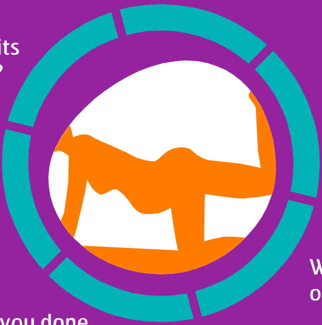

Subsequently, the questions can be extended / specified:

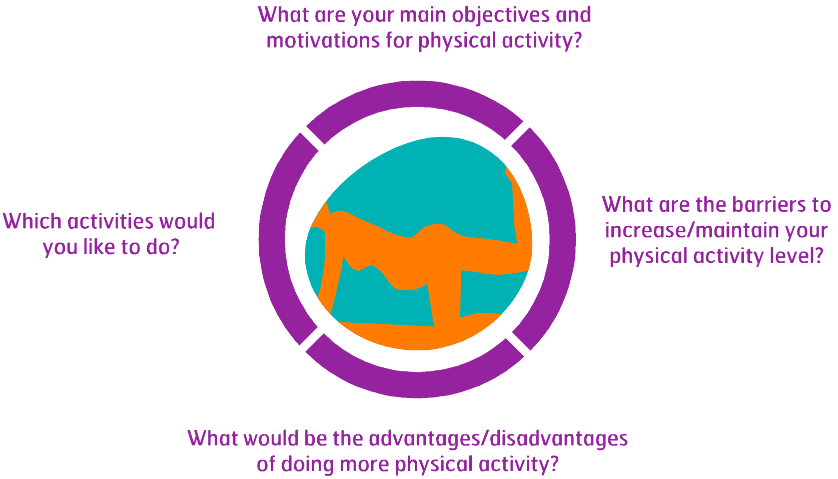

The achievement or non-achievement of physical activity recommendations before and during pregnancy provides a means of situating women in the transtheoretical behavioral change process model by Prochaska et al. [60] as basis for motivational counselling. This model is built on different stages: pre-contemplation (not thinking about changing), contemplation (thinking about changing), preparation (preparing to change/ initiate change), action (making the change), maintenance (sustaining the change over time) and relapse. The pre-contemplation stage is not part of the behavioral change process. The other five stages are related to each other in a circular and non-linear way; the individual may move from one to the other. Relapses are part of the process of behavioral change and can occur when the life trajectory is altered, for example during parenthood.

6.1. Inactive women

Women who identify as inactive before pregnancy usually become more sedentary and less physically active as pregnancy progresses [61, 62]. According to Prochaska et al. [60] misinformation about the consequences of a behavior does not enable a person to overcome the pre-contemplation stage. A woman who is inactive before pregnancy has no interest in changing her behavior during pregnancy because she has never experienced the benefits of physical activity for herself and her child. Providing women information could support them to move to the first stage of the change process (contemplation). Another issue with inactive women is a lack of self-efficacy and skills to perform the behavior (preparation). These competences can be developed through motivational counselling and/or participation in adapted physical activity sessions/ exercise prescription [49].

Doing any physical activity is better than doing none [1]. Inactive woman should start with small amounts of physical activity and gradually increase frequency, intensity and duration over time [1]. Any forms of increasing the volume of physical activity as outlined in Box 3 count. All examples should be recommended to the three profiles of women in perinatality.

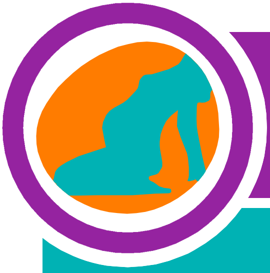

Box 3 FORMS OF INCREASING THE VOLUME OF PHYSICAL ACTIVITY OR EXERCISE THROUGH OCCUPATIONAL ACTIVITIES, ACTIVE COMMUTING, AND DAILY ACTIVITIES, ADAPTED FROM [2]

Starting the day with some stretching
**Increasing walking time** (i.e. walking the dog, parking further away from the office or residence)
Using a pedometer and aiming for 10,000 steps per day (minimum 5.000 steps/day)
Walking or biking for active commuting
Engaging in walking groups with other mothers and babies, or with family and friends

Taking stairs instead of the elevator as often as possible
Doing housing chores, gardening, etc.
Avoiding standing in the same position for long periods of time (stand up each hour)
**Limiting the seating time** (i.e., using a standing desk)
Doing stretching breaks during seated activities or desk exercises, dancing with music at home
**Reducing sedentary activities** (e.g., television watching, computer use, sitting in a car or at a desk)
**Getting all family involved** (i.e., playing with the baby or other children)
Using available mobile applications that encourage physical activity
**Performing short bouts of exercise at home** (e.g., by following YouTube videos [63, 64]), such as:<br>Active at home / Active outdoors YouTube Chanel:<br>https://www.youtube.com/channel/UCEUWdoBeh5rgfM0kZOn9Xtg/videos<br>Active Pregnancy YouTube Chanel:<br>https://www.youtube.com/channel/UC0VyookwcOmcQ5T7OimtoNA/playlists

6.2. Partially active and active women

Women who were partially active/active before pregnancy and who became less active (preparation) or even inactive (contemplation) during pregnancy are also unaware of the benefits of physical activity during pregnancy and postpartum, the recommendations and how to implement them. Giving them more concrete information on "what", "how" and "where" could support them to achieve the recommendations. Women who already have a physical activity routine before pregnancy could, with this information given, move quickly through the stages of the behavioral change process (contemplation, preparation, and action) to maintain physical activity. Skills, self-efficacy and perception of control can also be successfully addressed through motivational counselling and/or participation in adapted physical activity sessions/ exercise prescription in partially and/or physically active women [49].

6.3. Athletes

Regarding the pregnant athlete, vigorous intensity exercise appears to be safe in healthy pregnancies [31] unless extreme sports are practiced [65]. Elite athletes who wish to become pregnant, should discuss specific issues with their medical team [44, 56]. According to the Australian Sports Commission [65], recreational and competitive athletes may train safely at high intensities and volumes throughout pregnancy while being under obstetric supervision. Due to the risk of trauma, athletes performing contact sports may be advised to switch to fitness-oriented exercises. The key message of the International Olympic Committee (IOC) [56] is that elite athletes with an uncomplicated pregnancy should be reassured that they can continue exercising, although some adjustments in intensity and activity may be required.

"...Elite athletes who wish to become pregnant, should discuss specific issues with their medical team..."

HEALTH SCREENING AND CONTRAINDICATIONS FOR PHYSICAL ACTIVITY IN PREGNANCY

7

7.1. Health screening

Health screening, i.e., the review of overall health and medical and obstetric risks by a healthcare professional should precede an exercise program during pregnancy [1, 28, 31, 37, 42-45, 56, 58, 59] to confirm the absence of contraindication to physical activity.

The ACOG's Antepartum Records and the Postpartum Care forms can assist health care providers. These forms are available at the ACOG website upon registration:

https://www.acog.org/clinical-information/obstetric-patient-record-forms

The GET ACTIVE QUESTIONNAIRE FOR PREGNANCY by CSEP [68] replaced the Physical Activity Readiness Medical Examination (PARmed-X) for Pregnancy, after the publication of the Canadian guidelines [44]. This 2-page guideline for health screening facilitates the communication between healthcare professionals, exercise specialists and pregnant women. It includes guidance on exercise prescription, healthy lifestyle during pregnancy, and exercise safety. The questionnaire is available online in English and French:

https://csep.ca/wp-content/uploads/2021/05/GAQ_P_English.pdf

The GET ACTIVE QUESTIONNAIRE FOR PREGNANCY by CSEP [68] and companion HEALTH CARE PROVIDER CONSULTATION FORM FOR PRENATAL PHYSICAL ACTIVITY by CSEP [69] helps healthcare professionals to engage in a meaningful conversation about health benefits of physical activity during pregnancy. The questionnaire is available online in English and French:

https://csep.ca/wp-content/uploads/2021/05/GAQ_P_HCP_English.pdf

Another validated and comprehensive inventory to objectively monitor pregnancy-related symptoms is available from Foxcroft et al. [70]: https://bmcpregnancychildbirth.biomedcentral.com/articles/10.1186/1471-2393-13-3

These forms may be used in combination with other preliminary screening tools, such as the latest version of the Physical Activity Readiness Questionnaire for Everyone (PAR-Q+) available at the official website [71]. This questionnaire can be filled out by medical doctors, healthcare practitioners such as midwives, or qualified exercise specialists in order to support the decision of whether further advice should be sought before engaging in a fitness appraisal. The questionnaire is available online: http://eparmedx.com/

7.2. Contraindications for physical activity in pregnancy

After medical clearance, pregnant women and health professionals should be familiar with the absolute and relative contraindications to exercise during pregnancy, as well as with the signs and symptoms to cease exercise and to seek medical attention(summarized in Box 4). The relative and absolute contraindications for exercising during pregnancy are summarized in Box 5 and Box 6, respectively.

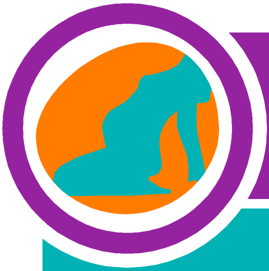

Box 4 **SIGNS AND SYMPTOMS TO CEASE EXERCISE AND SEEK MEDICAL ATTENTION, ADAPTED FROM** [53]

Feeling faint or headache [43, 45]
Chest pain [31, 38, 43-45]
Lower back, pelvic or abdominal pain (potentially indicating pre-term labor) [43, 45]
Calf pain or swelling [38, 43, 45]
Sudden swelling of the ankles, hands or face [45]
Dizziness or presyncope or faintness that does not go away with rest [31, 38, 43-45]
Shortness of breath that does not go away with rest / dyspnea before exertion [31, 38, 43, 44]
Unexplained / excessive shortness of breath [43, 45]
Excessive fatigue [43]

Muscle weakness [45]

Regular painful uterine contractions [31, 38, 43-45]

Decreased fetal movement [43, 45]

Bleeding or amniotic fluid coming from vaginal (indicating rupture of the membranes) [31, 38, 43-45]

Box 5 RELATIVE CONTRAINDICATIONS FOR EXERCISE DURING PREGNANCY, ADAPTED FROM[53]

History of fetal growth restriction [56, 58]

Miscarriage / recurrent pregnancy loss [44, 56]

History of premature birth or labor [44, 56, 58]

Cervical enlargement [56]

Persistent vaginal bleeding in the second or third trimesters [58]

Symptomatic anemia [44]

Poorly controlled seizure disorder [56]

Multiple gestation (triplets or higher) after the 28th week [44, 58]

Unevaluated maternal cardiac arrhythmia [56]

Gestational hypertension [44, 58]

Mild/moderate cardiovascular disease [44, 58]

Chronic bronchitis or other respiratory disorders [44, 56, 58]

Poorly controlled type I diabetes [56, 58]

Extreme underweight [56]

Malnutrition, eating disorder [44]

Orthopedic limitations [56]

Other significant medical conditions [44]

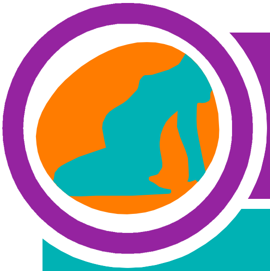

Box 6 ABSOLUTE CONTRAINDICATIONS FOR EXERCISE DURING PREGNANCY, ADAPTED FROM[53]

- Hemodynamically significant heart disease / other serious cardiovascular disorder [44, 56]
- Incompetent cervix / cerclage [44, 56, 58]
- Intrauterine growth restriction in current pregnancy [44, 56]
- Multiple gestation at risk of premature labor [44, 56]
- Other serious systemic disorders [44, 58]
- Placenta previa after 26/28 weeks' gestation [44, 56, 58]
- Pre-eclampsia / pregnancy-induced hypertension [44, 56, 58]
- Premature labor during the current pregnancy [44, 56]
- Restrictive lung disease / respiratory disorder [44, 56]
- Ruptured membranes [44, 56, 58]
- Severe anemia [56]
- Uncontrolled hypertension [44, 56]
- Uncontrolled thyroid disease [44, 58]
- Uncontrolled type I diabetes [44]
- Unexplained persistent vaginal bleeding / persistent second or third trimester bleeding [44, 56]

Any of these signs or symptoms, should induce participants to cease or limit physical activity in order to avoid the risk of future complications. In this case, it is of utmost importance to interact with the exercise professional to adapt daily routine and/or exercise program.

EVALUATION OF PHYSICAL ACTIVITY LEVEL AND SETTING OF OBJECTIVES

8

A healthy pregnant woman without complications may decide either to engage in a supervised or unsupervised physical activity / exercise program that is - ideally - adapted (e.g., through realistic objectives) and motivating (e.g., objective measures of volume). No tests or monitoring are needed. Wearable technology (i.e., pedometers, accelerometers in combination with smartphone apps) and questionnaires, are basic and affordable equipment to monitor physical activity [72].

Accelerometer-based devices allow for objective, reliable and valid measurement of physical activity and show good correlation with indirect calorimetry in pregnant women [73]. With regards to sensor placement, wrist location shows higher compliance than hip location among pregnant women due to anatomical changes of the latter during pregnancy [74]. **Pedometers** allow for step-count only and are less accurate and reliable than accelerometers. They provide an inexpensive tool to objectively measure walking in large-scale studies [75] and are integrated in smartphones.

Questionnaires can be used to recall physical activity pattern and volume, complementing the information provided by wearables. The worldwide reliable and validated **7-day PAR - 7-day Physical Activity Recall** interview [76] is available from the website: https://drjimsallis.org/measure_7daypar.html

The PPAQ - Pregnancy Physical Activity Questionnaire developed by Chasan-Taber et al. in 2004 [77], is a widely used tool for the assessment and measurement of physical activity levels amongst pregnant women. PPAQ is a self-administered questionnaire which assesses sedentary, light, moderate, and vigorous activities regarding household/caregiving, occupational, and sports/exercise activities respectively. Pregnant women are asked to select the category that best reflect the amount of time spent in 32 activities and in inactivity for their current trimester of pregnancy. At the end of the questionnaire, At the end of the questionnaire, an open-ended section allows to add activities that are not listed. Link to the 4-page form PPAQ available at:
https://journals.lww.com/acsm-msse/Fulltext/2004/10000/Development_and_Validation_of_a_Pregnancy_Physical.14.aspx

QUESTIONS REGARDING THE EXERCISE PRESCRIPTION PLAN ARE THE FOLLOWING:

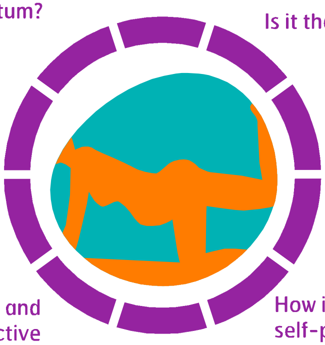

EXERCISE TESTING DURING PREGNANCY

9

Extensive exercise testing during pregnancy is usually only performed for medical reasons or for research purposes [27] unless desired by the pregnant woman. Special care should be taken to ensure that the pregnant woman feels comfortable and there are no risk of falls and injuries. Exercise testing should not be invasive during pregnancy. Instead, it should increase motivation, objectively evaluate the effects of training, and support exercise prescription through the assessment of baseline fitness level, as according to good practice based on the RANZCOG guidelines [45]. In a clinical and research setting, cardiopulmonary exercise testing during pregnancy is valuable in identifying underlying cardiopulmonary conditions, stratifying the risk of adverse pregnancy outcomes, as well as establishing exercise tolerance/limitations, as underlined by Wowdzia and Davenport [78].

Several maximal and submaximal tests exist in practice to evaluate physical fitness components (i.e., cardiorespiratory endurance, body composition, muscular strength and endurance, and flexibility), as well as skill-related components of physical fitness (i.e., agility, coordination, balance, power, reaction time, and speed) [27]. Most tests have been developed for a healthy adult population and allow for an objective evaluation of fitness status and effectiveness of an exercise intervention.

According to the ACSM [27], maximal exercise testing should not be performed on pregnant women unless medically necessary, and under medical supervision. Because maximal exercise testing is rarely performed in pregnant women, Mottola et al. [79] developed and validated heart rate (HR) ranges that correspond to moderate intensity exercise for low-risk pregnant women based on age and body mass index (BMI) while taking into account fitness levels. Those HR ranges are provided in the Canadian [44] and RANZCOG [45] guidelines.

Regarding maximal heart rate, the estimation equations by Gellish et al. [80] are more accurate than other formulas, and were estimated for men and women participants in an adult fitness program with a broad range of age and fitness levels, as follows:

(eq. 1) $$HRmax = 207 - (0.7 \times age)$$

(eq. 2) $$HRmax = 192 - (0.007 \times age^2)$$

Although the nonlinear predictor model (eq. 2) was slightly more accurate than the linear equation (eq. 1), the authors suggest that the linear model is easier to use.

After estimating HRmax and monitoring heart rate at rest (probably with great variability as pregnancy progresses), the exercise intensity can be estimated using the heart rate reserve (HRR) equation [27]:

(eq. 3) **HRR method (training) = [(HRmax – HRrest) x intensity in percent] + HRrest (bpm)**

Submaximal exercise testing is more appropriate for pregnant women [27] in order to estimate the maximum rate of oxygen utilization of muscles during exercise (VO2max). Treadmill walking and upright leg cycling are the most common and convenient testing modalities during pregnancy, since the injury risk is low, physiological monitoring easy (not much vertical movement), and the exercises based on basic movements [81, 82]. Another exercise testing option is a field test consisting of walking over a predetermined time or distance, such as the 6-min walk test. Those tests are easy to administer and require little equipment. The 6-min walk test (6MWT) assesses the distance for a 6 min time interval [26, 27, 39]. This test is easy to administer as it requires little equipment. It is safe in pregnant women, and references exist for resting HR and distance walked [83].

There are no specific tests for the assessment of musculoskeletal function, i.e., muscular strength and resistance, and flexibility, although these components of physical fitness are addressed in the recommended guidelines for exercise during pregnancy. Similarly, no testing exists for skill-related fitness components, i.e., agility, coordination, and balance. However, the main purpose of a prenatal exercise program is to promote maternal-fetal health, rather than to maximize physical performance [84]. Fitness testing protocols might be useful in determining the effects of training, and to increase motivation.

O'Connor et al. [84] found that fatigue during pregnancy is attenuated by adopting low-to-moderate intensity resistance training. However, to our knowledge, the safety and validity of maximal muscle strength assessment for pregnant women is not assessed in literature. Further details on musculoskeletal health adaptations during pregnancy can be found in Fitzgerald and Segal [85].

The **IFIS - International FItness Scale** for self-reported fitness is a simple-to-use tool with demonstrated validity and reliability [86]. This scale is available in several languages at the project' website:

http://www.helenastudy.com/ifis.php

The findings of Romero-Gallardo et al. [87] suggest that IFIS might be a useful tool for identifying pregnant women with low or very low physical fitness and with low health-related quality of life. The findings of Henström et al. [88] suggest that IFIS could be useful to stratify pregnant women in appropriate fitness levels on a population-based level where objective measurement is not possible.

Moreover, body composition is a health-related component of physical fitness. Monitoring weight gain and nutritional status is insightful during pregnancy, especially for those women with excessive weight or obesity status. In these cases, the assessment of body composition, body circumferences, body fat distribution markers and other body indexes can be used [89]. Normal fetal growth depends on adequate maternal energy stores for which guidelines exist. Excessive weight gain is undesirable; however, guilt or blame in pregnant women with regarding their eating habits need to be avoided. The exercise professional can provide general advice regarding healthy nutrition during pregnancy. Dietary analysis and tailored nutrition plans should be conducted by a qualified nutritionist. Further development on this topic can be found elsewhere [90, 91].

"...Treadmill walking and upright leg cycling are the most common and convenient testing modalities during pregnancy..."

10 EXERCISE PRESCRIPTION FOR PREGNANT WOMEN

Exercise prescription and monitoring during pregnancy require expertise in the fields of obstetrics and exercise physiology. In general, an exercise prescription is made for pregnant women with specific conditions or for athletes. Pregnant women fall into the category of apparently healthy adults, although they are considered a special population [27]. Thus, the general guidelines of ACSM regarding the "FITT-VP principle" apply to pregnant women, with some modifications [27, 39]. This principle is based on the following elements that address one or more physical fitness components:

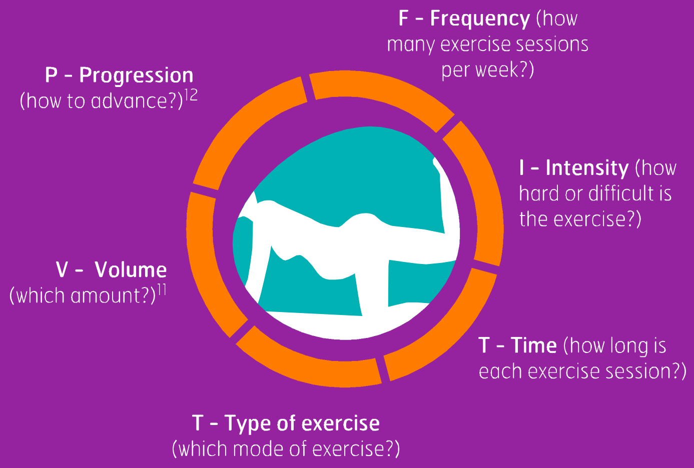

[11] Usually, the volume is considered to be the product of intensity, frequency and duration of the exercise sessions.

[12] With pregnant clients, the "progression" is assumed as the adaptation of exercise to each trimester of pregnancy, rather than focused on intensity and complexity, taking into account the physiological adaptations to pregnancy.

Box 7 EXERCISE PRESCRIPTION FOR PREGNANT WOMEN

Type	Intensity	Duration	Frequency	Progression / Adaptation
Aerobic exercise				
Exercises that activate large muscle groups in a rhythmic and continuous fashion<br><br>A variety of weight- and non-weight-bearing activities are well tolerated during pregnancy<br><br>Aerobic exercises can be categorized by intensity and skill demands	Moderate intensity exercise (3-5.9 METs; RPE = 12-13; 40%-60% VO2reserve)<br><br>Vigorous intensity exercise (>6 METs; RPE = 14-17) for women who were highly active prior to pregnancy or for those who progress to higher fitness levels during pregnancy<br><br>So far, there is little evidence on the influence of exercise of high intensity (RPE > 17) on the course of pregnancy.	30 min / day of accumulated moderate intensity exercise to total at least 150 min / week<br>or<br>75 min / week of vigorous intensity<br>or<br>a mix between moderate and vigorous intensity<br><br>Previously inactive women should progress from 15 to 30 min / day	Previous sedentary: up to 3 days / week<br>Previous active: 3-5 days / week to most days of the week	Avoid activities with risk of fall and trauma<br><br>Activities that require jumping movements and quick changes in direction which can stress joints should be done with caution to - minimize the risk of joint injury

Resistance exercise				
A variety of machines, free weights, and body weight exercises are well tolerated during pregnancy	Intensity that permits multiple submaximal repetitions (i.e., 8-10 or 12-15 repetitions) to be performed to the point of moderate fatigue (40%-60% of estimated one repetition maximum)	1-2 sets for beginners<br>2-3 sets for intermediate and advanced<br>Target major muscles groups<br>A basic program includes 8-10 different exercises	2-3 nonconsecutive days / week	Modifying the position of the exercise to instead be performed on one's side, sitting or standing is a safe alternative<br>Avoid performing the Valsalva maneuver during exercise<br>Heavy-resistance weight lifting and intense repetitive isometric exercises should be performed with caution until more data is available
**Flexibility exercise**				
A series of active or passive static and dynamic flexibility exercises for each muscle-tendon unit	Stretch to the point of feeling tightness or slight discomfort	Hold static stretch for 10-30 s (up to 60 s)<br>2-4 repetitions of each exercise	At least 2-3 up to 7 days / week	Avoid excessive joint stress

Neuromotor exercise				
Exercises involving motor skill, e.g., balance, agility, coordination, gait), proprioceptive training, and multifaceted activities (e.g., Pilates, Yoga, tai chi)	Intensity in balance training refers to the degree of difficulty of the postures, movements, or routines practiced<br><br>An effective intensity (and volume) of neuromotor exercise has not been determined	20-30 to 60 min / day	At least 1-2 up to 7 days / week<br><br>Can be included in daily activities	Avoid positions that are uncomfortable or likely to result in loss of balance and falling
**Pelvic floor training**				
Complex training for pelvic-floor muscles should be focused both on their contraction and relaxation<br><br>Various devices can be used to increase the effectiveness and attractiveness of exercise (e.g. vaginal cones)	An effective intensity (and volume) of pelvic floor exercise has not been determined	10-20 min / day	1-7 days / week<br><br>Should be incorporated in any prenatal exercise program	Can be done anywhere, anytime, everyday<br><br>Ensure proper technique: on the expiration contraction of perineal muscles and then transversal muscles<br><br>Different exercises should be performed to improve pelvic floor muscle speed, strength, endurance and muscular coordination, and engaging both fast and slow twitch muscle fibers

RPE = rating of perceived exertion (6-20 scale); METs = metabolic equivalents; VO2reserve= oxygen uptake reserve (VO2max – VO2rest)

10.1. Type of exercise

Plenty of physical activities can be performed alone or in group, indoor or outdoor, with or without equipment. Pregnant women should select the type of physical activity or exercise program according to their motivation and ability.

Among the recommended types of exercise supported by the guidelines for physical activity during pregnancy and postpartum is walking, jogging, indoor cycling, cross-country skiing, swimming, water exercise, low-impact aerobics, step, dancing, Pilates, yoga, flexibility, balance, posture and functional, pelvic floor muscles training, and resistance training [53]. Other safe activities (for pregnant women who participated in these activities regularly before pregnancy) are running, outdoor cycling, strength training and racquet sports, upon consultation with an obstetric care provider [31, 45]. Further explanation of each type of exercise can be found in Szumilewicz and Santos-Rocha [40]. Further examples of exercises and sessions tailored to pregnant and postpartum women can be found in the ACTIVE PREGNANCY YouTube channel [64]:

https://www.youtube.com/channel/UC0VyookwcOmcQ5T7OimtoNA/playlists

A comprehensive exercise program during pregnancy may include several of the recommended types of exercise and should address all physical fitness components (i.e., aerobic, resistance training, and flexibility), as well as neuromotor exercises, pelvic floor training, and preparation for birth exercises [39, 40, 93]. Each exercise session could consist of at least 30 minutes of aerobic exercise, strength and flexibility training (including posture and functional exercise), neuromotor exercise (especially, balance and coordination), and pelvic floor muscle training. For example, a step exercise session may combine aerobic, lower limb resistance, and neuromotor training, while a Pilates exercise session may combine upper, core and lower limb resistance, posture, flexibility, and neuromotor training.

A typical exercise session should be organized as follows [27, 39, 40]

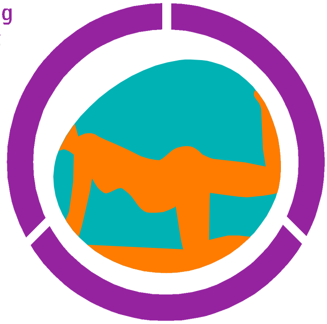

Cool-down period including breathing exercises, light stretching, mobility exercises, pelvic floor training.

5-10 min warm-up period, including, walking, pelvic floor training, light stretching, or movements performed during the main part of the session. Some fun exercises in groups could be done to promotion social inclusion.

The main part should include endurance, strengthening and balance exercises. Each exercise should be presented with variations to let the women choose the best fit.

Regarding exercise selection and adaptation [40], exercise professionals must be aware of the morphological [89], physiological [93, 94], musculoskeletal [85, 95] and biomechanical [96] changes that occur during pregnancy, such as increased ligament laxity, weight gain, increased fatigue, change in the center of gravity, carpal tunnel syndrome and vena cava syndrome that will affect the response to exercise. Moreover, the typical signs and symptoms associated to each trimester of pregnancy, the motivations and objectives, the safety considerations, the fitness level, and the level of experience of the pregnant women, will also impact on exercise selection and adaptation [40]. For instance, indoor or outdoor cycling, aerobic and step exercise can be performed at different intensity and complexity levels. For a pregnant woman who has never practiced these activities, the entire learning process must be kept in mind so as not to jeopardize her safety in the event of falls or collisions.

The USDHHS [28], Canadian [44], AGDH [58], ACOG [31], SMA [43], and EiM [38] guidelines also list non recommended physical activities, including contact sports, activities with high risk of falling, activities at high altitude (when not normally living at high altitude), scuba diving, skydiving, downhill skiing, water skiing, activities in excessive heat (e.g., hot Pilates, and hot yoga). Further explanation can be found in Szumilewicz et al. [53].

10.2. Exercise duration, frequency and intensity (volume)

Exercise duration is prescribed as the amount of time physical activity is performed, i.e., time per session, per day, and per week [27]. Frequency is prescribed as sessions per day and as days per week. Exercise duration typically ranges from 20 to 60 min. Most guidelines for exercise during pregnancy [53] suggest 30 minutes of daily exercise, five to seven days per week. Previously inactive or not regularly exercising women should begin with 10 to 20 minutes of continuous low-intensity exercise three times per week, gradually increasing the intensity, frequency, and duration. If an inactive woman would like to participate in a one-hour group session, the healthcare professional should encourage her. The role of the healthcare professional is to provide sufficient recovery and variation of exercises tailored to the fitness level of participant.

Health professionals can empower women using two practical methods to monitor intensity in practice [26, 27, 43-45]

Perceived exertion (overall sensation of effort): for moderate exercise, ratings of perceived exertion should be 13 to 14 (somewhat hard) on a Borg Rating of Perceived Exertion scale, where 6 represents no exertion and 20 represents maximal exertion.

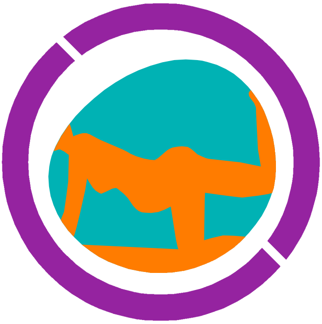

The "talk test": the individual should be able to speak comfortably and in complete sentences reflecting moderate exercise intensity. In contrast, vigorous exercise is associated with substantial increases in breathing, thus an inability to carry on a conversation easily, and perspiration.

Exercise volume is the product of frequency, intensity, and exercise duration. Usually, the exercise volume is used to estimate the gross **energy expenditure** in metabolic equivalents (in MET-min/week or in kcal/week) with respect to body composition and weight management outcomes [26, 27]. Another form of estimating the exercise volume is via **steps per day** using pedometers. The goal of 10,000 steps / day is often cited regarding health benefits, but a pedometer step count of at least 5,400-7,900 steps / day can already meet recommended exercise targets. This step count volume is approximately equal to 1,000 kcal / week or 150 min / week of moderate-intensity physical activity [27].

10.3. Exercise progression and adaptation to body changes

Exercise progression may vary at different time points during pregnancy [39, 40], thus exercise routines should remain flexible and in accordance with the physiological and biomechanical adaptations occurring over the time course of pregnancy [97].

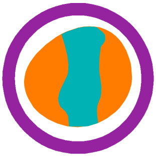

First trimester

The right amount of exercise for a pregnant woman will depend on how active she was before pregnancy. Most women don't need to modify their activities too much during the first trimester of pregnancy. Usually, it is best to favor low impact exercises, e.g., walking, Nordic walking, low-impact aerobics and step, yoga, Pilates, resistance training, indoor cycling, swimming, and water aerobics. Slightly more vigorous exercises may also be appropriate in the first trimester, in case the woman is used to them, e.g., running, jogging, cross-country skiing, outdoor cycling, and moderate weightlifting, etc. Pelvic floor training should be advised from the first trimester.

In the first trimester, most pregnant women experience symptoms, such as fatigue mood changes, nausea, vomiting, breast tenderness, dyspepsia, frequent urination, and constipation, which can limit daily and physical activities. Symptoms and discomfort may prevent some women from practicing physical activity or exercise, and may require adaptations of the types of exercise selected [39, 40].

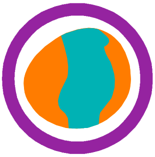

Second trimester

During the second trimester, discomfort of the first trimester is usually gone and physical limitations of the third trimester have not yet appeared. At this stage, the uterine volume increases and the inferior vena cava syndrome can develop. This results in a reduction of venous return through said vessel, due to the pressure exerted by the pregnant uterus in supine position. Some women may therefore feel discomfort or dizziness while lying on their back. In practice, many women reports sleeping while lying on back. Nevertheless, some guidelines recommend avoiding this position after the first trimester of pregnancy [1, 42, 44].

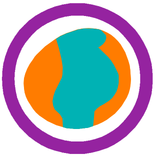

Third trimester

During the third trimester, the increased volume of the gravid uterus as well as the weight gain of the pregnant woman compromises the volume at the abdominal and pulmonary level. Consequently, pregnant women tend to decrease the intensity and duration of their physical activity. It is therefore recommended to start or maintain an adequate amount of physical activity particularly in the third trimester. Activities in the aquatic environment are adequate (weightlessness, mobilizing joints with passive resistance, the appearance of inferior vena cava syndrome is attenuated). The AGDH guidelines [58] recommend that after 28 weeks, exercises should not be performed lying flat on back, but in an upper body tilt of 45-degree angle or while lying on the side. Moreover, decreased balance and coordination can lead to falls. Balance and coordination exercises are recommended at each stage of pregnancy. Hormonal and biomechanical adaptations may be associated with joint and low back pain that could be minimized by strengthening abdominal and back muscles [31]. Pelvic floor muscles training should be maintained. Walking or Nordic walking with good posture and at varying paces can be performed autonomously any time. During the third trimester, women should be advised to prepare for birth program [97-100].

EXERCISE PRESCRIPTION IN SPECIAL CONDITIONS

11

Exercise can be a recreational or competitive component but also serve as an adjunct treatment for several disorders, such as gestational diabetes, excessive weight gain and obesity, low back pain, and antenatal depression and prevention of hypertension and preeclampsia [5, 101]. Women suffering from these conditions face substantial barriers to participate in exercise and require support to initiate and adhere to physical activity. Under medical supervision and in interprofessional collaboration, health professionals do not only need to select appropriate exercise interventions, but also behavioral strategies as described in the chapter of motivational counseling.

11.1. Gestational diabetes

A healthy pregnancy can be associated with resistance to insulin on glucose uptake and utilization [102]. In 1-14% of pregnant women this condition develops into gestational diabetes mellitus (GDM) [103]. Gestational diabetes is the most common metabolic disorder in pregnancy and its prevalence is nowadays increasing because there is a higher number of pregnant women with a body mass index (BMI) or weight gain level in the range of overweight or obesity, and also because childbearing age is increasing [103].

GDM is associated with a wide range of adverse health consequences for women and their infants in the short and long term, including an increased risk of macrosomia, birth complications, and maternal diabetes after pregnancy. It may also increase the risk of obesity and type 2 diabetes in offspring later in life [104, 105]. There is growing evidence that exercise and physical activity can control this condition while being easy to be carried out, effective and with minimum costs [106].

Observational studies strongly support exercise and physical activity as a tool that may control glycemia levels in pregnancy and reduce the risk of gestational diabetes. In the last few years various studies showed the following protective functions of exercise and physical activity:

A significant reduction of developing a gestational diabetes of 28% [105]

Increasing postprandial blood glucose control, and decreasing the rapid passage of blood into the bloodstream [107]

Reduced risks of GDM and cesarean section in combination with diet interventions [108]

To achieve at least a 25% reduction in the odds of developing GDM (and also gestational hypertension and pre-eclampsia), pregnant women need to accumulate at least 600 MET-min/week of moderate-intensity exercise (i.e., approximately 140 min) [109].

Exercise prescription is the same for pregnant women with and without gestational diabetes. However, pregnant women who need glucose-lowering drugs for metabolic control of glycemia should be closely monitored, since exercise may misadjust the prescribed pharmacological regimen.

11.2. Excess weight and obesity

Gestational weight gain (GWG) has often been identified as critical for maternal and fetal health [110]. The ACOG reaffirmed in 2017 that "in pregnancy, physical inactivity and excessive weight gain have been recognized as independent risk factors for maternal obesity and related pregnancy complications, including gestational diabetes mellitus" (p. 136). The ACOG advice that obese pregnant women should be encouraged to engage in a healthy lifestyle including physical activity and judicious diets [111]. This special population should start with low-intensity, short periods of exercise and gradual increase of exercise volume [31, 44]. In the last few years various studies showed:

Exercise in obese pregnant women reduces the odds of excessive weight gain [110, 112, 113]

Supervised physical activity combined with diet programs were most effective in managing weight among overweight and obese pregnant and postpartum women [112], lower the odds of a caesarean section [113] and prevent postpartum weight retention [10]

11.3. Hypertension and preeclamsia

Hypertension[14] in pregnancy may result in adverse perinatal outcomes for mother and fetus / newborn. Preeclampsia in pregnancy and postpartum is due to an abnormal development of uterine spiral arteries. Despite scientific advances in the understanding of risk factors for preeclampsia, and preventive measures, the condition remains the second most prevalent cause of global maternal mortality, reaching 14% [114].

At present, most research in preeclampsia focuses on improving the development of uterine spiral arteries, determining factors related to genetic predisposition, and improving the low immunological response of most pregnant women that develop this pathology [115]. Exercise plays a key role in view of increasing placental vascularity and stimulating the immune system [116]. In the last few years various studies showed:

To achieve at least a 25% reduction in the odds of developing gestational hypertension and pre-eclampsia (and also GDM), pregnant women need to accumulate at least 600 MET-min/week of moderate-intensity exercise (i.e., approximately 140 min) [10]

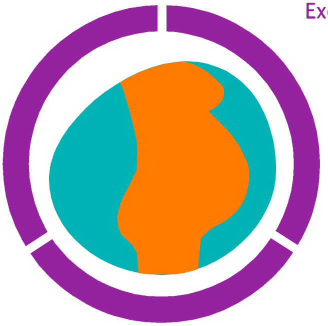

Exercise is safe and beneficial in pregnancies involving hypertension (therapy) [10, 117, 118]

Aerobic exercise for about 30-60 min, two to seven times per week during pregnancy, is associated with a significantly reduced risk of gestational hypertension as compared to sedentary behavior. However, the recommended exercise intensity for these conditions remains unclear [9]

[14] The ACSM [26, 27] defines 'hypertension' as: having a resting systolic blood pressure (BP) of 130 mmHg or greater; having a resting diastolic BP of 80 mmHg or greater; taking antihypertensive medication; being told by a physician or health professional on at least two occasions that one has high BP; or any combination of these criteria.

11.4. Low back pain

The majority of pregnant women experience low back pain that interferes with daily routine and worsens as pregnancy progresses. In the last few years various studies showed:

The effectiveness of exercise in reducing back pain and preventing lumbopelvic pain in pregnancy [17-19, 101, 119]

Prenatal exercise decreased the severity of low back, pelvic girdle, and lumbopelvic pain during and following pregnancy [17]

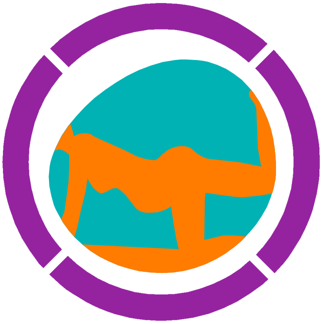

Exercise reduced the risk of low back pain in pregnancy by 9% and prevented sick leave due to lumbopelvic pain [120]

Exercise appears to reduce the risk of low back pain in pregnant women, but there is no clear evidence for an effect on pelvic girdle pain [18]

However, in the listed studies, exercise interventions varied in type, frequency, intensity and duration, hindering a possible association of specific types of exercise interventions with these outcomes [101].

11.5. Depression and mental disorders

The prevalence of depression during the first trimester of pregnancy was reported as 7.4-11%; in the second trimester 12.8%; and in the third trimester 8.5-12%. The prevalence at 40 weeks of pregnancy was 18.4% [121, 122]. Practice of physical activity and exercise is considered as a therapeutic complement to pharmacological treatment even in major depression cases [123]. In the last few years various studies showed:

Exercise in pregnancy may prevent perinatal depression and anxiety [101]

Light-to-moderate aerobic exercise improves mild-to-moderate depressive symptoms, and increases the likelihood that mild-to-moderate depression will resolve in the postpartum period [21]

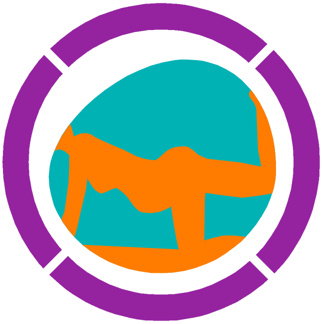

Prenatal exercise reduced the odds and severity of prenatal depression [22, 23]

Sufficient physical activity was associated with a reduced likelihood of probable antenatal depression and trait anxiety symptoms [124]

11.6. Urinary incontinence

Pelvic floor muscle dysfunctions can lead to urinary incontinence, a condition which often affects women during pregnancy and postpartum. Urinary incontinence (UI) is prevalent in antenatal and postnatal women, and pelvic floor muscle training (PFMT) is the first-line treatment for UI [15, 125, 126]. In case of symptoms, assessment and training by a gynecologist/midwife and women's health physiotherapist is indicated [56]. Current evidence supports that:

PFMT (e.g., Kegel exercises) may be performed on a daily basis to strengthen the pelvic floor muscles and to reduce the odds of urinary incontinence [1, 37, 38, 44, 45]

According to AGDH guidelines [58], pelvic floor exercises strengthen and tone the pelvic floor muscles and other tissues. Moreover, a strong pelvic floor can reduce the chance of complications (such as UI) after giving birth and later in life

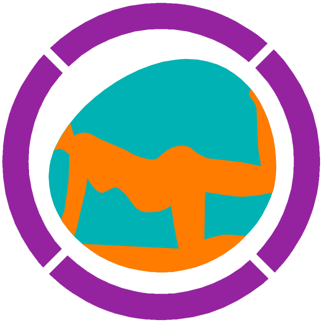

Structured pelvic floor muscle training in early pregnancy for continent women may prevent the onset of UI in late pregnancy and postpartum [15]

According to RANZCOG guidelines [45], activities that involve jumping or bouncing may add extra load to the pelvic floor muscles and should be avoided. However, there is some evidence that women participating in high-low impact exercise program (containing jumps and runs) combined with pelvic floor muscle training, maintained urinary continence, and improved neuromuscular activity of pelvic floor muscles [127].

"...pelvic floor muscle training (PFMT) is the first-line treatment for UI..."

12 SAFETY ISSUES REGARDING EXERCISE DURING PREGNANCY

The following considerations ensure the safety of an exercise program for pregnant women:

12.1. Hydration

Pregnant women feel secure if a bathroom is available in proximity of the training location

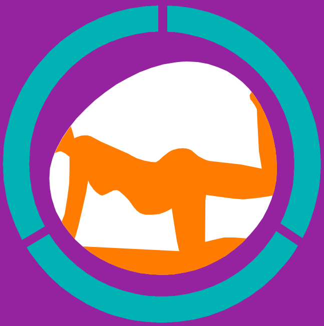

There are recommendations to increase hydration during pregnancy [1, 31, 42, 44, 58]. Pregnant women should drink water before, during and after exercise

UI during exercise is due to mechanical and anatomical changes. Considerations exist to minimize this complication, such as: voiding before activity; avoiding breath holding and use of Valsalva maneuver during exercise; practicing pelvic muscle-strengthening exercises; minimizing high-impact activities when incontinence symptoms appeared

12.2. Falls and injury

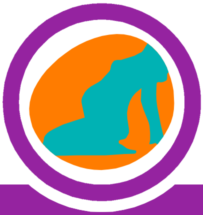

Pregnant women should avoid contact sports and sports or activities that may cause loss of balance or trauma to the mother and fetus (e.g., soccer, basketball, ice hockey, rollerblading, horseback riding, skiing, snowboarding, scuba diving, and vigorous intensity racquet sports) [1, 27, 28, 44, 56]. However, in the absence of medical contraindications, the decision to stop or continue particular disciplines should be based on the assessment of women's individual abilities, skills, previous experience and their sense of security and comfor

Increased body weight is associated with increased loading at the joints [45, 95]. Thus, weight-supported activities such as water-based exercise or stationary cycling may be more comfortable compared with weight-bearing exercises such as walking or jogging in the later stages of pregnancy [45]

During pregnancy an increase in the laxity of the musculoskeletal system is a natural adaptive process. There is a significant increase in joint laxity in five of seven peripheral joints over the course of the pregnancy and postpartum [128]

An exercise program employing minimal to moderate weight-bearing did not result in any measurable increases in knee laxity and, therefore, appears to be appropriate with regard to knee stability [129]

According to the RANZOG guidelines [45], the increase in ligament laxity associated with pregnancy may have implications for the injury risk. However, no scientific evidence proves a prevalence of joint injury related to physical activity in pregnant women. Activities that require jumping movements and quick changes in direction (e.g., court sports, aerobic dancing, etc.) should therefore be performed with caution [45]

According to the SMA guidelines [43], stretching exercises are useful but should be done gently due to the increased joint laxity during pregnancy. Because of increased relaxation of ligaments in pregnancy, joints are supported less effectively, especially in women with poor muscle mass. Activities that may result in excessive joint stress should be discontinued or adapted

The altered center of gravity resulting from the change in weight distribution as pregnancy progresses may influence balance [45, 95]. Thus, precaution should be taken to modify the exercise routine to minimize or avoid fast changes in direction, if necessary [45]

Balance exercises can improve the ability to resist forces within or outside of the body that cause falls while a person is stationary or moving. Strengthening muscles of the back, abdomen, and legs also improves balance [28]

When jogging, running or cycling, rocky terrains or unstable grounds should be avoided, since the joints are more lax in pregnancy, and ankle sprains and other injuries may occur.

12.3. Nausea and dizziness

For all activity types, the Valsalva maneuver, prolonged isometric contractions, and prolonged (motionless) standing should be avoided [27]

Exercise should always be completed with a cool-down and never stopped suddenly [45]

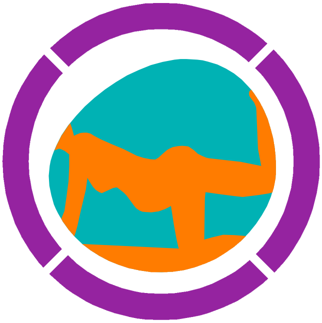

Some women will need to avoid physical activity in the supine position notably after week 16 of pregnancy [31, 44]**. Due to the weight of the growing fetus, exertion or prolonged periods in the supine position may reduce venous return and cardiac output** [27]

Fast movement changes in the vertical plane (e.g., from lying or sitting to standing; fast stand to sit and sit to stand) **are associated with a reduction in blood pressure and may cause dizziness and imbalance**

12.4. Heat, humidity and environment

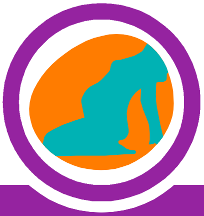

Hot ambient conditions and associated heat stress can increase adverse pregnancy outcomes and negatively affect mental health [130]

Evidence suggests that during exercise, evaporative (sweating) and dry (skin blood flow and temperature) heat loss responses increase from early to late pregnancy in addition to greater cardiac output, blood volume and reduced vascular resistance [131]

Pregnant women should avoid exercising in a hot humid environment, be well hydrated at all times, and dress appropriately to avoid heat stress [1, 27, 31, 37 38, 44, 58]

Prolonged exercise should be performed in a thermoneutral environment or in controlled environmental conditions (air conditioning) with close attention paid to proper hydration and caloric intake [31, 44]

Pregnant women are sensitive to smell. Exercise should be performed in a clean environment and avoid air pollution

Women should choose a place with adequate space and floor surface, ventilation, and temperature, as well as proper exercise equipment or common household materials. The key-point is safety, i.e., if there is no bench step, it is preferably to perform the step exercise on the floor (i.e., "invisible step") rather than use an inadequate bench. Another example is to use a stable and not slippy chair, if the exercise program is performed on a chair (e.g., [63])

In general, exercising outdoors – and in group – is preferable. Caution should be taken regarding the wind, the rain and slipping pavement while running or cycling. Proper sportswear and shoes, as well as adequate sun and head protection (e.g., hat or bike helmet) are other recommendations for exercising outdoor [41]

12.5. Sportswear and shoes

Endocrine changes occurring during pregnancy result in increased laxity of the ligaments of the foot, and several gait adaptations may occur [96, 132]

Sportswear should be comfortable, functional and (ideally) seamless due to the increased sensitivity of the skin.

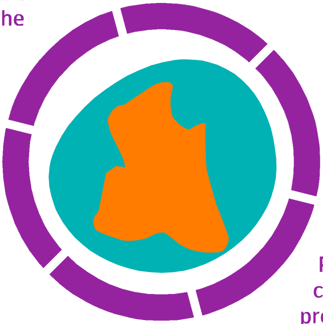

The AGDH guidelines [58] advise that pregnant women should wear appropriate shoes, non-restrictive clothing, and a supportive bra, and - in warm environments - loose clothing made from 'breathable' fabric.

Breasts become more sensitive during pregnancy. A supportive sports (or pregnancy specific) bra provides comfort during physical activities such as walking and running

Pregnant women should wear comfortable exercise footwear providing ankle and arch support, shock absorption and stability, especially during weight-bearing activities and outdoor activities [96]

Shoe specialists can provide recommendations to meet individual biomechanical profiles

12.6. Hight-intensity or prolonged exercise

Since competitive athletes have a strenuous training schedule throughout pregnancy and resume high-intensity postpartum training sooner as compared to other pregnant women, they require frequent and closer supervision [31]. Such athletes should pay particular attention to avoid hyperthermia, maintain proper hydration, and sustain adequate caloric intake to prevent weight loss, which may adversely affect fetal growth [31]

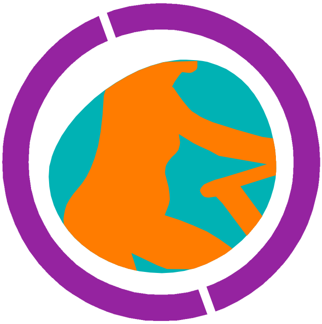

High-intensity or prolonged exercise over 45 minutes can lead to hypoglycemia; therefore, adequate caloric intake before exercise, or limiting the exercise session, is essential to minimize this risk [31]

13

RECOMMENDATIONS TO REDUCE TIME SPENT IN SEDENTARY BEHAVIOR

Sedentary behavior increases the risk of cardiovascular and cancer mortality, incident type 2 diabetes, and all-cause mortality [133]. During pregnancy, sedentary behaviors are associated with impaired glucose tolerance, high level of LDL[15] cholesterol and C reactive protein, mental distress, and larger newborn abdominal circumference [134]. Despite those negative consequences, pregnant women spent nearly two-thirds of their day in sedentary behaviors, independently of the trimester [135]. Furthermore, even those women that are compliant with physical activity guidelines at mid-pregnancy, did not reduce the time spent in sedentary behavior [136]. A recent cross-sectional study by Oviedo-Caro et al. [137] estimated a decrease of 4% in cardiorespiratory fitness and an increase of 6% in skinfold thickness adiposity when 30 minutes of moderate to vigorous physical activity was replaced by sedentary time.

The WHO's guidelines on physical activity and sedentary behavior extrapolated the recommendations for adult population for pregnant and postpartum women [1]. The WHO states that replacing sedentary time with physical activity of any intensity (including light intensity) provides health benefits. However, there is insufficient evidence to estimate health consequences for different domains of sedentary behavior, i.e., sitting or lying. In addition, more research is needed with regards to health benefits of breaking up prolonged periods of sedentary time [1]. The ACOG's guidelines do not include specific advice on sedentary behavior during pregnancy [31]. The Canadian Society for Exercise Physiology in their 24-hours Movement Guidelines for adults, encourage adults to limit sedentary time to 8 hours or less, by spending no more than 3 hours of recreational screen time and, by breaking up long periods of sitting as often as possible [138]. In addition, CSEP suggests that replacing sedentary behavior by light physical activity; and light physical activity by more moderate to vigorous physical activity - along with sufficient sleep - can provide greater health benefits [138].

In order to obtain an accurate and efficient measure of sedentary time, its definition and differentiation from physical inactivity needs to be understood, i.e., any waking activity involving energy expenditure less than 1.5 METs in a

[15] Low density lipoprotein cholesterol

inclinometer placed on the thigh allow to differentiate between sitting, lying, and standing postures, provide more sensitive measurement of sedentary time than accelerometers only [139].

In addition to objective measures of sedentary behavior, subjective measures are available for pregnant women. Several studies have used the subscale titled sedentary behavior from the Pregnancy Physical Activity Questionnaire (PPAQ) [77], although this subscale has shown poor agreement with devices combining accelerometer and inclinometer [140]. Another tool was designed to evaluate self-reported sedentary behavior in general population, the Sedentary Behavior Questionnaire (SBQ) [141]. This tool has been validated for pregnant women, however, the correlation with combined measures of accelerometer and physiological sensors is weak [142].

"...During pregnancy, sedentary behaviors are associated with impaired glucose tolerance, high level of LDL cholesterol and C reactive protein, mental distress, and larger newborn abdominal circumference..."

14

EXERCISE PRESCRIPTION FOR EARLY POSTPARTUM WOMAN

As pregnancy, the postpartum period provides an opportunity to encourage women to adopt a healthy and active lifestyle thereby improving body image, self-esteem, to lose excessive weight [143]. However, women may encounter personal and environmental factors limiting their physical activity after childbirth. Such factors include fatigue, lack of motivation and self-confidence, time constraints, lack of affordable and adequate activity, poor public transport connections, and childcare problems (leaving the baby to someone else) are the main causes of non-exercising [144]. Makama et al. [145] identified barriers and facilitators relating to capability (e.g., lack of knowledge regarding benefits of lifestyle behaviors; limitations in healthcare providers' skills in providing lifestyle support), opportunity (e.g., social support from partners, family, friends, and healthcare providers; childcare needs) and motivation (e.g., identifying benefits of exercise and perception of personal health; enjoyment of the activity). Thus, the authors suggest that lifestyle interventions for postpartum women should include the identified barriers and facilitators [145]. Moreover, there is a need to convey global guidelines in simple language (e.g., via educational lectures, visual information). Promising strategies include increasing knowledge, regular and individual counseling and support, self-monitoring with diaries and (if possible combined with pedometers), increasing self-efficacy, addressing barriers, referral to community resources for physical activity [146]. Moreover, initiation and adherence to physical activity can be promoted through (self-)organized mothers' groups, clubs or postnatal classes. Additionaly, the interaction between individuals, community, organizations, and policymakers is required [144].

There are several guidelines supporting the benefits of exercising during postpartum [53, 146]. Specific physiological, morphological, and musculoskeletal changes may persist for 4-6 weeks after birth, thus, the early postpartum period can be assumed as the “fourth trimester”. The immediate postpartum period focuses on recovery from delivery and caring for the infant [146], however, women's recovery from birth can be assisted through increased physical activity [144].

14.1. Exercise in the immediate postpartum period

Hospitalisation offers limited physical activity. However, simple exercises to stimulate the cardiorespiratory system and blood flow in particular muscles groups can be performed while walking through the hospital corridors or lying in bed. Some postural and light stretching exercises are also possible. Pelvic floor training (also known as Kegel exercises) can be implemented anytime and is important for a fast recovery [147, 148]. Moreover, those exercises are recommended to decrease the risk of urinary incontinence during and after pregnancy [41, 148-150].

14.2. Benefits of postpartum physical activity and exercise

The short-term benefits of postpartum physical activity and exercise include:

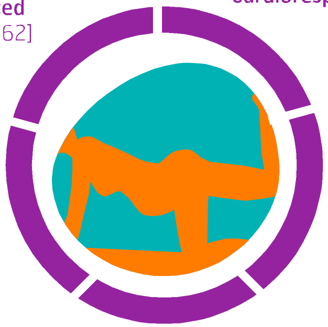

Factors for the association between physical activity and postnatal depression and/or weight loss changes were: supervision (one to one, and in group), structure (weekly frequency, scheduled durations and moderate intensity), guidelines-based over an extended postpartum period (e.g., more than 12 weeks) and supplemented by several psycho-social support strategies (e.g., educational information, exercise/ physical activity advice, and counselling) [157, 158]. Combined diet and physical exercise programs were more effective in reducing weight than physical exercise alone [163]. Moreover, the above listed studies indicate an urgent need to educate women in the topic of pelvic floor muscle training during the perinatal period.

14.3. General physical activity guidelines in postpartum

There is a lack of information on specific guidelines and concrete strategies of adapting several recreational and sports activities to healthy postpartum women. The main physical activity related recommendations are resumed in Box 7.

Box 7 MAIN PHYSICAL ACTIVITY RELATED RECOMMENDATIONS IN THE POSTPARTUM PERIOD, ADAPTED FROM [53]

General recommendations
Women should undertake regular physical activity throughout (pregnancy and) postpartum [1].
Frequency of recommended moderate-intensity physical activity for (pregnant and) postpartum women are comparable with the general adult population [1].
Women should do at least 150 minutes of moderate-intensity aerobic physical activity throughout the week for substantial health benefits, incorporating a variety of aerobic and muscle-strengthening activities. Adding gentle stretching may also be beneficial [1].
(Pregnant and) postpartum women should start by doing small amounts of physical activity, and gradually increase frequency, intensity, and duration over time [1].

If (pregnant and) postpartum women are not meeting the recommendations, doing some physical activity will benefit their health [1]. Even 10 minutes of exercise benefits the body [31]

Women should stop exercising if they feel pain [31]

Women should check with local fitness clubs or community centers for (group exercise) classes that interest them. Some gyms offer special postpartum exercise classes and classes where they can take with the baby. If women prefer to exercise on their own, they should check out fitness videos and online exercise programs designed for postpartum women [164]

Regarding breastfeeding

Regular aerobic exercise in lactating women has been shown to improve maternal cardiovascular fitness [31]

Mild- to moderate-intensity exercise during lactation does not affect the quantity or composition of breast milk or impact infant growth [31, 43, 57, 59], as long as there is appropriate food and fluid intake (the caloric cost of breast feeding is estimated to be about 600 kcal/day) [43]

Nursing women may find exercise more comfortable after breast feeding, to avoid the discomfort of engorged breasts during exercise [31, 43, 56, 57, 59, 164]

Mothers could feed their baby before exercise, postpone feeding to one hour after physical activity/exercise, or express milk before exercising, so that it may be used after the activity [43]

Breastfeeding women may find wearing a fitted bra with features of greater breast elevation more comfortable than a standard encapsulation sport bra (or compression) [56, 57]

Regarding low back pain and pelvic girdle pain

There is strong evidence that stabilization exercises generally are not more effective than any other form of active exercise in the long term, regarding low back pain and pelvic girdle pain [57]

Women presenting with low back and pelvic girdle pain at 6 weeks postpartum should be referred to a sport/women's health physiotherapist [56]

Regarding diastasis recti

To date, there is no evidence to guide women on which abdominal exercises are the most effective in reducing the distance between the two rectus muscles and strengthening the abdominals postpartum [56, 57].

In case of diastasis recti, women should be referred to a physiotherapist who will advise of some postnatal exercises and support garments that will help bring these muscles back together [45].

14.4. Pre-exercise assessment in the early postpartum

The postpartum exercise routine should gradually return to normal as soon as it is safe depending on the type of delivery and potential medical complications. Some women are capable of resuming physical activities within a few days after delivery. In the absence of medical or surgical complications, rapid resumption of exercise activities has not been found to result in adverse effects.

The following topics must be taken into consideration when planning an exercise program during the early postpartum period [41]:

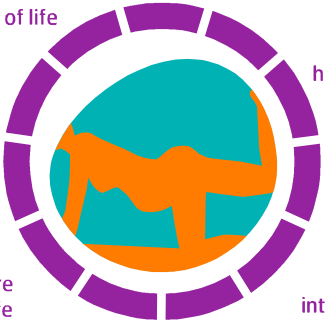

The following topics regarding pre-exercise assessment are addressed in the recommendations:

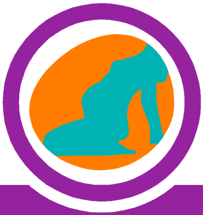

Postpartum resumption of physical activity is an individualized process [58] depending on health status [37]

If possible, women should try to get outside, take walks, or keep blood moving with a gentle workout [45]. Mild exercises should be able to be resumed immediately [164, 37].

Women without complications and discomfort may seek guidance from their health professionals before they start or re-start their physical activity/exercise regime [28, 37, 43, 58] and should seek guidance the case of delivery by caesarean section and surgical complications [1, 59, 164]

The decision when to restart exercise after cesarean section will depend on issues such as blood pressure, anemia, fatigue, pain management and wound healing [165]

Progression should be slower if there is discomfort or other relevant factors, such as anemia or wound infection [31, 45, 59, 164]

Independent of physical activity, the RANZCOG [45] general recommendations on when a postpartum woman should contact a doctor or midwife are the following:

Your doctor or midwife will want to see you for a postnatal checkup two to six weeks after the birth of your baby. Contact your doctor or midwife earlier, if you notice any of the following:

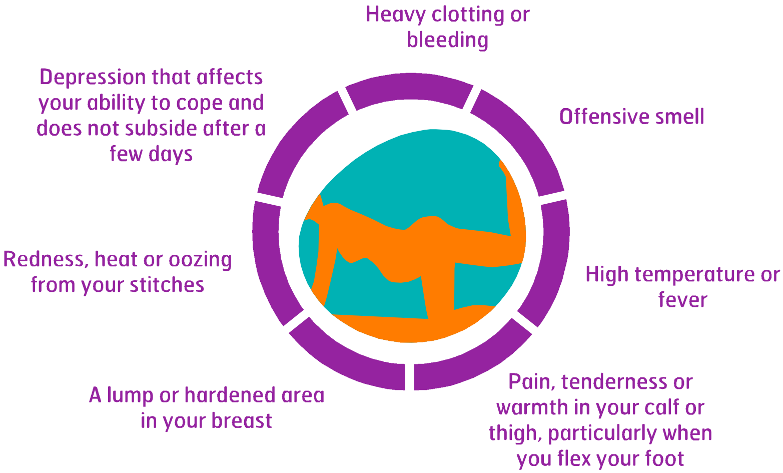

14.5. General exercise prescription components in postpartum

Box 8 resumes the exercise prescription components applied to the early postpartum period, adapted from Santos-Rocha et al. [41].

Box 8 MAIN PHYSICAL ACTIVITY RELATED RECOMMENDATIONS IN THE POSTPARTUM PERIOD, ADAPTED FROM [53]

Aerobic exercise

Exercise routines should incorporate a variety of aerobic activities [1]; exercises that activate large muscle groups in a rhythmic and continuous fashion

A variety of weight-bearing activities are well tolerated during the postpartum period

Women who habitually engaged in vigorous-intensity aerobic activity or who were physically active before pregnancy can continue these activities during pregnancy and the postpartum period [58]

Aerobic exercise should start gradually [56, 57, 164], and increasing exercise time, frequency and intensity as tolerated by their body [58]

In general, all healthy women should aim (through gradual progression) to accumulate 150-300 minutes of moderate-vigorous intensity aerobic exercise per week [1, 28, 43, 58, 164]. Moderate intensity exercise refers to 3-5.9 METs; RPE = 12-13; 40%-60% VO2reserve

Preferably, aerobic activity should be spread throughout the week [1, 28, 58]; 150 minutes can be divided into 30-minute workouts on 5 days of the week or into smaller 10-minute sessions throughout each day (e.g., three 10-minute walks each day) [164]

Women who habitually engaged in vigorous-intensity aerobic activity or who were physically active before pregnancy can continue these activities during pregnancy and the postpartum period [28]. If the woman exercised vigorously before pregnancy or she is a competitive athlete, she can work up to vigorous-intensity activity [164]. Low impact activities such as cross-country skiing, fast walking, low impact aerobics and step training put little pressure on the pelvic floor and can start soon after birth [56]. Walking and joining an exercise class (e.g., spinning and dance) are good ways to get daily exercise and get back in shape [164]

Resistance / strength exercise

Exercise routines should incorporate a variety of muscle-strengthening activities [1]

A variety of machines, free weights, and body weight exercises are well tolerated during the postpartum period

Strength exercise should start gradually [56, 57]**, and increasing exercise time, frequency and intensity as tolerated by their body** [57]

Intensity that permits multiple submaximal repetitions (i.e., 8-10 or 12-15 repetitions) to be performed to the point of moderate fatigue (40%-60% of estimated one repetition maximum)

First focus should be on abdominal and back muscles [56, 164]

It may be prudent for women whose delivery was complicated by a risk factor for levator ani muscle injury (anal sphincter tear, forceps delivery, long second stage, large baby) to minimize activities that generate large increases in intra-abdominal pressure for several months postpartum [57]

Pelvic floor muscles training

Complex training for pelvic-floor muscles should be focused both on their contraction and relaxation. Different exercises should be performed to improve pelvic floor muscle speed, strength, endurance and muscular coordination, and engaging both fast and slow twitch muscle fibers.

Proper technique should be ensured.

There is strong evidence for pelvic floor muscle training as prevention and treatment of urinary incontinence in the general postpartum population [45, 57]**.**

Pelvic floor muscles training should be performed during pregnancy, and can start directly after birth [31, 56, 58, 59, 164]**, at least 25 repetitions at various times of the day** [45]**.**

An effective intensity (and volume) of pelvic floor muscles exercise has not been determined, but it can be performed 10-20 min/day, 1-7 days/week.

Pelvic floor muscles training can be performed anywhere, anytime, every day.

Jumping exercises should be avoided in the early postpartum period due to the fragility of the pelvic floor [59].

Balance and coordinaion exercise

Exercises involving motor skill, e.g., balance, agility, coordination, gait), proprioceptive training, and multifaceted activities (e.g., Pilates, Yoga, tai chi) are well tolerated during the postpartum period.

Joining an exercise class (e.g., yoga, and Pilates) is a good way to get daily exercise and get back in shape [164].

Balance and/or coordination exercises can be included in daily activities (e.g., functional training).

Intensity in balance or coordination training refers to the degree of difficulty of the postures, movements, or routines practiced. An effective intensity (and volume) of neuromotor exercise has not been determined, but it can be performed 20-30 to 60 min/day, at least 2-3 up to 7 days/week.

Positions and movements that are uncomfortable or likely to result in loss of balance and falling should be avoided.

RPE = rating of perceived exertion (6-20 scale); METs = metabolic equivalents; $VO_{2reserve}$= oxygen uptake reserve (VO_{2max} – VO_{2rest})

14.6. Exercise selection and adaptation in the early postpartum

All the following types of physical activity are recommended to be implemented progressively in the postpartum period. The main question is when to start or to continue. Most women will have sufficiently recovered 4-6 weeks after a vaginal birth or surgery (i.e., cesarean section). Those women with normal delivery and puerperium can start earlier. Some activities will be limited until pelvic organs and musculoskeletal health structures are recovered.

Pregnancy and childbirth impact the maternal musculoskeletal system [56]. The amount of physical activity should increase until the baseline recommendations for adults are reached. Women who were already active before giving birth can gradually return to their usual level of physical activity as soon as they feel well, taking into account the mode of birth, any injuries related to the delivery and the stability of the perineum. To get back to the same level as before and to start a high level of solicitation, it is recommended to wait until the postnatal consultation 6 to 8 weeks after delivery. This recovery period may be longer for some women, especially for high-impact forms of physical activity such as running or activities involving jumping. These activities should be implemented after full recovery of pelvic organs and musculoskeletal structures.

According to Swiss Health Promotion [166] "Getting enough physical activity after childbirth is also important for the health and well-being. Even for women who were inactive before giving birth, starting to exercise physical activity has many benefits. In the medium term, the amount of physical activity should increase until the baseline recommendations for adults are reached. Women who were already active before giving birth can gradually return to their usual level of physical activity as soon as they feel well, taking into account the mode of birth, any injuries related to the delivery and the stability of the perineum. To get back to the same level as before and to start a high level of solicitation it is recommended to wait until the postnatal consultation 6 to 8 weeks after delivery. Depending on the woman, this waiting time may be longer, especially for high-impact forms of physical activity such as running or activities involving jumping. Exercises to strengthen the perineum are particularly recommended for all women after giving birth".

Box 9 lists the recommended exercises to be implemented in the early postpartum, and to be implemented after full recovery of pelvic organs and musculoskeletal structures.

Box 9 RECOMMENDED EXERCISES TO BE IMPLEMENTED IN THE EARLY POSTPARTUM, ADAPTED FROM [41]

Exercises to be implemented in the early postpartum (i.e., up to 6-8 weeks)
Pelvic floor muscle training
Walking indoor and walking outdoor
Low-impact Aerobics, Dancing, Step, “Invisible Step”
Pilates and Core training
Stretching exercises
Light resistance training
Posture and functional training
Balance training

Exercises to be implemented in the postpartum (e.g., after 12 weeks)
Indoor and outdoor cycling (using different types of bikes) are among the recommended types of exercise during the postpartum period, after full recovery of pelvic organs and musculoskeletal structures
Jogging or running
Water exercise and swimming
More intense functional and strength training are among the recommended types of exercise during the postpartum period, after full recovery of pelvic organs and musculoskeletal structures

Further explanation of exercise selection and adaptation during the postpartum period can be found elsewhere [39-41]. More examples of exercises and workouts of different activities can be found in our YouTube Channels: Active Pregnancy [63] and Active at home & outdoors [64].

REFERENCES

1. Bull FC, Al-Ansari SS, Biddle S, et al. World Health Organization 2020 guidelines on physical activity and sedentary behavior. British Journal of Sports Medicine. 2020;54:1451-62. Available at: https://bjsm.bmj.com/content/54/24/1451.long

2. Santos-Rocha R (Editor). Exercise and Physical Activity during Pregnancy and Postpartum. Evidence-Based Guidelines. 2 ed. Switzerland: Springer International Publishing, 2022.

3. Barakat R, Perales M, Bacchi M, Coteron J, Refoyo I. A program of exercise throughout pregnancy. Is it safe to mother and newborn? Am J Health Promot 2013;29(1): 2-8.

4. Perales M, Santos-Lozano A, Ruiz JR, Lucia A, Barakat R. Benefits of aerobic or resistance training during pregnancy on maternal health and perinatal outcomes: A systematic review. Early Hum Dev. 2016 Mar;94:43-8.

5. Dipietro L, Evenson KR, Bloodgood B, Sprow K, Troiano RP, Piercy KL, Vaux-Bjerke A, Powell KE; 2018 PHYSICAL ACTIVITY GUIDELINES ADVISORY COMMITTEE*. Benefits of Physical Activity during Pregnancy and Postpartum: An Umbrella Review. Med Sci Sports Exerc. 2019 Jun;51(6):1292-302.

6. Díaz-Burrueco JR, Cano-Ibáñez N, Martín-Peláez S, Khan KS, Amezcua-Prieto C. Effects on the maternal-fetal health outcomes of various physical activity types in healthy pregnant women. A systematic review and meta-analysis. Eur J Obstet Gynecol Reprod Biol 2021;262:203-215.

7. Davenport MH, Skow RJ, Steinback CD. Maternal Responses to Aerobic Exercise in Pregnancy. Clin Obstet Gynecol. 2016 Sep;59(3):541-51.

8. Morales-Suárez-Varela M, Clemente-Bosch E, Peraita-Costa I, Llopis-Morales A, Martínez I, Llopis-González A. Maternal Physical Activity During Pregnancy and the Effect on the Mother and Newborn: A Systematic Review. J Phys Act Health. 2020 Dec 22;18(1):130-47.

9. Magro-Malosso ER, Saccone G, Di Tommaso M, Roman A, Berghella V. Exercise during pregnancy and risk of gestational hypertensive disorders: a systematic review and meta-analysis. Acta Obstet Gynecol Scand. 2017 Aug;96(8):921-31.

10. Davenport MH, Ruchat SM, Poitras VJ, Jaramillo Garcia A, Gray CE, Barrowman N, Skow RJ, Meah VL, Riske L, Sobierajski F, James M, Kathol AJ, Nuspl M, Marchand AA, Nagpal TS,

Slater LG, Weeks A, Adamo KB, Davies GA, Barakat R, Mottola MF. Prenatal exercise for the prevention of gestational diabetes mellitus and hypertensive disorders of pregnancy: a systematic review and meta-analysis. Br J Sports Med. 2018 Nov;52(21):1367-75.

11. Harrison AL, Shields N, Taylor NF, Frawley HC. Exercise improves glycaemic control in women diagnosed with gestational diabetes mellitus: a systematic review. J Physiother. 2016 Oct;62(4):188-96.

12. Bgeginski, R., Ribeiro, P. A.B., Mottola, M. F. and Ramos, J. G. L. (2017), Effects of weekly supervised exercise or physical activity counseling on fasting blood glucose in women diagnosed with gestational diabetes mellitus: A systematic review and meta-analysis of randomized trials. Journal of Diabetes, 9: 1023–32.

13. Brown J, Ceysens G, Boulvain M. Exercise for pregnant women with gestational diabetes for improving maternal and fetal outcomes. Cochrane Database Syst Rev. 2017 Jun 22;6(6):CD012202.

14. Shepherd E, Gomersall JC, Tieu J, Han S, Crowther CA, Middleton P. Combined diet and exercise interventions for preventing gestational diabetes mellitus. Cochrane Database Syst Rev. 2017 Nov 13;11(11):CD010443.

15. Mørkved S, Bø K. Effect of pelvic floor muscle training during pregnancy and after childbirth on prevention and treatment of urinary incontinence: a systematic review. Br J Sports Med. 2014 Feb;48(4):299-310.

16. Domenjoz I, Kayser B, Boulvain M. Effect of physical activity during pregnancy on mode of delivery. Am J Obstet Gynecol. 2014 Oct;211(4):401.e1-11.

17. Liddle SD, Pennick V. Interventions for preventing and treating low-back and pelvic pain during pregnancy. Cochrane Database of Systematic Reviews 2015, Issue 9. Art. No.: CD001139.

18. Davenport MH, Marchand AA, Mottola MF, Poitras VJ, Gray CE, Jaramillo Garcia A, Barrowman N, Sobierajski F, James M, Meah VL, Skow RJ, Riske L, Nuspl M, Nagpal TS, Courbalay A, Slater LG, Adamo KB, Davies GA, Barakat R, Ruchat SM. Exercise for the prevention and treatment of low back, pelvic girdle and lumbopelvic pain during pregnancy: a systematic review and meta-analysis. Br J Sports Med. 2019 Jan;53(2):90-98.

19. Shiri R, Coggon D, Falah-Hassani K. Exercise for the prevention of low back and pelvic girdle pain in pregnancy: A meta-analysis of randomized controlled trials. Eur J Pain. 2018 Jan;22(1):19-27.

20. Daley AJ, Foster L, Long G, Palmer C, Robinson O, Walmsley H, Ward R. The effectiveness of exercise for the prevention and treatment of antenatal depression: systematic review with meta-analysis. BJOG 2015 Jan; 122(1):57-62.

21. McCurdy AP, Boulé NG, Sivak A, Davenport MH. Effects of Exercise on Mild-to-Moderate Depressive Symptoms in the Postpartum Period: A Meta-analysis. Obstet Gynecol. 2017 May 5.

22. Davenport MH, McCurdy AP, Mottola MF, Skow RJ, Meah VL, Poitras VJ, Jaramillo Garcia A, Gray CE, Barrowman N, Riske L, Sobierajski F, James M, Nagpal T, Marchand AA, Nuspl M, Slater LG, Barakat R, Adamo KB, Davies GA, Ruchat SM. Impact of prenatal exercise on both prenatal and postnatal anxiety and depressive symptoms: a systematic review and meta-analysis. Br J Sports Med. 2018 Nov;52(21):1376-1385.

23. Nakamura A, van der Waerden J, Melchior M, Bolze C, El-Khoury F, Pryor L. Physical activity during pregnancy and postpartum depression: Systematic review and meta-analysis. J Affect Disord. 2019 Mar 1;246:29-41.

24. Bø K, Artal R, Barakat R, Brown W, Dooley M, Evenson KR, Haakstad LA, Larsen K, Kayser B, Kinnunen TI, Mottola MF, Nygaard I, van Poppel M, Stuge B, Davies GA; IOC Medical Commission. Exercise and pregnancy in recreational and elite athletes: 2016 evidence summary from the IOC expert group meeting, Lausanne. Part 2-the effect of exercise on the fetus, labour and birth. Br J Sports Med. 2016 Oct 12. pii: bjsports-2016-096810.

25. Caspersen CJ, Powell KE, Christenson GM. Physical activity, exercise, and physical fitness: definitions and distinctions for health-related research. Public Health Rep. 1985;100(2):126–31.

26. ACSM - American College of Sports Medicine. ACSM's Resources for the Exercise Physiologist (2nd ed). Wolters Kluwer, 2018.

27. ACSM - American College of Sports Medicine. ACSM's Guidelines for Exercise Testing and Prescription. 11 ed. Wolters Kluwer Health, 2021.

28. USDHHS - U.S. Department of Health and Human Services. Physical Activity Guidelines for Americans. 2 ed. Washington, DC: U.S. Department of Health and Human Services; 2018. Available at: https://health.gov/sites/default/files/2019-09/Physical_Activity_Guidelines_2nd_edition.pdf

29. Kohl HW 3rd, Craig CL, Lambert EV, Inoue S, Alkandari JR, Leetongin G, Kahlmeier S; Lancet Physical Activity Series Working Group. The pandemic of physical inactivity: global action for public health. Lancet 2012;380(9838):294-305.

30. Blair SN. Physical inactivity: the biggest public health problem of the 21st century. Br J Sports Med. 2009;43:1-2.

31. ACOG – American College of Obstetricians and Gynecologists. ACOG Committee Opinion No. 804: Physical Activity and Exercise During Pregnancy and the Postpartum Period. Obstetrics and Gynecology [Internet]. 2020; 135(4):[e178-e88 pp.]. Available at: https://www.acog.org/clinical/clinical-guidance/committee-opinion/articles/2020/04/physical-activity-and-exercise-during-pregnancy-and-the-postpartum-period

32. Okafor UB, Goon DT. Providing physical activity education and counselling during pregnancy: A qualitative study of midwives' perspectives. Nig. J. Clin. Pract. 2021;24, 718-28.

33. Findley A, Smith DM, Hesketh K, Keyworth C. Exploring womens' experiences and decision making about physical activity during pregnancy and following birth: A qualitative study. BMC pregnancy and childbirth. 2020;20(1), 1-10.

34. OMS bureau ré'8Egional de l'Europe. Orientations straté'8Egiques europé'8Eennes relatives au renforcement des soins infirmiers et obstétricaux dans le cadre des objectifs de Santé 2020. Available at: https://www.euro.who.int/fr/health-topics/Health-systems/nursing-and-midwifery/publications/2015/european-strategic-directions-for-strengthening-nursing-and-midwifery-towards-health-2020-goal

35. Petrov Fieril K, Fagevik Olsén M, Glantz A, Larsson M. Experiences of exercise during pregnancy among women who perform regular resistance training: a qualitative study. Physical Therapy. 2014;94(8):1135-43.

36. Kilpatrick SJ, Papile LA, Macones GA. Guidelines for Perinatal Care. 8 ed. AAP Committee on Fetus and Newborn and ACOG Committee on Obstetric Practice, 2017. Available at: https://ebooks.aappublications.org/content/guidelines-for-perinatal-care-8th-edition.tab-info

37. ACSM - American College of Sport Medicine. ACSM information on Pregnancy Physical Activity. American College of Sports Medicine; 2020. Available at: https://www.acsm.org/docs/default-source/files-for-resource-library/pregnancy-physical-activity.pdf?sfvrsn=12a73853_4

38. EIM/ACSM. Being Active during Pregnancy. Exercise is Medicine/American College of Sports Medicine 2019. Available at: https://www.exerciseismedicine.org/assets/page_documents/EIM_Rx%20for%20Health_Pregnancy.pdf

39. Santos-Rocha R, Corrales-Gutierrez I, Szumilewicz A, Pajaujiene S. Exercise testing and prescription during pregnancy. In R. Santos-Rocha, Editor, Exercise and Physical Activity during Pregnancy and Postpartum. Evidence-Based Guidelines. 2 ed. Switzerland: Springer International Publishing; 2022, Ch.8.

40. Szumilewicz A and Santos-Rocha R. Exercise selection during pregnancy. In Santos-Rocha, Editor, Exercise and Physical Activity during Pregnancy and Postpartum. Evidence-Based Guidelines. 2 ed. Switzerland: Springer International Publishing; 2022, Ch.9.

41. Santos-Rocha R, Szumilewicz A and Pajaujiene S. Exercise prescription and adaptations in early postpartum. In Santos-Rocha, Editor, Exercise and Physical Activity during Pregnancy and Postpartum. Evidence-Based Guidelines. 2 ed. Switzerland: Springer International Publishing; 2022, Ch.10.

42. NHS - National Health Service. Exercise in Pregnancy; 2020. Available at: https://www.nhs.uk/pregnancy/keeping-well/exercise/

43. SMA – Sports Medicine Australia. Pregnancy and Exercise. Women in Sport. 2017. Available at: https://sma.org.au/sma-site-content/uploads/2017/08/SMA-Position-Statement-Exercise-Pregnancy.pdf

44. Mottola MF, Davenport MH, Ruchat S-M, Davies GA, Poitras VJ, Gray CE, et al. 2019 Canadian guideline for physical activity throughout pregnancy. British Journal of Sports Medicine. 2018;52(21):1339-46. Available at: https://bjsm.bmj.com/content/52/21/1339

45. RANZCOG - The Royal Australian and New Zealand College of Obstetricians and Gynaecologists. Exercise in Pregnancy. RANZCOG, 2020. Available at: https://ranzcog.edu.au/womens-health/patient-information-resources/exercise-during-pregnancy

46. EuropeActive. EuropeActive Standards - European Qualification Framework level 5 - Pregnancy and Postnatal Exercise Specialist, 2016. Available at: http://www.ehfa-standards.eu/es-standards

47. Atkinson L and Teychenne M. Psychological, social and behavioural changes during pregnancy: implications for physical activity and exercise. In Santos-Rocha, Editor, Exercise and Physical Activity during Pregnancy and Postpartum. Evidence-Based Guidelines. 2 ed. Switzerland: Springer International Publishing; 2022, Ch.2.

48. Zinsser LA, Stoll K, Wieber F, Pehlke-Milde J, Gross MM. Changing behaviour in pregnant women: A scoping review. Midwifery. 2020 Jun;85:102680.

49. Michie S, van Stralen MM, West R. The behaviour change wheel: A new method for characterising and designing behaviour change interventions. Implement Sci. 2011;6(1):42.

50. Wegrzyk J, Hyvärinen M, De Labrusse C, Schläppy F. Strengthening competences of future healthcare professionals to promote physical activity during pregnancy and post-partum. In R. Santos-Rocha, Editor, Exercise and Physical Activity during Pregnancy and Postpartum. Evidence-Based Guidelines. 2 ed. Switzerland: Springer International Publishing; 2022, Ch.11.

51. Tanha FD, Ghajarzadeh M, Mohseni M, Shariat M, Ranjbar M. Is ACOG Guideline Helpful for Encouraging Pregnant Women to Do Exercise During Pregnancy? Acta Medica Iranica. 2014;52(6):458-61.

52. Savvaki D, Taousani E, Goulis DG, Tsirou E, Voziki E, Douda H, Nikolettos N, Tokmakidis SP. Guidelines for exercise during normal pregnancy and gestational diabetes: a review of international recommendations. Hormones (Athens). 2018 Dec;17(4):521-529.

53. Szumilewicz A, Worska A, Santos-Rocha R, Oviedo-Caro MA. Evidence-based and practice-oriented guidelines for exercising during pregnancy. In R. Santos-Rocha, Editor, Exercise and Physical Activity during Pregnancy and Postpartum. Evidence-Based Guidelines. 2 ed. Switzerland: Springer International Publishing; 2022, Ch.7.

54. Evenson KR, Mottola MF, Artal R. Review of Recent Physical Activity Guidelines During Pregnancy to Facilitate Advice by Health Care Providers. Obstet Gynecol Surv. 2019 Aug;74(8):481-9.

55. Tsakiridis I, Bakaloudi DR, Oikonomidou AC, Dagklis T, Chourdakis M. Exercise during pregnancy: a comparative review of guidelines. J Perinat Med. 2020 Jul 28;48(6):519-525.

56. Bø K, Artal R, Barakat R, Brown WJ, Davies GAL, Dooley M, et al. Exercise and pregnancy in recreational and elite athletes: 2016/2017 evidence summary from the IOC expert group meeting, Lausanne. Part 5 - Recommendations for health professionals and active women. British journal of sports medicine. 2018;52(17):1080-5. Available at: https://bjsm.bmj.com/content/52/17/1080.long

57. Bø K, Artal R, Barakat R, Brown WJ, Davies GAL, Dooley M, Evenson KR, Haakstad LAH, Kayser B, Kinnunen TI, Larsén K, Mottola MF, Nygaard I, van Poppel M, Stuge B, Khan KM; IOC Medical Commission. Exercise and pregnancy in recreational and elite athletes: 2016/17 evidence summary from the IOC Expert Group Meeting, Lausanne. Part 3 - Exercise in the postpartum period. Br J Sports Med. 2017 Nov;51(21):1516-25. Available at: https://bjsm.bmj.com/content/51/21/1516.long

58. AGDH - Australian Government. Department of Health. Guidelines for physical activity during pregnancy, 2021. Available at: https://www.health.gov.au/resources/publications/physical-activity-and-exercise-during-pregnancy-guidelines-brochure

59. Campos MDSB, Buglia S, Colombo CSSS, Buchler RDD, Brito ASX, Mizzaci CC, Feitosa RHF, Leite DB, Hossri CAC, Albuquerque LCA, Freitas OGA, Grossman GB, Mastrocola LE. Position Statement on Exercise During Pregnancy and the Post-Partum Period - 2021. Arq Bras Cardiol. 2021 Jul;117(1):160-80. English, Portuguese. Available at: https://www.ncbi.nlm.nih.gov/pmc/articles/PMC8294738/#S01

60. Prochaska JO, DiClemente CC, Norcross JC. In search of how people change: Applications to addictive behaviors. American psychologist, 1992, 47(9), 1102.

61. Broberg L, Ersbøll AS, Backhausen MG, Damm P, Tabor A, Hegaard HK. Compliance with national recommendations for exercise during early pregnancy in a Danish cohort. BMC Pregnancy Childbirth. 2015;15(1):317.

62. Huberty JL, Buman MP, Leiferman JA, Bushar J, Adams MA. Trajectories of objectively-measured physical activity and sedentary time over the course of pregnancy in women self-identified as inactive. Preventive Medicine Reports. 2016;3:353-360.

63. Active at home / Active outdoors YouTube Chanel: https://www.youtube.com/channel/UCEUWdoBeh5rgfM0kZOn9Xtg/videos

64. Active Pregnancy YouTube Chanel: https://www.youtube.com/channel/UC0VyookwcOmcQ5T70imtoNA/playlists

65. ASC - Australian Sports Commission. Pregnancy and Sport: Guidelines for the Australian sporting industry, 2002.

66. Szymanski LM, Satin AJ. Strenuous exercise during pregnancy: is there a limit? Am J Obstet Gynecol. 2012 Sep;207(3):179.e1-6.

67. Pivarnik JM, Szymanski LM, Conway MR. The Elite Athlete and Strenuous Exercise in Pregnancy. Clin Obstet Gynecol. 2016 Sep;59(3):613-9.

68. CSEP - Canadian Society for Exercise Physiology. GET ACTIVE QUESTIONNAIRE FOR PREGNANCY. 2021 Available at: https://csep.ca/wp-content/uploads/2021/05/GAQ_P_English.pdf

69. CSEP - Canadian Society for Exercise Physiology. HEALTH CARE PROVIDER CONSULTATION FORM FOR PRENATAL PHYSICAL ACTIVITY. 2021 Available at: https://csep.ca/wp-content/uploads/2021/05/GAQ_P_HCP_English.pdf

70. Foxcroft KF, Callaway LK, Byrne NM, Webster J. Development and validation of a pregnancy symptoms inventory. BMC Pregnancy Childbirth. 2013 Jan 16;13:3.

71. PAR-Q+ Collaboration. Physical Activity Readiness Questionnaire for Everyone (PAR-Q+), 2017. Available at: http://eparmedx.com/

72. Tang MSS, Moore K, McGavigan A, Clark RA, Ganesan AN. Effectiveness of Wearable Trackers on Physical Activity in Healthy Adults: Systematic Review and Meta-Analysis of Randomized Controlled Trials. JMIR Mhealth Uhealth. 2020 Jul 22;8(7):e15576.

73. Conway MR, Marshall MR, Schlaff RA, Pfeiffer KA, Pivarnik JM. Physical Activity Device Reliability and Validity during Pregnancy and Postpartum. Med Sci Sports Exerc. 2018 Mar;50(3):617-623.

74. Hesketh KR, Evenson KR, Stroo M, Clancy SM, Østbye T, Benjamin-Neelon SE. Physical activity and sedentary behavior during pregnancy and postpartum, measured using hip and wrist-worn accelerometers. Prev Med Rep. 2018 Apr 19;10:337-345.

75. Kinnunen TI, Tennant PW, McParlin C, Poston L, Robson SC, Bell R. Agreement between pedometer and accelerometer in measuring physical activity in overweight and obese pregnant women. BMC Public Health. 2011 Jun 27;11:501.

76. Craig CL, Marshall AL, Sjostrom M, Bauman AE, Booth ML, Ainsworth BE. International physical activity questionnaire: 12-country reliability and validity. Med Sci Sports Exerc., 2003, 35: 1381-95.

77. Chasan-Taber L, Schmidt MD, Roberts DE, Hosmer D, Markenson G, Freedson PS. Development and validation of a Pregnancy Physical Activity Questionnaire. Med Sci Sports Exerc., 2004; 36(10):1750-60.

78. Wowdzia JB, Davenport MH. Cardiopulmonary exercise testing during pregnancy. Birth Defects Res. 2021 Feb 1;113(3):248-64.

79. Mottola MF, Davenport MH, Brun CR, Inglis SD, Charlesworth S, Stopper MM. VO2peak prediction and exercise prescription for pregnant women. Med Sci Sports Exerc. 2006; 38(8):1389-95.

80. Gellish RL, Goslin BR, Olson RE, McDonald A, Russi GD, Moudgil VK. Longitudinal modeling of the relationship between age and maximal heart rate. Med Sci Sports Exerc. 2007;39(5):822–9.

81. O´Toole ML and Artal R. Clinical Exercise Testing during Pregnancy and the Postpartum Period. In Weisman IM, Zeballos RJ (eds): Clinical Exercise Testing. Prog Respir Res. Basel, Karger, 2002, vol 32, pp 273-81.

82. Wolfe LA. Pregnancy. In Skinner JS (ed): Exercise Testing and Exercise Prescription for Special Cases: Theoretical Basis and Clinical Application (3rd ed). Baltimore: Lippincott Williams & Wilkins; 2005. pp 377–91.

83. Dennis AT, Salman M, Paxton E, Flint M, Leeton L, Roodt F, Yentis S, Dyer RA. Resting Hemodynamics and Response to Exercise Using the 6-Minute Walk Test in Late Pregnancy: An International Prospective Multicentre Study. Anesth Analg. 2019 Aug;129(2):450-7.

84. O'Connor PJ, Poudevigne MS, Johnson KE, Brito de Araujo J, Ward-Ritacco CL. Effects of Resistance Training on Fatigue-Related Domains of Quality of Life and Mood During Pregnancy: A Randomized Trial in Pregnant Women With Increased Risk of Back Pain. Psychosom Med. 2018 Apr;80(3):327-32.

85. Fitzgerald CM and Segal NA (eds.), Musculoskeletal Health in Pregnancy and Postpartum, 2015. Springer International Publishing Switzerland.

86. Ortega FB, Ruiz JR, España-Romero V, Vicente-Rodriguez G, Martínez-Gómez D, Manios Y, Béghin L, Molnar D, Widhalm K, Moreno LA, Sjöström M, Castillo MJ; HELENA study group. The International Fitness Scale (IFIS): usefulness of self-reported fitness in youth. Int J Epidemiol. 2011 Jun;40(3):701-11.

87. Romero-Gallardo L, Soriano-Maldonado A, Ocón-Hernández O, Acosta-Manzano P, Coll-Risco I, Borges-Cosic M, Ortega FB, Aparicio VA. International Fitness Scale-IFIS: Validity and association with health-related quality of life in pregnant women. Scand J Med Sci Sports. 2020 Mar;30(3):505-14.

88. Henström M, Leppänen MH, Henriksson P, Söderström E, Sandborg J, Ortega FB, Löf M. Self-reported (IFIS) versus measured physical fitness, and their associations to cardiometabolic risk factors in early pregnancy. Sci Rep. 2021 Nov 22;11(1):22719.

89. Pimenta N and van Poppel M. Body composition changes during pregnancy and effects of physical exercise. In Santos-Rocha, Editor, Exercise and Physical Activity during Pregnancy and Postpartum. Evidence-Based Guidelines. 2 ed. Switzerland: Springer International Publishing; 2022, Ch.4

90. Silva MRG, Rodriguez Doñate B, Che Carballo KN. Nutritional requirements for the pregnant exerciser and athlete. In R. Santos-Rocha, Editor, Exercise and Physical Activity during Pregnancy and Postpartum. Evidence-Based Guidelines. 2 ed. Switzerland: Springer International Publishing; 2022, Ch.13.

91. Jorge R, Teixeira D, Ferreira I, Alvarez-Fálcon AL. Diet Recommendations for the Pregnant Exerciser and Athlete. In R. Santos-Rocha, Editor, Exercise and Physical Activity during Pregnancy and Postpartum. Evidence-Based Guidelines. 2 ed. Switzerland: Springer International Publishing; 2022, Ch.14.

92. Garber CE, Blissmer B, Deschenes MR, Franklin BA, Lamonte MJ, Lee IM, Nieman DC, Swain DP; American College of Sports Medicine. American College of Sports Medicine

position stand. Quantity and quality of exercise for developing and maintaining cardiorespiratory, musculoskeletal, and neuromotor fitness in apparently healthy adults: guidance for prescribing exercise. Med Sci Sports Exerc. 2011 Jul;43(7):1334-59.

93. Melzer K, Schutz Y, Boulvain M, Kayser B. Physical activity and pregnancy: cardiovascular adaptations, recommendations and pregnancy outcomes. Sports Med. 2010 Jun 1;40(6):493-507.

94. Perales M, Nagpal TS, Barakat R. Physiological changes during pregnancy. Main adaptations and discomforts and implications for physical activity and exercise. In Santos-Rocha, Editor, Exercise and Physical Activity during Pregnancy and Postpartum. Evidence-Based Guidelines. 2 ed. Switzerland: Springer International Publishing; 2022, Ch.3.

95. Bø K, Stuge B, Hilde G. Specific musculoskeletal adaptations in pregnancy: pelvic floor, pelvic girdle and low back pain. Implications for physical activity and exercise. In Santos-Rocha, Editor, Exercise and Physical Activity during Pregnancy and Postpartum. Evidence-Based Guidelines. 2 ed. Switzerland: Springer International Publishing; 2022, Ch.6.

96. Branco M, Santos-Rocha R, Aguiar L, Vieira F, Veloso AP. Biomechanical adaptations of gait in pregnancy. Implications for physical activity and exercise. In Santos-Rocha, Editor, Exercise and Physical Activity during Pregnancy and Postpartum. Evidence-Based Guidelines. 2 ed. Switzerland: Springer International Publishing; 2022, Ch.5.

97. ACOG. Your Pregnancy and Childbirth. 7 ed. American College of Obstetricians and Gynecologists, 2021.

98. Miquelutti MA, Cecatti JG, Makuch MY. Evaluation of a birth preparation program on lumbopelvic pain, urinary incontinence, anxiety and exercise: a randomized controlled trial. BMC Pregnancy Childbirth. 2013 Jul 29;13:154.

99. Miquelutti MA, Cecatti JG, Makuch MY. Developing strategies to be added to the protocol for antenatal care: an exercise and birth preparation program. Clinics (Sao Paulo). 2015 Apr;70(4):231-6.

100. Akca A, Corbacioglu Esmer A, Ozyurek ES, Aydin A, Korkmaz N, Gorgen H, Akbayir O. The influence of the systematic birth preparation program on childbirth satisfaction. Arch Gynecol Obstet. 2017 May;295(5):1127-33.

101. Ribeiro MM, Andrade A, Nunes I. Physical exercise in pregnancy: benefits, risks and prescription. J Perinat Med. 2021 Sep 6.

102. Sivan E, Homko CJ, Chen XH, Reece EA, Boden G. Effect of insulin on fat metabolism during and after normal pregnancy. Diabetes. 1999;48(4):834-8.

103. Zhang CL, Ning Y. Effect of dietary and lifestyle factors on the risk of gestational diabetes: review of epidemiologic evidence. American Journal of Clinical Nutrition. 2011;94(6):1975S-9S.

104. Pettit D, Bennett PH, Knowler WC, et al. Gestational diabetes mellitus and impaired glucose tolerance during pregnancy: long-term effects on obesity and glucose intolerance in the offspring. Diabetes Care 1985;34:119–22.

105. Horvath K, Koch K, Jeitler K, et al. Effects of treatment in women with gestational diabetes mellitus: systematic review and meta-analysis. BMJ 2010;340:1395.

106. Carolan-Olah MC. Educational and intervention programmes for gestational diabetes mellitus (GDM) management: an integrative review. Collegian 2016;23:103–14.

107. Sanabria-Martínez G, García-Hermoso A, Poyatos-León R, Álvarez-Bueno C, Sánchez-López M, Martínez-Vizcaíno V. Effectiveness of physical activity interventions on preventing gestational diabetes mellitus and excessive maternal weight gain: a meta-analysis. BJOG. 2015 Aug;122(9):1167-74.

108. Brown J, Ceysens G, Boulvain M. Exercise for pregnant women with gestational diabetes for improving maternal and fetal outcomes. Cochrane Database Syst Rev. 2017 Jun 22;6(6):CD012202

109. Shepherd E, Gomersall JC, Tieu J, Han S, Crowther CA, Middleton P. Combined diet and exercise interventions for preventing gestational diabetes mellitus. Cochrane Database Syst Rev. 2017 Nov 13;11(11):CD010443.

110. Choi J, Fukuoka Y, Lee JH. The effects of physical activity and physical activity plus diet interventions on body weight in overweight or obese women who are pregnant or in postpartum: a systematic review and meta analysis of randomized controlled trials. Preventive Medicine. 2013;56(6):351-64.

111. ACOG - American College of Obstetricians and Gynecologists. Obesity and Pregnancy. ACOG Committee opinion Nº 549. American College of Obstetricians and Gynecologists. Obstet Gynecol 2013; 121:213-7.

112. Mottola MF, Giroux I, Gratton R, et al. Nutrition and exercise prevent excess weight gain in overweight pregnant women. Med Sci Sports Exerc. 2010;42(2):265–72.

113. International Weight Management in Pregnancy (i-WIP) Collaborative Group. Effect of diet and physical activity based interventions in pregnancy on gestational weight gain and pregnancy outcomes: meta-analysis of individual participant data from randomised trials. BMJ. 2017 Jul 19;358:j3119..

114. Say L, Chou D, Gemmill A, et al. Global causes of maternal death: a WHO systematic analysis. Lancet Glob Health 2014;2(6):e323–e333.

115. Gillon TE, Pels A, von Dadelszen P, et al. Hypertensive disorders of pregnancy: a systematic review of international clinical practice guidelines. PLoS One 2014;9(12):e113715.

116. Berzan E, Doyle R, Brown CM. Treatment of preeclampsia: current approach and future perspectives. Curr Hypertens Rep 2014;16(9):473.

117. Mparmpakas D, Goumenou A, Zachariades E, et al. Immune system function, stress, exercise and nutrition profile can affect pregnancy outcome: Lessons from a Mediterranean cohort. Exp Ther Med 2013;5(2):411–8.

118. Chawla S, Anim-Nyame N. Advice on exercise for pregnant women with hypertensive disorders of pregnancy. Int J Gynaecol Obstet. 2015 Mar;128(3):275-9.

119. Abu MA, Abdul Ghani NA, Lim Pei S, Sulaiman AS, Omar MH, Muhamad Ariffin MH, et al. Do exercises improve back pain in pregnancy? Hormone Molecular Biology & Clinical Investigation. 2017;32(3):1-7.

120. Sklempe Kokic I, Ivanisevic M, Uremovic M, Kokic T, Pisot R, Simunic B. Effect of therapeutic exercises on pregnancy-related low back pain and pelvic girdle pain: Secondary analysis of a randomized controlled trial. Journal of Rehabilitation Medicine. 2017;49(3):251-7.

121. Gavin N, Gaynes BN, Lohr KN. Meltzer-Brody S. Perinatal depression: a systematic review of prevalence and incidence. Obstet Gynecol 2005; 106(5):1071-83.

122. Bennett HA, Einarsson A, Taddio A. Prevalence of depression during pregnancy: systematic review. Obstet Gynecol 2006; 103(4):698-709.

123. Trivedi MH, Greer TL, Grannemann BD, Chambliss HO, Jordan AN: Exercise as an augmentation strategy for treatment of major depression. J Psychiatr Pract 2006; 12:205–13.

124. Padmapriya N, Bernard J, Liang S, Loy S, Shen Z, Kwek K, et al. Association of physical activity and sedentary behavior with depression and anxiety symptoms during pregnancy in a multiethnic cohort of Asian women. Archives of Women's Mental Health. 2016;19(6):1119-28.

125. Yang X, Zhang A, Sayer L, Bassett S, Woodward S. The effectiveness of group-based pelvic floor muscle training in preventing and treating urinary incontinence for antenatal and postnatal women: a systematic review. Int Urogynecol J. 2021 Aug 28.

126. Woodley SJ, Lawrenson P, Boyle R, Cody JD, Mørkved S, Kernohan A, Hay-Smith EJC. Pelvic floor muscle training for preventing and treating urinary and faecal incontinence in antenatal and postnatal women. Cochrane Database Syst Rev. 2020 May 6;5(5):CD007471

127. Szumilewicz A, Dornowski M, Piernicka M, Worska A, Kuchta A, Kortas J, . . . Jastrz•'A5bski Z. High-Low Impact Exercise Program Including Pelvic Floor Muscle Exercises Improves Pelvic Floor Muscle Function in Healthy Pregnant Women – A Randomized Control Trial. Frontiers in Physiology. 2019, 9, 1867.

128. Schauberger CW, Rooney BL, Goldsmith L, Shenton D, Silva PD, Schaper A. Peripheral joi laxity increases in pregnancy but does not correlate with serum relaxin levels. American Journal Of Obstetrics And Gynecology. 1996;174(2):667-71.

129. Dumas GA, Reid JG. Laxity of knee cruciate ligaments during pregnancy. The Journal of Orthopaedic and Sports Physical Therapy. 1997;26(1):2-6.

130. Ebi KL, Capon A, Berry P, Broderick C, de Dear R, Havenith G, Honda Y, Kovats RS, Ma W, Malik A, Morris NB, Nybo L, Seneviratne SI, Vanos J, Jay O. Hot weather and heat extremes: health risks. Lancet. 2021 Aug 21;398(10301):698-708.

131. Dervis S, Dobson KL, Nagpal TS, Geurts C, Haman F, Adamo KB. Heat loss responses at rest and during exercise in pregnancy: A scoping review. J Therm Biol. 2021 Jul;99:103011.

132. Branco M, Santos-Rocha R, Vieira F. Biomechanics of gait during pregnancy. Scientific World Journal. 2014;2014:527940.

133. Patterson R, McNamara E, Tainio M, de Sa TH, Smith AD, Sharp SJ, et al. Sedentary behaviour and risk of all-cause, cardiovascular and cancer mortality, and incident type 2 diabetes: a systematic review and dose response meta analysis. Eur J Epidemiol. 2018;33(9):811–29.

134. Fazzi C, Saunders DH, Linton K, Norman JE, Reynolds RM. Sedentary behaviours during pregnancy: a systematic review. Int J Behav Nutr Phys Act. 2017 Mar 16;14(1):32.

135. Barone Gibbs B, Jones MA, Jakicic JM, Jeyabalan A, Whitaker KM, Catov JM. Objectively Measured Sedentary Behavior and Physical Activity Across 3 Trimesters of Pregnancy: The Monitoring Movement and Health Study. J Phys Act Health. 2021 Jan 28;18(3):254-61.

136. Di Fabio DR, Blomme CK, Smith KM, Welk GJ, Campbell CG. Adherence to physical activity guidelines in mid-pregnancy does not reduce sedentary time: an observational study. Int J Behav Nutr Phys Act. 2015 Feb 24;12:27.

137. Oviedo-Caro MÁ, Bueno-Antequera J, Munguía-Izquierdo D. Associations of 24-hours activity composition with adiposity and cardiorespiratory fitness: The PregnActive project. Scand J Med Sci Sports. 2020 Feb;30(2):295-302.

138. Canadian Society for Exercise Physiology. Canadian 24-Hour Movement Guidelines: An Integration of Physical Activity, Sedentary Behaviour, and Sleep. 2021. Available at: https://csepguidelines.ca/guidelines/adults-18-64/

139. Kozey-Keadle S, Libertine A, Lyden K, Staudenmayer J, Freedson PS. Validation of wearable monitors for assessing sedentary behavior. Med Sci Sports Exerc. 2011 Aug;43(8):1561-7.

140. Barone Gibbs B, Paley JL, Jones MA, Whitaker KM, Connolly CP, Catov JM. Validity of self-reported and objectively measured sedentary behavior in pregnancy. BMC Pregnancy Childbirth. 2020 Feb 11;20(1):99.

141. Rosenberg DE, Norman GJ, Wagner N, Patrick K, Calfas KJ, Sallis JF. Reliability and validity of the Sedentary Behavior Questionnaire (SBQ) for adults. J Phys Act Health. 2010 Nov;7(6):697-705.

142. Oviedo-Caro MÁ, Bueno-Antequera J, Munguía-Izquierdo D. Measuring Sedentary Behavior During Pregnancy: Comparison Between Self-reported and Objective Measures. Matern Child Health J. 2018 Jul;22(7):968-77.

143. Pajaujiene S, Dabasinskiene L, Santos-Rocha R. Feasibility and pilot study of a postpartum health promotion program for improving women's body composition and active lifestyle. Acta Medica Mediterranea /International Scientific Journal of Clinical Medicine. 2018, 34, 889-99.

144. Saligheh M, McNamara B, Rooney R. Perceived barriers and enablers of physical activity in postpartum women: a qualitative approach. BMC Pregnancy Childbirth. 2016 Jun 2;16(1):131.

145. Makama M, Awoke MA, Skouteris H, Moran LJ, Lim S. Barriers and facilitators to a healthy lifestyle in postpartum women: A systematic review of qualitative and quantitative studies in postpartum women and healthcare providers. Obes Rev. 2021 Apr;22(4):e13167.

146. Evenson KR, Mottola MF, Owe KM, Rousham EK, Brown WJ, P. Summary of International for Physical Activity Following Pregnancy. Obstet Gynecol Surv 2014 Jul; 69(7): 407–14.

147. Leon-Larios F, Corrales-Gutierrez I, Casado-Mejía R, Suarez-Serrano C.Influence of a pelvic floor training programme to prevent perineal trauma: A quasi-randomised controlled trial. Midwifery 2017 Jul;50:72-7.

148. Mottola MF. Exercise in the postpartum period: practical applications. Curr Sports Med Rep. 2002 Dec;1(6):362-8.

149. Bø K, Stuge B, Hilde G. Specific musculoskeletal adaptations in pregnancy: pelvic floor, pelvic girdle and low back pain. Implications for physical activity and exercise In Santos-Rocha, Editor, Exercise and Physical Activity during Pregnancy and Postpartum. Evidence-Based Guidelines. 2 ed. Switzerland: Springer International Publishing; 2022, Ch.6.

150. Bø K, Berghmans B, Mørkved S, van Kampen M. Evidence-based Physical Therapy for the Pelvic Floor. Bridging Science and Clinical Practice. Elsevier, 2007, 435 p.

151. Zourladani A, Zafrakas M, Chatzigiannis B, Papasozomenou P, Vavilis D, Matziari C. The effect of physical exercise on postpartum fitness, hormone and lipid levels: a randomized controlled trial in primiparous, lactating women. Arch Gynecol Obstet. 2015 Mar;291(3):525-30.

152. Carter T, Bastounis A, Guo B, Jane Morrell C. The effectiveness of exercise-based interventions for preventing or treating postpartum depression: a systematic review and meta-analysis. Arch Womens Ment Health. 2019 Feb;22(1):37-53.

153. Davenport MH, McCurdy AP, Mottola MF, Skow RJ, Meah VL, Poitras VJ, Jaramillo Garcia A, Gray CE, Barrowman N, Riske L, Sobierajski F, James M, Nagpal T, Marchand AA, Nuspl M, Slater

LG, Barakat R, Adamo KB, Davies GA, Ruchat SM. Impact of prenatal exercise on both prenatal and postnatal anxiety and depressive symptoms: a systematic review and meta-analysis. Br J Sports Med. 2018 Nov;52(21):1376-85.

154. Ko•'A5oma•'A5ska-Bogucka D, Mazur-Bialy AI. Physical Activity and the Occurrence of Postnatal Depression-A Systematic Review. Medicina (Kaunas). 2019 Sep 2;55(9):560.

155. Pritchett RV, Daley AJ, Jolly K. Does aerobic exercise reduce postpartum depressive symptoms? a systematic review and meta-analysis. Br J Gen Pract. 2017 Oct;67(663):e684-e691.

156. Nakamura A, van der Waerden J, Melchior M, Bolze C, El-Khoury F, Pryor L. Physical activity during pregnancy and postpartum depression: Systematic review and meta-analysis. J Affect Disord. 2019 Mar 1;246:29-41.

157. Saligheh M, Hackett D, Boyce P, Cobley S. Can exercise or physical activity help improve postnatal depression and weight loss? A systematic review. Arch Womens Ment Health. 2017 Oct;20(5):595-611.

158. Nascimento SL, Pudwell J, Surita FG, Adamo KB, Smith GN. The effect of physical exercise strategies on weight loss in postpartum women: a systematic review and meta-analysis. Int J Obes (Lond). 2014 May;38(5):626-35.

159. Makama M, Skouteris H, Moran LJ, Lim S. Reducing Postpartum Weight Retention: A Review of the Implementation Challenges of Postpartum Lifestyle Interventions. J Clin Med. 2021 Apr 27;10(9):1891.

160. Woodley SJ, Lawrenson P, Boyle R, Cody JD, Mørkved S, Kernohan A, Hay-Smith EJC. Pelvic floor muscle training for preventing and treating urinary and faecal incontinence in antenatal and postnatal women. Cochrane Database Syst Rev. 2020 May 6;5(5):CD007471.

161. Wu YM, McInnes N, Leong Y. Pelvic Floor Muscle Training Versus Watchful Waiting and Pelvic Floor Disorders in Postpartum Women: A Systematic Review and Meta-analysis. Female Pelvic Med Reconstr Surg. 2018 Mar/Apr;24(2):142-149.

162. Liu N, Wang J, Chen DD, Sun WJ, Li P, Zhang W. Effects of exercise on pregnancy and postpartum fatigue: A systematic review and meta-analysis. Eur J Obstet Gynecol Reprod Biol. 2020 Oct;253:285-95.

163. Lim S, O'Reilly S, Behrens H, Skinner T, Ellis I, Dunbar JA. Effective strategies for weight loss in post-partum women: a systematic review and meta-analysis. Obes Rev. 2015 Nov;16(11):972-87.

164. ACOG – American College of Obstetricians and Gynecologists. Exercise after pregnancy, 2019. Available at: https://www.acog.org/womens-health/faqs/exercise-after-pregnancy

165. Bø K, Artal R, Barakat R, Brown WJ, Davies GAL, Dooley M, Evenson KR, Haakstad LAH, Kayser B, Kinnunen TI, Larsén K, Mottola MF, Nygaard I, van Poppel M, Stuge B, Khan KM; IOC Medical Commission. Exercise and pregnancy in recreational and elite athletes: 2016/17 evidence summary from the IOC Expert Group Meeting, Lausanne. Part 3-exercise in the postpartum period. Br J Sports Med. 2017 Nov;51(21):1516-25.

166. Promotion Santé Suisse. Activité physique et santé pendant et après la grossesse, 2018; French. Avalable at: https://promotionsante.ch/assets/public/documents/fr/5-grundlagen/publikationen/ernaehrung-bewegung/empfehlungen/kinder-und-jugendliche/bewegung/Fiche_d_information_PSCH_-_2018-08_Activite_physique_et_sante_pendant_et_apres_la_grossesse.pdf

REVIEWERS

Prof. SJ (Hanlie) Moss, PhD

Director of Research - Physical Activity, Sport and Recreation

Faculty of Health Sciences, North-West University, South Africa

Hugo Vieira Pereira, PhD

Clinica Exercise Physiologist
Assistant Professor - Universidade Lusófona, Lisbon, Portugal

Integrated researcher - Centro de Investigação em Desporto, Educação Física, Exercício e saúde (CIDEFES), Portugal

Dr. Isabel Corrales Gutiérrez, PhD (Gynecologist)

Fetal Medicine Unit, University Hospital Virgen Macarena, Seville, Spain

Department os Surgery, University of Seville, Spain

Prof. Ramón Mendoza, PhD

Professor of Developmental and Educational Psychology

Department of social, Developmental and Educational Psycology

Research Group on Health Promotion and Development of Lifestyle across the Life Span

University of Huelva, Spain

Simona Pajaujiene, PhD

Associate Professor at Lithuanian Sports University, Kaunas, Lithuania

Founder and Program Director at ActiveTraining Kaunas, Lithuania

“This book is a perfect motivation tool, like the guide for health professionals, wich helps to prioritize woman’health and wellbeing during pregnancy and postpartum. Woman do not always know if they need or can maintain an active lifestyle, how to exercise safely in pregnancy and after delivery, therefore they need promotion and constantly support not only from exercise specialists but also from healthcare professionals. Heres everyone can find a concentrated information and main advices to keep fit and healthy. I highly recommend to read and use this most recent, verified, and scientifically proven information regarding training during and post-pregnancy!”

Simona Pajaujiene

Promotion of physical activity and exercise during pregnancy and postpartum. Health professionals guide

Funded by:
Swiss-Portuguese Seed Money CCISP – Conselho Coordenador dos Institutos Superiores Politécnicos (Portugal) – HES-SO – Haute École Specialisée de Suisse Occidentale (Switzerland) collaborative research: ACTIVE PREGNANCY - PROMOTING PHYSICAL EXERCISE AND A HEALTHY LIFESTYLE DURING PREGNANCY AND POSTPARTUM.

Promotors:
IPSANTARÉM - Polytechnic Institute of Santarém, Portugal: ESDRM – Sport Sciences School of Rio Maior; ESSS – Health School of Santarém; ESAS – Agrarian School of Santarém and HES-SO - University of Applied Sciences and Arts Western Switzerland: HESAV - School of Health Sciences, Lausanne

Principal Researchers:
Rita Santos Rocha and Jennifer Wegrzyk

POLITÉCNICO DE SANTARÉM
ESCOLA SUPERIOR DE DESPORTO DE RIO MAIOR

CONSELHO COORDENADOR DOS INSTITUTOS SUPERIORES POLITECNICOS

HESAV
HAUTE ÉCOLE DE SANTÉ VAUD

Hes·so
Haute Ecole Spécialisée de Suisse occidentale
Fachhochschule Westschweiz
University of Applied Sciences Western Switzerland
