# Supplementary material for: Impact of in-person versus online supervised multicentre multicomponent prenatal exercise programme on maternal physical activity, fitness and healthy lifestyle: the Active Pregnancy trial SPIRIT 2025-based protocol
Source: BMJ Open Sport Exerc Med. 2025 Jul 11;11(3):e002767. doi: 10.1136/bmjsem-2025-002767 (PMC12248212; doi:10.1136/bmjsem-2025-002767)
Supplement: Abstract translation 1 [file bmjsem-11-3-s003.docx]

Muitos estudos têm promovido os efeitos positivos de um estilo de vida ativo na prevenção e tratamento de complicações relacionadas com a gravidez, bem como na manutenção da aptidão física e da funcionalidade da mulher grávida. A pandemia de COVID-19 acentuou a necessidade de abordagens virtuais relativamente à promoção da saúde; no entanto, poucos estudos examinaram a eficácia destas intervenções. Foram seguidas as diretrizes SPIRIT 2025 para descrever o protocolo do estudo GRAVIDEZ ATIVA, um estudo de intervenção que disponibiliza um programa de exercício físico presencial (IN) e online (ON) para melhorar os parâmetros de atividade física (AF), de aptidão física e de estilo de vida saudável em mulheres grávidas. As mulheres grávidas serão convidadas a participar num programa de exercício específico, supervisionado por fisiologistas do exercício devidamente qualificado/as, em formato presencial (em várias localidades de Portugal) ou online. Todas as participantes terão acesso à intervenção baseada em exercício físico. As participantes serão avaliadas através de testes de terreno de aptidão física e de questionários específicos sobre os parâmetros de AF, aptidão física e estilo de vida, antes de iniciarem o programa de exercício e após completarem 12 semanas de intervenção. A análise estatística irá testar se a intervenção é benéfica na manutenção ou melhoria dos parâmetros em estudo após 12 semanas de intervenção, e irá comparar a eficácia dos dois formatos de intervenção. Serão também analisados subgrupos de idade materna e de volume semanal de atividade física. Variáveis primárias: volume de atividade física, aptidão física relacionada com a saúde e funcionalidade. Variáveis secundárias: parâmetros de estilo de vida saudável. Variáveis adicionais: satisfação com as intervenções e com os recursos educativos (eg, Guia da Gravidez Ativa e canal YouTube @GravidezAtiva-ActivePregnancy). O estudo foi aprovado pela Comissão de Ética do Politécnico de Santarém, foi registado no ClinicalTrials.gov (NCT06954454) e financiado pela Fundação para a Ciência e Tecnologia (2023.14896.PEX). Os resultados serão divulgados através de publicações, conferências e programas de formação.
